# Asymmetric Synthesis of (*R*)‐1‐Alkyl‐Substituted Tetrahydro‐ß‐carbolines Catalyzed by Strictosidine Synthases

**DOI:** 10.1002/anie.201803372

**Published:** 2018-06-21

**Authors:** Desiree Pressnitz, Eva‐Maria Fischereder, Jakob Pletz, Christina Kofler, Lucas Hammerer, Katharina Hiebler, Horst Lechner, Nina Richter, Elisabeth Eger, Wolfgang Kroutil

**Affiliations:** ^1^ Department of Chemistry, Organic und Bioorganic Chemistry University of Graz, NAWI Graz, BioTechMed Graz Heinrichstrasse 28 8010 Graz Austria; ^2^ ACIB GmbH—Austrian Center of Industrial Biotechnology Petersgasse 14 8010 Graz Austria

**Keywords:** asymmetric catalysis, biocatalysis, Pictet–Spengler reaction, strictosidine Synthases, tetrahydro-β-carbolines

## Abstract

Stereoselective methods for the synthesis of tetrahydro‐ß‐carbolines are of significant interest due to the broad spectrum of biological activity of the target molecules. In the plant kingdom, strictosidine synthases catalyze the C−C coupling through a Pictet–Spengler reaction of tryptamine and secologanin to exclusively form the (*S*)‐configured tetrahydro‐ß‐carboline (*S*)‐strictosidine. Investigating the biocatalytic Pictet–Spengler reaction of tryptamine with small‐molecular‐weight aliphatic aldehydes revealed that the strictosidine synthases give unexpectedly access to the (*R*)‐configured product. Developing an efficient expression method for the enzyme allowed the preparative transformation of various aldehydes, giving the products with up to >98 % *ee*. With this tool in hand, a chemoenzymatic two‐step synthesis of (*R*)‐harmicine was achieved, giving (*R*)‐harmicine in 67 % overall yield in optically pure form.

The Pictet–Spengler reaction^1^ allows the synthesis of a large variety of heterocyclic compounds,^2^ and various chemical methods have been established, including many stereoselective approaches.^2^, ^3^, ^4^ It is catalysed in nature by substrate‐pattern‐specific enzymes.^5^ The most prominent and best characterized members of these Pictet–Spenglerases are the norcoclaurine synthases^6^ and the strictosidine synthases (STRs).^7^

In the natural reaction, the STR (EC 4.3.3.2 according to the Enzyme Commission classification) condenses tryptamine (**1**) and secologanin to generate the (*S*)‐configured 1,2,3,4‐tetrahydro‐ß‐carboline (*S*)‐strictosidine (Scheme 1). The latter serves as a central precursor of naturally occurring, biologically active indole alkaloids, thus the enzyme is located in the biosynthetic pathways at the central branching point of more than 2000 monoterpenoid indole alkaloids in higher plants, including some possessing extraordinary medicinal and therapeutic value.^8^ In general STRs have been reported to show a narrow substrate spectrum with respect to the aldehyde reactant, transforming only secologanin and close derivatives in most cases;^7^, ^9^ to the best of our knowledge, only one report indicated that simple non‐natural aliphatic aldehydes might also be accepted.^10^ Therefore, our aim was to investigate the substrate tolerance of STRs for non‐natural aldehydes.

**Scheme 1:**
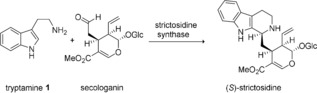
Strictosidine‐synthase‐catalyzed natural Pictet–Spengler condensation of tryptamine **1** and secologanin to give (*S*)‐strictosidine.

Our research focused on four strictosidine synthases originating from *Catharanthus roseus* (CrSTR),^11^ *Ophiorrhiza pumila,* (OpSTR),6e, 12 and *Rauvolfia serpentina* (RsSTR),^13^ as well as its V208A variant (RvSTR),13e which was described to accept an even broader spectrum of amines. We quickly noticed that a significant limitation of using STRs is the low expression level in *E. coli* when using various described constructs, although the activity with secologanin is high (e.g. 31 U mg^−1^ for RsSTR).^14^

Attempts at expressing native sequences of the STRs (Table S1 in the Supporting Information) using various expression plasmids as well as various *E. coli* hosts, chaperones, and expression conditions resulted in no visible overexpression bands detectable by SDS PAGE, although the enzyme preparations allowed us to run the transformation of the natural substrate to completion within 24 hours at 1–2 mm substrate concentration.6b Interestingly, highest activities were found when the STRs were expressed with an N‐terminal His_6_‐tag. However, the use of a non‐natural aldehyde such as isovaleraldehyde **2 a** did not lead to clear product formation independent of the enzyme preparation applied. Additionally, the application of refolding strategies to solubilize inclusion bodies, which were formed in significant amounts, were not successful. The key to success was finally to design *E. coli* optimized DNA sequences devoid of the inherent signal peptide^15^, ^16^ but including an N‐terminal His_6_‐tag, which were expressed using *E. coli* Shuffle T7LysY as the expression host for promoting disulfide bond formation, and performing the expression in TB medium by a careful selection of the expression conditions. This approach finally led to clear detectable enzyme expression and up to 100‐fold improvement in activity per mg cell‐free extract when employing the natural substrates (Figure S1 and Table S6).

Biotransformation of tryptamine with the aliphatic aldehyde isovaleraldehyde **2 a** led to a clear detection of product for all four investigated STRs when using freeze dried cell‐free extracts. Optimization of the reaction conditions (pH, buffer salt, temperature, substrate concentration) enabled successful transformation of tryptamine **1** (10 mm) and isovaleraldehyde **2 a** (50 mm) into the corresponding 1,2,3,4‐tetrahydro‐ß‐carboline **3 a** with 77 % conversion when employing RsSTR within 20 hours (Scheme 2). It has to be noted that 2.5 U_strictosidine_ were employed for these reactions, which equals a catalyst loading of 0.32–6.9 mg of pure STR (applied as 5–65 mg CFE) per 500 μL reaction volume. The STRs showed significant lower activity for the transformation of the non‐natural aldehydes in comparison to the natural substrates, which can probably be attributed to less/no interaction sites between the enzyme and aldehyde substrate. Analysis of the optical purity indicated an essentially optically pure product for RsSTR, RvSTR, and OpSTR (*ee* >98 %, HPLC) while for CrSTR, an *ee* of 88 % was obtained (Table 1, entries 1–4).

**Scheme 2:**
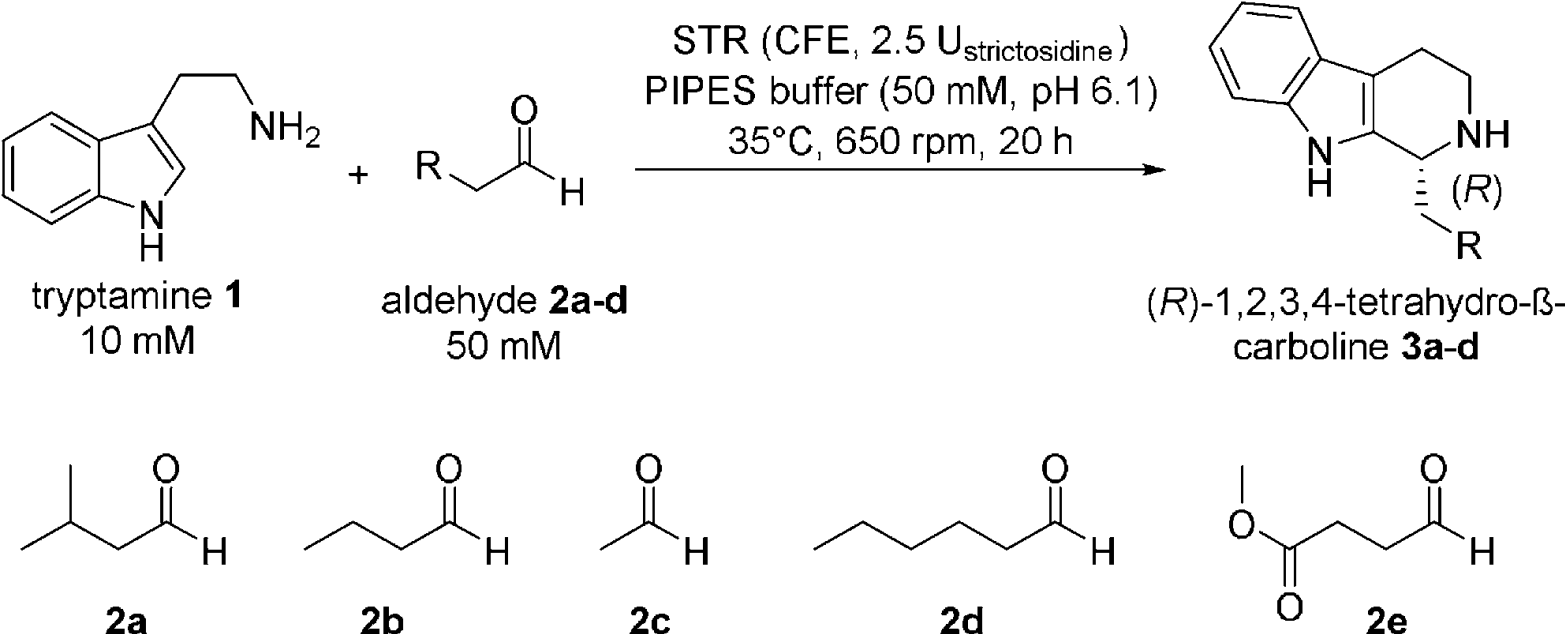
Strictosidine‐synthase‐catalyzed Pictet–Spengler reaction between tryptamine and non‐natural aliphatic low‐molecular‐weight aldehydes.

**Table 1:** Biocatalytic Pictet–Spengler reaction of tryptamine **1** and non‐natural aldehydes **2**.

Entry	Aldehyde 2	STR	Conv. [%]^[a]^	*ee* **3**/**4** [%]^[b]^
1	2 a	CrSTR	12	88 (*R*)
2	2 a	RvSTR	50	>98 (*R*)
3	2 a	RsSTR	77	>98 (*R*)
4	2 a	OpSTR	63	>98 (*R*)
5	2 b	CrSTR	7	61 (*R*)
6	2 b	RvSTR	45	91 (*R*)
7	2 b	RsSTR	42	87 (*R*)
8	2 b	OpSTR	22	88 (*R*)
9	2 c	CrSTR	14	*rac*
10	2 c	RvSTR	23	43 (*R*)
11	2 c	RsSTR	38	*rac*
12	2 c	OpSTR	19	28 (*R*)
13	2 d	CrSTR	4	46 (*R*)
14	2 d	RvSTR	7	82 (*R*)
15	2 d	RsSTR	7	79 (*R*)
16	2 d	OpSTR	8	77 (*R*)
17	2 e	CrSTR	11	>98 (*R*)
18	2 e	RvSTR	66	>98 (*R*)
19	2 e	RsSTR	95	>98 (*R*)
20	2 e	OpSTR	71	>98 (*R*)

[a] Reaction conditions: tryptamine*HCl **1** (10 mm), aldehyde 2 (**2 a**,**b**,**d**: 50 mm, **2 c**: 125 mm), freeze‐dried cell‐free extract of His_6_‐STR/Shuffle T7LysY (2.5 U_strictosidine_), PIPES buffer (0.5 mL, 50 mm, pH 6.1), 35 °C, 650 rpm, 20 h. Product formation and conversion were determined by GC (HP‐5 column). [b] *ee* was determined by HPLC with a chiral stationary phase (**3 a**–**d**: Chiralpak IC; **4**: Chiralcel OD‐H).

Deducing the absolute configuration of the enzymatically formed product **3 a** by comparison of previously reported elution order on a Chiralpak IC column^17^ unexpectedly led to the conclusion that the newly formed chiral centre has to be (*R*)‐configured. This is surprising because the natural reaction leads to the (*S*)‐configured product (*S*)‐strictosidine and the (*S*) configuration has been assumed for product **3 a** in a previous study.^18^ To verify the absolute configuration, a semi‐preparative biocatalytic Pictet–Spengler reaction was performed using RsSTR and the optical rotation of the obtained product **3 a** (85.4 mg, 75 % isolated yield) was measured (>98 % *ee*: [α]20D : +63 (c 1.0, MeOH). Comparison with published optical rotation values at comparable conditions for the (*S*)‐enantiomer^19^ and two reports for the (*R*)‐enantiomer19b, 20 clearly support the idea that the obtained product is (*R*)‐configured, thus (*R*)‐**3 a** was obtained.

In a similar fashion, the non‐branched linear low‐molecular‐weight aldehyde *n*‐butanal **2 b** was transformed giving the corresponding (*R*)‐**3 b** product with up to 91 % *ee* (Entry 6). The smallest possible aldehyde leading to a chiral product, acetaldehyde **2 c**, gave (*R*)‐**2 c** with a clear enantiomeric excess (43 %) when employing RvSTR, while the variant RsSTR led to racemic product (Entry 10 and 11). In all these cases, the (*R*)‐configured product was formed as verified by assigning the absolute configuration by comparison with published optical rotation values (see the Supporting Information).^21^

In the case of hexanal **2 d**, the conversions were low (max 8 % within 20 h), leading to the corresponding β‐carboline **3 d** with up to 82 % *ee* when using RvSTR (entry 14). With all four enzymes investigated, the same enantiomer of the product was in excess. To prove the absolute configuration of the obtained product **3 d**, two independent asymmetric syntheses of optically enriched material according to reported procedures were repeated to have material to identify the elution order of the enantiomers. Both methods, namely using a chiral Brønsted acid4a or a chiral auxiliary,^22^ are reported to give the (*S*)‐enantiomer. Comparison of the elution order of the enantiomers clearly proved that the four enzymes investigated led to the formation of the *R*‐configured (*R*)‐**3 d** (see Figure S20).^23^

To demonstrate the synthetic applicability of the biocatalytic Pictet–Spengler reaction with non‐natural aliphatic aldehydes, the synthesis of the natural alkaloid (*R*)‐harmicine [(*R*)‐**5**] was envisioned. (*R*)‐**5** has been isolated from the Malaysian plant *Kopsia griffithii*, which displays strong anti‐Leishmania activity.^24^ Recently antinociceptive properties have been assigned to **5**.^25^ Consequently, the biocatalytic transformation of commercially available methyl 4‐oxobutanoate **2 e** with tryptamine **1** was performed and led to the formation of the Pictet–Spengler product (*R*)‐**3 e** followed by spontaneous ring closure to give the lactam product (*R*)‐**4** (Scheme 3). The STR from *Rauvolfia serpentina* performed best, giving the product (*R*)‐**4** with 95 % conversion and >98 % *ee*. Again (*R*)‐configuration was assigned through comparison of the optical rotation with reported values (see the Supporting Information). Chemical reduction of the lactam gave the target molecule (*R*)‐harmicine [(*R*)‐**5**] in just two steps in good overall yield (62 %, 2 steps) and perfect *ee* (>98 %). The absolute configuration of (*R*)‐harmicine was confirmed through optical rotation [[α]20D =+87.9 (*c*=0.5, CHCl_3_); Ref. 26c. (*R*)‐isomer, [α]20D =+101.9 (*c*=0.5, CHCl_3_)].

**Scheme 3:**
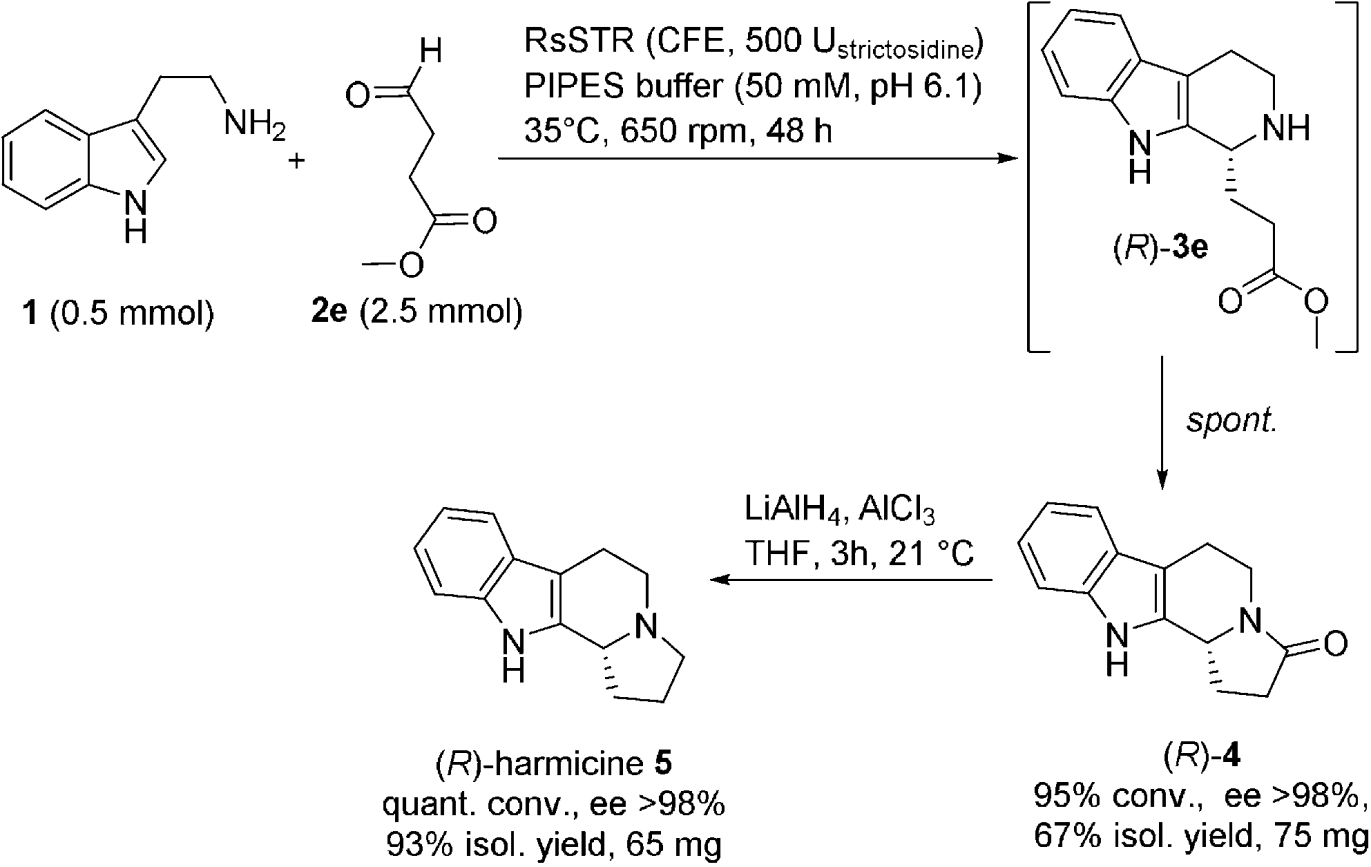
Chemoenzymatic synthesis of (*R*)‐harmicine in two steps starting from commercial substrates.

A few asymmetric synthetic routes to (*R*)‐harmicine [(*R*)‐**5**] have been reported.^26^ To the best of our knowledge, the approach presented here, which enables the synthesis of (*R*)‐**5** in optically pure form in just two steps from commercial substrates, is the shortest and highest yielding sequence reported to date.

Although optimization of the expression conditions to produce sufficient enzyme enabled actual preparative transformations, further optimization of the biocatalysts, for example, by enzyme engineering, is required to reduce the amount of catalyst needed even further.

In conclusion, it has been shown that strictosidine synthases can be used for preparative transformations of low‐molecular‐weight aldehydes, leading unexpectedly to the corresponding (*R*)‐configured products, which is counterintuitive since the natural transformation with the aldehyde secologanin leads to the (*S*)‐configured product. Consequently, the natural alkaloid (*R*)‐harmicine was synthesized with the developed method in just two steps with 62 % yield of isolated product in optically pure form. The presented asymmetric biocatalytic method extends the biocatalytic toolbox for the preparation of chiral amines^27^ through biocatalytic C−C bond formation.^28^

## Experimental Section

Preparative example for the biocatalytic synthesis of the precursors (*R*)‐**4** for (*R*)‐harmicine: Freeze dried cell free extracts of recombinant His_6_‐RsSTR overexpressed in *E. coli*/Shuffle T7LysY (500 U_strictosidine_) were dissolved in a PIPES–tryptamine*HCl buffer system [50 mL, 50 mm PIPES, 10 mm tryptamine*HCl 1*HCl, pH 6.1] in 150 mL Erlenmeyer flasks without baffles. The reaction was started by the addition of methyl 4‐oxobutanoate **2 e** (75 mg, 0.33 mmol, final concentration: 50 mm). The mixture was incubated for 48 h at 470 rpm at 35 °C, then quenched by the addition of aq. NaOH (5 mL, 10 N) and extracted with ethyl acetate (3×100 mL). The combined organic phase was dried over Na_2_SO_4_ and the solvent was evaporated under reduced pressure. Product (*R*)‐**4** was obtained as a white solid (75.4 mg, 67 %).

## Conflict of interest

The authors declare no conflict of interest.

## Supporting information

As a service to our authors and readers, this journal provides supporting information supplied by the authors. Such materials are peer reviewed and may be re‐organized for online delivery, but are not copy‐edited or typeset. Technical support issues arising from supporting information (other than missing files) should be addressed to the authors.

Supplementary
