# Supplementary material for: Asymmetric Synthesis of (R)‐1‐Alkyl‐Substituted Tetrahydro‐ß‐carbolines Catalyzed by Strictosidine Synthases
Source: Angew Chem Int Ed Engl. 2018 Jun 21;57(33):10683–7. doi: 10.1002/anie.201803372 (PMC6146909; doi:10.1002/anie.201803372)
Supplement: Supplementary file 1 — Supplementary [file ANIE-57-10683-s001.pdf]

Angewandte Chemie
Eine Zeitschrift der Gesellschaft Deutscher Chemiker

Supporting Information

Asymmetric Synthesis of (*R*)-1-Alkyl-Substituted Tetrahydro-ß-carbolines Catalyzed by Strictosidine Synthases

*Desiree Pressnitz, Eva-Maria Fischereder, Jakob Pletz, Christina Kofler, Lucas Hammerer, Katharina Hiebler, Horst Lechner, Nina Richter, Elisabeth Eger, and Wolfgang Kroutil**

anie_201803372_sm_miscellaneous_information.pdf

Supporting Information

Table of Contents

General	page 2
Origin, DNA and Amino Acid Sequences of STRs	page 3
Cloning and Expression of STRs	page 5
Biotransformations	page 9
Preparative transformations	page 11
Chemoenzymatic Synthesis of (*R*)-harmicine (**5**)	page 14
Synthesis of references	page 15
Analytical	page 22
Spectra	page 29

General

All chemicals were purchased from Sigma Aldrich, Acros Organics, Alfa Aesar, Oxoid, Carbosynth, TCI Europe, Strem Chemicals or Roth and used as received; solvents were obtained from Roth and Chem-Lab NV. STR genes were synthesized and codon-optimized for E. coli by Life Technologies. Primers were synthesized by Eurofins Genomics. Expression vectors and E. coli strains were purchased from Novagen or Invitrogen. Ni-NTA superflow was purchased from Qiagen. Restriction enzymes were from New England Biolabs. All moisture or air sensitive operations were conducted under dry argon in heat dried glassware. Column chromatography was performed on silica gel 60 from Merck with particle sizes 40-63 μm.

^{1}H- and ^{13}C-NMR spectra were recorded on a Bruker AVANCE III 300 spectrometer (^{1}H: 300.13 MHz; ^{13}C: 75.47 MHz) with autosampler. Chemical shifts were referenced to the residual proton and carbon signal of the deuterated solvent ($CDCl_3$: δ = 7.26 ppm (^{1}H), 77.16 ppm (^{13}C)). Chemical shifts δ are given in ppm (parts per million) and coupling constants *J* in Hz (Hertz). Signal multiplicities are abbreviated as s (singlet), d (doublet), dd (doublet of doublet), t (triplet) and m (multiplet). Deuterated solvents for nuclear resonance spectroscopy were purchased from Roth.

GC-MS measurements were performed on an Agilent 7890A GC system, equipped with an Agilent 5975C mass-selective detector (EI 70 eV) and a HP-5-MS column (30 m x 0.25 mm x 0.25 μm film) using He at a flow rate of 0.5 mL/min. Temperature program (Method A): 100 °C, hold 0.5 min, 10 °C/min 300 °C, hold 2 min, inlet temperature 250 °C. GC-FID measurements were performed on an Agilent 7890A GC system, equipped with an FID detector and a HP-5 column (30 m x 0.32 mm x 0.25 μm film) using He at a total flow rate of 35 mL/min. Temperature program: 100 °C, hold 0.5 min, 10 °C/min 300 °C, inlet temperature 300 °C, split ratio 15/1. Chiral and achiral HPLC analysis was performed on a Shimadzu HPLC system using columns and methods as specified below.

The specific optical rotation was determined on a Perkin Elmer Polarimeter 341 with an integrated sodium vapor lamp. The measurements were performed in spectroscopy ($CHCl_3$, acetone), HPLC grade (EtOH, MeOH) or reagent grade solvents (acetone, >99.9%) at the D-line of the sodium light (λ = 589 nm) under non-tempered conditions between 22 °C and 27 °C.

Origin, DNA and amino acid sequences of STRs

Table S1. List of the investigated STRs.

Abbr.	Organism	GenBank[a]	Vector	Restriction site	pEG-Number[d]
RsSTR	*R. serpentina*	CAA44208.1	pET-28a(+)	NdeI, XhoI	pEG 291
RvSTR	*R. serp.* variant V208A	CAA44208.1[b]	pET-28a(+)	NdeI, XhoI	pEG 290
CrSTR	*C. roseus*	CAA43936.1	pET-28a(+)	NdeI, XhoI	pEG 289
OpSTR	*O. pumila*	-[c]	pET-28a(+)	NheI, XhoI	pEG 292

[a] NCBI Reference sequence accession number. [b] Variant of RsSTR designed by Stöckigt and co-workers.[1] [c] Gene sequence containing a C-terminal His_6-tag was taken from publication.[2] [d] Internal plasmid number, for inquiries about plasmids please refer to this number.

STR from *Rauvolfia serpentina* (RsSTR)

Native amino acid sequence and codon-optimized DNA sequence without signal peptide

```
  1 SPILKEILIE APSYAPNSFT FDSTNKGFYT SVQDGRVIKY EGPNSGFVDF AYASPYWNKA
 61 FCENSTDAEK RPLCGRTYDI SYNLQNNQLY IVDCYYHLSV VGSEGGHATQ LATSVDGVPF
121 KWLYAVTVDQ RTGIVYFTDV STLYDDRGVQ QIMDTSDKTG RLIKYDPSTK ETTLLLKELH
181 VPGGAEVSAD SSFVLVAEFL SHQIVKYWLE GPKKGTAEVL VKIPNPGNIK RNADGHFWVS
241 SSEELDGNMH GRVDPKGIKF DEFGNILEVI PLPPPFAGEH FEQIQEHDGL LYIGTLFHGS
301 VGILVYDKKG NSFVSSH*
```

AGCCCGATTCTGAAAGAAATTCTGATTGAAGCACCGAGCTATGCACCGAATAGCTTTACCTTTGATAGCACCAACAAAGGCTT
TTATACCAGCGTTCAGGATGGTCGTGTTATCAAATATGAAGGTCCGAATAGCGGCTTTGTGGATTTTGCCTATGCAAGCCCGT
ATTGGAATAAAGCCTTTTGTGAAAATAGCACCGATGCCGAAAAACGTCCGCTGTGTGGTCGTACCTATGATATTAGCTATAAT
CTGCAGAACAACCAGCTGTATATCGTGGATTGTTATTATCATCTGAGCGTTGTTGGTAGCGAAGGTGGTCATGCAACCCAGCT
GGCAACCAGCGTTGATGGTGTTCCGTTTAAATGGCTGTATGCAGTTACCGTTGATCAGCGTACCGGTATTGTGTATTTTACCGA
TGTTAGCACCCTGTATGACGATCGTGGTGTGCAGCAGATTATGGATACCAGCGATAAAACCGGTCGTCTGATTAAATACGATC
CGAGCACCAAAGAAACCACCCTGCTGCTGAAAGAACTGCATGTTCCGGGTGGTGCAGAAGTTAGCGCAGATAGCAGCTTTGT
TCTGGTTGCCGAATTTCTGAGCCATCAGATTGTGAAATATTGGCTGGAAGGTCCTAAAAAAGGCACCGCAGAAGTTCTGGTTA
AAATTCCGAATCCGGGTAACATTAAACGTAATGCCGATGGTCATTTTTGGGTTAGCAGCAGCGAAGAACTGGATGGTAATAT
GCATGGTCGCGTTGATCCGAAAGGCATTAAATTCGATGAATTTGGCAACATCCTGGAAGTTATTCCGCTGCCTCCGCCTTTTG
CCGGTGAACATTTTGAGCAGATTCAAGAACATGATGGCCTGCTGTATATTGGCACCCTGTTTCATGGTAGCGTTGGTATTCTG
GTGTATGATAAAAAAGGTAACAGCTTTGTGAGCAGCCACTAA

STR from *Rauvolfia serpentina* variant V208A (RvSTR)

Native amino acid sequence and codon-optimized DNA sequence without signal peptide

```
  1 SPILKEILIE APSYAPNSFT FDSTNKGFYT SVQDGRVIKY EGPNSGFVDF AYASPYWNKA
 61 FCENSTDAEK RPLCGRTYDI SYNLQNNQLY IVDCYYHLSV VGSEGGHATQ LATSVDGVPF
121 KWLYAVTVDQ RTGIVYFTDV STLYDDRGVQ QIMDTSDKTG RLIKYDPSTK ETTLLLKELH
181 APGGAEVSAD SSFVLVAEFL SHQIVKYWLE GPKKGTAEVL VKIPNPGNIK RNADGHFWVS
241 SSEELDGNMH GRVDPKGIKF DEFGNILEVI PLPPPFAGEH FEQIQEHDGL LYIGTLFHGS
301 VGILVYDKKG NSFVSSH*
```

AGCCCGATTCTGAAAGAAATTCTGATTGAAGCACCGAGCTATGCACCGAATAGCTTTACCTTTGATAGCACCAACAAAGGCTT
TTATACCAGCGTTCAGGATGGTCGTGTTATCAAATATGAAGGTCCGAATAGCGGCTTTGTGGATTTTGCCTATGCAAGCCCGT
ATTGGAATAAAGCCTTTTGTGAAAATAGCACCGATGCCGAAAAACGTCCGCTGTGTGGTCGTACCTATGATATTAGCTATAAT
CTGCAGAACAACCAGCTGTATATCGTGGATTGTTATTATCATCTGAGCGTTGTTGGTAGCGAAGGTGGTCATGCAACCCAGCT
GGCAACCAGCGTTGATGGTGTTCCGTTTAAATGGCTGTATGCAGTTACCGTTGATCAGCGTACCGGTATTGTGTATTTTACCGA
TGTTAGCACCCTGTATGACGATCGTGGTGTGCAGCAGATTATGGATACCAGCGATAAAACCGGTCGTCTGATTAAATACGATC
CGAGCACCAAAGAAACCACCCTGCTGCTGAAAGAACTGCATGCACCGGGTGGTGCAGAAGTTAGCGCAGATAGCAGCTTTGT
TCTGGTTGCCGAATTTCTGAGCCATCAGATTGTGAAATATTGGCTGGAAGGTCCTAAAAAAGGCACCGCAGAAGTTCTGGTTA
AAATTCCGAATCCGGGTAACATTAAACGTAATGCCGATGGTCATTTTTGGGTTAGCAGCAGCGAAGAACTGGATGGTAATAT

GCATGGTCGCGTTGATCCGAAAGGCATTAAATTCGATGAATTTGGCAACATCCTGGAAGTTATTCCGCTGCCTCCGCCTTTTG
CCGGTGAACATTTTGAGCAGATTCAAGAACATGATGGCCTGCTGTATATTGGCACCCTGTTTCATGGTAGCGTTGGTATTCTG
GTGTATGATAAAAAAGGTAACAGCTTTGTGAGCAGCCACTAA

STR from *Catharanthus roseus* (CrSTR)

Native amino acid sequence and codon-optimized DNA sequence without signal peptide

1 SPILKKIFIE SPSYAPNAFT FDSTDKGFYT SVQDGRVIKY EGPNSGFTDF AYASPFWNKA
61 FCENSTDPEK RPLCGRTYDI SYDYKNSQMY IVDGHYHLCV VGKEGGYATQ LATSVQGVPF
121 KWLYAVTVDQ RTGIVYFTDV SSIHDDSPEG VEEIMNTSDR TGRLMKYDPS TKETTLLLKE
181 LHVPGGAEIS ADGSFVVVAE FLSNRIVKYW LEGPKKGSAE FLVTIPNPGN IKRNSDGHFW
241 VSSSEELDGG QHGRVVSRGI KFDGFGNILQ VIPLPPPYEG EHFEQIQEHD GLLYIGSLFH
301 SSVGILVYDD HDNKGNSYVS S*

AGCCCGATTCTGAAAAAAATCTTTATTGAAAGCCCGAGCTATGCACCGAATGCATTTACCTTTGATAGCACCGATAAAGGCTT
TTATACCAGCGTTCAGGATGGTCGTGTTATCAAATATGAAGGTCCGAATAGCGGCTTTACCGATTTTGCCTATGCAAGCCCGT
TTTGGAATAAAGCCTTTTGTGAAAATAGTACCGACCCGGAAAAACGTCCGCTGTGTGGTCGTACCTATGATATTAGCTATGAT
TATAAAAACAGCCAGATGTATATCGTGGATGGCCATTATCATCTGTGCGTTGTTGGTAAAGAAGGTGGTTACGCAACCCAGCT
GGCAACCAGCGTGCAGGGTGTTCCGTTTAAATGGCTGTATGCAGTTACCGTTGATCAGCGTACCGGTATTGTGTATTTTACCG
ATGTTAGCAGCATCCATGATGATAGTCCGGAAGGTGTTGAAGAAATTATGAATACCAGCGATCGTACCGGTCGTCTGATGAA
ATATGATCCGAGCACCAAAGAAACCACCCTGCTGCTGAAAGAACTGCATGTTCCGGGTGGTGCAGAAATTAGCGCAGATGGT
AGCTTTGTTGTTGTTGCAGAATTTCTGAGCAACCGCATTGTGAAATATTGGCTGGAAGGTCCTAAAAAAGGTAGTGCCGAATT
TCTGGTTACCATTCCGAATCCGGGTAACATTAAACGTAATAGCGACGGTCATTTTTGGGTGAGCAGCAGCGAAGAACTGGAT
GGTGGTCAGCATGGTCGCGTTGTTAGCCGTGGCATTAAATTCGATGGTTTTGGTAATATCCTGCAGGTTATCCCGCTGCCTCCG
CCTTATGAAGGTGAACATTTTGAGCAGATTCAAGAACATGATGGCCTGCTGTATATTGGTAGCCTGTTTCATAGCAGCGTTGG
TATTCTGGTTTATGATGATCATGATAACAAAGGCAACAGCTATGTGAGCAGCTAA

STR from *Ophiorrhiza pumila* (OpSTR)

Native amino acid sequence and codon-optimized DNA sequence without C-terminal His$_6$-tag

1 SPEFFEFIEA PSYGPNAYAF DSDGELYASV EDGRIIKYDK PSNKFLTHAV ASPIWNNALC
61 ENNTNQDLKP LCGRVYDFGF HYETQRLYIA DCYFGLGFVG PDGGHAIQLA TSGDGVEFKW
121 LYALAIDQQA GFVYYVTDVST KYDDRGVQDI IRINDTTGRL IKYDPSTEEV TVLMKGLNIP
181 GGTEVSKDGS FVLVGEFASH RILKYWLKGP KANTSEFLLK VRGPGNIKRT KDGDFWVASS
241 DNNGITVTPR GIRFDEFGNI LEVVAIPLPY KGEHIEQVQE HDGALFVGSL FHEFVGILHN
301 YKSSVDHHQE KNSGGLNASF KEFSSFGS*

AGTCCGGAATTTTTTGAATTTATTGAAGCACCGAGCTATGGTCCGAATGCATATGCCTTTGATAGTGATGGTGAACTGTATGC
AAGCGTTGAAGATGGTCGCATCATCAAATATGATAAACCGAGCAACAAATTTCTGACCCATGCAGTTGCAAGCCCGATTTGG
AATAATGCACTGTGTGAAAATAACACCAACCAGGATCTGAAACCGCTGTGTGGTCGTGTTTATGATTTTGGCTTTCATTATGA
AACCCAGCGCCTGTATATTGCCGATTGTTATTTTGGTCTGGGTTTTGTTGGTCCGGATGGTGGTCATGCAATTCAGCTGGCAAC
CAGCGGTGATGGTGTTGAGTTTAAATGGCTGTATGCACTGGCAATTGATCAGCAGGCAGGTTTTGTTTATGTTACCGATGTTA
GCACCAAATATGACGATCGTGGTGTTCAGGATATCATTCGCATTAATGATACCACCGGTCGCCTGATTAAATACGATCCGAGC
ACCGAAGAGGTTACCGTTCTGATGAAAGGTCTGAATATTCCGGGTGGCACCGAAGTTAGCAAAGATGGTAGCTTTGTTCTGGT
GGGTGAATTTGCAAGCCATCGTATTCTGAAATATTGGCTGAAAGGTCCGAAAGCAAATACCAGCGAATTTCTGCTGAAAGTTC
GTGGTCCGGGTAACATTAAACGTACCAAAGATGGCGATTTTTGGGTTGCAAGCAGCGATAATAATGGTATTACCGTTACACCG
CGTGGTATTCGCTTTGATGAATTTGGTAATATTCTGGAAGTTGTGGCAATTCCGCTGCCGTATAAAGGTGAACATATTGAACA
GGTGCAAGAACATGATGGTGCCCTGTTTGTTGGTAGCCTGTTTCATGAATTTGTGGGCATTCTGCACAACTATAAAAGCAGCG
TTGATCACCACCAAGAAAAAAACAGCGGTGGTCTGAATGCAAGCTTTAAAGAATTTAGCAGCTTTGGCAGCTAA

Cloning and Expression of STRs

Plasmid set up of STRs without signal sequences

The STR genes were codon optimized for expression in *Escherichia coli*. The nucleotides encoding the first amino acids were omitted to remove putative signal peptides to give the nucleotide sequences given above. N-terminal NdeI and C-terminal XhoI restricition sites were added to the sequences of RsSTR, RvSTR and CrSTR. N-terminal NheI and C-terminal XhoI restricition sites were added to the sequence of OpSTR.

DNAs encoding STRs were amplified by polymerase chain reaction (PCR) using STR gene containing pET-28a(+) vectors as template. PCR was performed using Phusion DNA polymerase (Phusion High Fidelity PCR Kit). Primers as well as PCR reaction and cycling conditions used are given in Tables S2-S4.

Table S2. PCR primers.

STR	Primer	Sequence (5'-3')
RsSTR	Forward	ATATCATATGAGCCCGATTCTGAAAGAAATTCTG
	Reverse	ATATCTCGAGTTAGTGGCTGCTCACAAAGCTGTTACCTTT
OpSTR	Forward	TATATGCTAGCAGTCCGGAATTTTTTGAATTTATTGAA
	Reverse	ATATCTCGAGTTAGCTGCCAAAGCTGCTAAATTCTTT
RvSTR	Forward	ATATCATATGAGCCCGATTCTGAAAGAAATTCTG
	Reverse	ATATCTCGAGTTAGTGGCTGCTCACAAAGCTGTTACCTTT
CrSTR	Forward	ATATCATATGAGCCCGATTCTGAAAAAAATCTTTATTGAAAGC
	Reverse	ATATCTCGAGTTAGCTGCTCACATAGCTGTTG

Table S3. Reaction conditions for the amplification of STR DNA *via* PCR.

Component	Volume [µL]	Final concentration
Nuclease free water	12.4	-
5x Phusion HF buffer	4.0	1x
10 mM dNTPs	0.4	200 µM
Primer forward 10 µM	1	0.5 µM
Primer reverse 10 µM	1	0.5 µM
Template 10 µg/mL	1	0.5 µg/mL
Phusion DNA Polymerase 2 U/µL	0.2	0.02 U/µL
Final Volume	20	

Table S4. Cycling conditions for the amplification of STR DNA *via* PCR.

Step	Cycles	Temperature [°C]	Time [sec]
Initial denaturation	1	98	30
Denaturation	25	98	10
Annealing		62	45
Extension		72	60
Final extension	1	72	420
Hold	1	4	∞

PCR products were purified by Qiagen PCR purification Kit. DNA was eluted with EB-buffer (30 µL). The PCR amplicons obtained and the expression plasmids were digested with the corresponding restriction enzymes. Following agarose gel analysis, restricted PCR products and plasmids were purified according the manual QIAquick Gel Extraction Kit. The purified DNA was eluted with nuclease-free water (2 x 25 µL) and stored at -20 °C. The STR encoding DNA-inserts were ligated using T4 DNA Ligase and 10x T4 DNA Ligase buffer. For the ligation a molar insert to vector ratio of 5:1 was chosen, whereby 80 ng of vector were employed. The ligation mixture was incubated for 2 h at room temperature. Subsequently the ligase was denatured at 65 °C for 10 min and the solution was stored at -20 °C.

For restriction control and sequence analysis, STR gene containing plasmids (5 µL) were transformed into Top10 competent cells (50 µL, for details see the manual of the competent cells). Precultures with LB-medium (10 mL) containing the corresponding antibiotics were inoculated with a single colony and incubated for 20 h at 30 °C and 120 rpm. Subsequently, plasmids were isolated using the QIAprep Spin Miniprep Kit and sequenced by LGC Genomics. For restriction control, the plasmids were restricted using the corresponding restriction enzymes and analysed by agarose gel electrophoresis.

Recombinant STR production

The STR-gene containing plasmids (3 µL) were transformed into suitable *E. coli* chemically competent cells (50 µL). Precultures with LB-medium or TB-medium (10 mL) containing the corresponding antibiotics were inoculated with a single colony and incubated for 20 h at 30 °C and 120 rpm. Main cultures (LB-medium or TB-medium) were inoculated with the preculture and incubated at 37 °C and 120 rpm until an OD_{600} of 0.6-0.8 was reached. Protein expression was started by addition of IPTG (0.5 mM) and shaking was continued over night at 20 °C and 120 rpm. The cells were harvested by centrifugation (4000 rpm, 4 °C, 20 min), washed with PIPES buffer (50 mM, pH 6.1 or pH 6.8), resuspended in PIPES buffer and subjected to cell lysis. Cells were disrupted by ultrasonication (2:30 min,

40% amplitude, 2 sec ON, 1 sec OFF, 3 times) centrifuged (6000 rpm, 4 °C, 20 min) and the supernatants as well as the cell pellets were subsequently lyophilized after resuspension in PIPES buffer.

For the SDS page analysis, lyophilized enzyme preparations [50 µg, dissolved in PIPES buffer (50 mM, pH 6.1 or pH 6.8] were used. Two fold Laemmli sample buffer was added to each protein sample in a ratio of 1:1 in order to prepare the denatured sample proteins. The samples were incubated for 5 minutes at 95 °C. The gel was loaded with the samples, as well as with the Precision Plus Protein All Blue standard or the PageRuler Prestained Protein Ladder (5 µL). A voltage of 80 V was applied first; subsequently the voltage was increased to 100 V.

Protein purification

CFEs of STRs were purified by Protino Ni-NTA affinity chromatography with a column bed volume of 1.5 mL (5 mL total column volume). The column was washed with buffer 1 (6 mL, 50 mM PIPES, 10 mM imidazole, pH 7.5). CFE (252 mg) was resuspended in buffer 1 (4 mL) and applied on Ni-NTA column. Flow-through was collected in a Sarstedt-tube and column was subsequently washed with buffer 1 (1.5 mL). The protein was eluted with buffer 2 (6-7 mL, 50 mM PIPES, 200 mM Imidazol, pH 7.5) and 15-20 fractions (each ~250 µL, 4-5 drops) were collected. The protein content was checked qualitatively by Bio-Rad Protein Assay (199 µL of 1:4 diluted dye reagent, 1 µL of fraction). Fractions containing protein were merged and desalinated by a PD-10 column (8.3 mL of Sephadex™ G-25 Medium, GE Healthcare) by eluting the applied protein solution (2.5 mL) with buffer 3 (3.5 mL, 10 mM PIPES, pH 6.1). Desalinated protein solution was frozen with liquid nitrogen and lyophilized.

Table S5. Yield and protein content of purified active STRs.

STR	MW [kDa]	Yield [mg][a]	Protein content [%][b]	Pure Protein [mg]	Yield [mg/L culture]
His_6-CrSTR	37.97	24.0	33.1	7.94	27
His_6-RvSTR	37.52	32.6	40.4	13.2	46
His_6-RsSTR	37.55	40.8	40.4	16.5	57
His_6-OpSTR	38.89	21.7	30.5	6.63	23

[a] Yield of lyophilized purified protein without abstraction of salt content and impurities. [b] Protein content of lyophilized purified protein preparation. Concentration was determined by Bio-Rad Protein Assay.

Concentrations of enzyme preparations were determined by a Bio-Rad Protein Assay. A certain amount [crude extract (20 µg), flow-through (15 µg), wash (15 µg), fractions (10 µg) and purified lyophilized protein (5 µg, 1 µg/µL, dissolved in buffer 3)] was applied with Laemmli sample buffer on the SDS gel after incubation for 5 minutes at 95 °C. The gel was loaded with the samples as well as with the Prestained Protein Ladder Mix (5 µL). To move the proteins into the half of the gel length a voltage of 80 V was applied; subsequently the voltage was increased to 100 V (Figure 1).

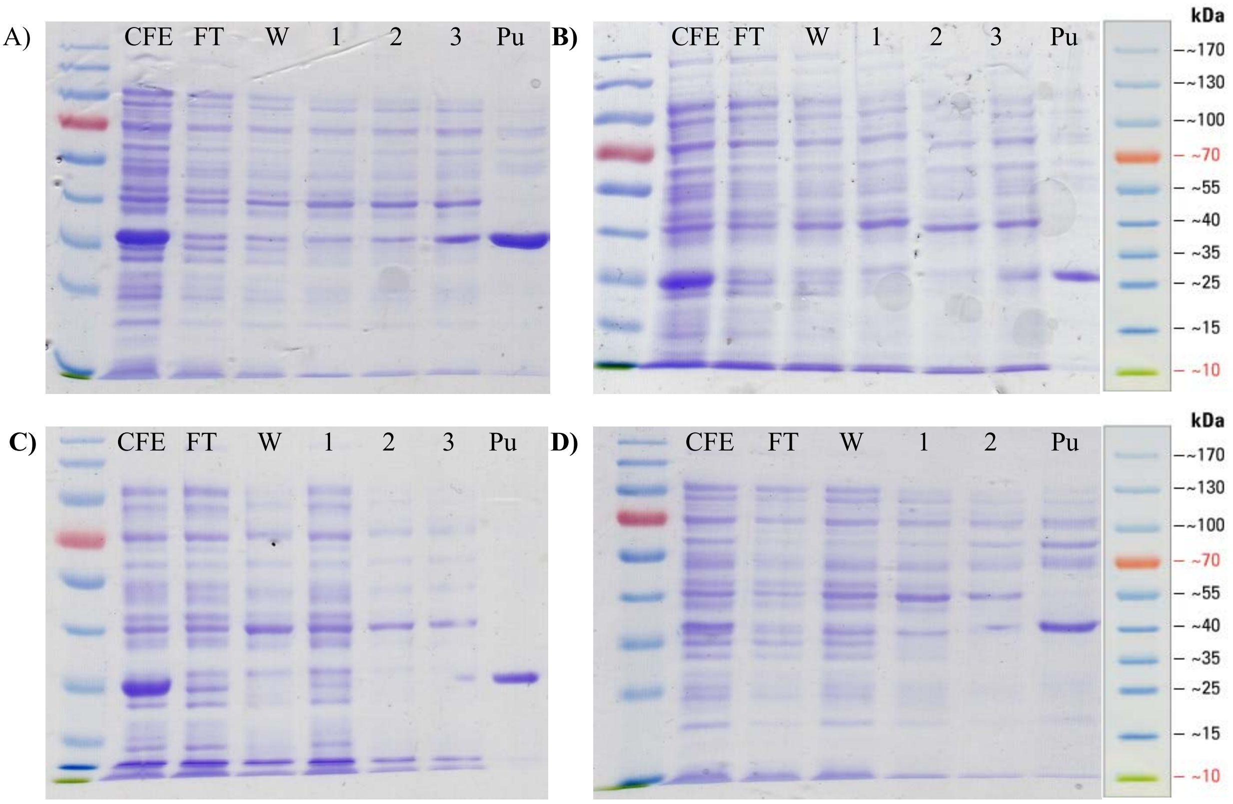

Figure S1. SDS-page. A: His_6-CrSTR (38.0 kDa), B: His_6-RvSTR (37.6 kDa), C: His_6-RsSTR (37.6 kDa), D: His_6-OpSTR (38.9 kDa). Crude extract (CE), flow-through (FT), wash (W), fractions 1, 2, 3 (1, 2, 3), purified enzyme (Pu).

Determination of specific activities

One unit of STR is defined as 1 µmol of (*S*)-strictosidine formed per minute.

The incubation mixtures consisting of a total volume of 500 µL contained lyophilized purified enzyme preparation (0.01 mg) dissolved in PIPES buffer (50 mM, pH 6.1), 2 mM secologanin and 1 mM tryptamine*HCl (**1**). The mixtures were incubated at 35 °C and 450 rpm for a given time (1 min, 2.5 min, 5 min, 10 min, 20 min, 1 h) on an orbital shaker in vertical position. The reaction was terminated by addition of MeOH (500 µL). Then aqueous

ammonium formate solution (500 µL, 30 mM, adjusted to pH 2.8 with formic acid) was added to precipitate the PIPES. The samples were centrifuged and subjected to HPLC analysis.

For the determination of conversions peak areas of strictosidine and tryptamine detected at 254 nm were calculated using the following equation:

$$conversion\ (strictosidine)\ [\%] = \frac{n\ (tryptamine) * peak\ area\ (stricosidine) * 100}{(peak\ area\ (strictosidine) + f * peak\ area\ (tryptamine))}$$

Factor *f* was calculated from calibration lines of secologanine and tryptamine (determined in a range of 0.05-1 mM secologanin and 0.05-2 mM for tryptamine):

$$f = \frac{slope\ calibration\ (secologanin)}{slope\ calibration\ (tryptamine)}$$

Table S6. Activities of the CFE and specific activities of purified His_6-tagged, soluble STRs.

STR	Initial expression<br>Activity<br>[U/mg CFE] [a]	Optimized expression<br>Activity<br>[U/mg CFE][a]	Specific activity<br>[U/mg]
His_6-RsSTR	0.022	0.334	4.05
His_6-RvSTR	0.007	0.038	0.36
His_6-CrSTR	0.005	0.505	7.84
His_6-OpSTR	0.017	0.056	0.80

[a] Soluble, lyophilized fractions (referred to as CFE = cell free extracts) were applied. Reactions were quenched at various time points. Activities were determined via initial rates of the biotransformation reaction using the slope in the linear initial rate period.

BIOTRANSFORMATIONS

Biotransformations employing natural substrates

The incubation mixtures consisting of a total volume of 500 µL contained lyophilized cells or CFE (20 mg) dissolved in PIPES buffer (50 mM, pH 6.8), 2 mM secologanin and 1 mM tryptamine*HCl (**1**). The mixtures were incubated at 28 °C and 450 rpm for 24 h on an orbital shaker in vertical position. The reaction was terminated by addition of MeOH (500 µL). Then aqueous ammonium formate solution (500 µL, 30 mM, adjusted to pH 2.8 with formic acid) was added to precipitate the PIPES. Samples were centrifuged and supernatants were subjected to HPLC analysis.

Activity assay of CFE

The incubation mixtures consisting of a total volume of 500 µL contained lyophilized CFE (0.1 mg) dissolved in PIPES buffer (50 mM, pH 6.8), 2 mM secologanin and 1 mM tryptamine*HCl (**1**). The mixtures were incubated at 28 °C and 450 rpm for a given time (2.5 min, 5 min, 10 min, 20 min and 1 h) on an orbital shaker in vertical position. The reaction was terminated by addition of MeOH (500 µL). Then aqueous ammonium formate solution (500 µL, 30 mM, adjusted to pH 2.8 with formic acid) was added to precipitate the PIPES. Samples were centrifuged and supernatants were subjected to HPLC analysis.

Biotransformation reaction employing non-natural aldehydes under optimized conditions
Freeze dried CFEs of recombinant His_6-STR obtained from expression in *E. coli* Shuffle T7LysY (for determination of stereoselectivity: 20 mg lyophilized CFE, for determination of conversion 2.5 Units) were dissolved in an aqueous PIPES - tryptamine*HCl buffer system [500 µL, 50 mM PIPES, 10 mM tryptamine*HCl (**1**), pH 6.1] and aldehyde [50 mM, exception: acetaldehyde (**2c**) 125 mM due to its high volatility] was added. The mixtures were incubated for 24 h on an orbital shaker in vertical position at 650 rpm and 35 °C. The reaction was quenched by the addition of aqueous NaOH solution (100 µL, 10 N) and extracted with ethyl acetate (2 x 500 µL). The combined organic phase was dried over Na_2SO_4 and subjected to GC-FID analysis.

Preparative transformations

Reactions were performed in 150 mL Erlenmeyer flasks without baffles (in case of 200-fold scale two Erlenmeyer flasks were used). Freeze dried CFEs of recombinant His_6-STR expressed in *E. coli* Shuffle T7LysY (500 Units) were dissolved in an aqueous PIPES - tryptamine*HCl buffer system [50 mL (100 x upscale) or 100 mL (200x scale), 50 mM PIPES, 10 mM tryptamine*HCl (**1**), pH 6.1] and aldehyde [50 mM, exception: acetaldehyde (**2c**) 125 mM due to its high volatility] was added. The mixture was incubated for 48 h on a shaker at 470 rpm at 35 °C. The reaction was quenched by the addition of aqueous NaOH solution [5 mL (100x scale) or 10 mL (200x scale), 10 N] and extracted with ethyl acetate [3 x 100 mL (100 x scale) or 3 x 200 mL (200x scale)]. The combined organic phase was dried over Na_2SO_4 and solvent was evaporated.

Table S7. Results of isolated THBCs obtained by STR catalyzed biotransformation.

Product	STR	Scale [times][a]	Characteristics	Isolated Yield [%] (mg)	*Ee* [%] [b]
**3a**	His_6-RsSTR	100	yellow oil	75 (85.4)	>98
**3b**	His_6-RsSTR	200	yellow oil	25 (54.5)	90
**3c**	His_6-OpSTR[c]	200	red oil	6 (11.2)	19
**4**	His_6-RsSTR	100	white solid	67 (75.4)[d]	>98

[a] Type of enzyme preparation: soluble lyophilized fractions (referred to as CFE). Reaction conditions: tryptamine*HCl (**1**) (10 mM), aldehyde (50 mM, except for acetaldehyde: 125 mM), CFE of His_6-STR/Shuffle T7LysY (500 Units), PIPES buffer [50 mL (scale: 100x) or 100 mL (scale: 200x), 50 mM, pH 6.1], 35 °C, 470 rpm, 48h. [b] *Ee* was determined by HPLC on a chiral column. [b] n.d. = not determined. [c] Although higher *ee*s were obtained with RvSTR, OpSTR was applied due to higher activity of the CFE. [d] MP: 158-163 °C.

Table S8. Specific optical rotations and corresponding absolute configurations of isolated enantioenriched THBCs.

THBC	$[\alpha]_D^{20}$ (Experimental)	$[\alpha]_D^{20}$ (Literature)	Abs. config.
**(*R*)-3a**	+63° (c=1.0, MeOH) (>98% ee)	-81° [(*S*), c=1.0, MeOH][3]	(*R*)
**(*R*)-3b**	+63 (c=1.0, EtOH) (90% ee)	-73 [(*S*), c=1.0, EtOH][3]	(*R*)
**(*R*)-3c**	+4° (c=0.2, EtOH) (19% ee)	+56 [(*R*), c=2.0, EtOH][4]	(*R*)
**(*R*)-4**	+221° (c=1.0, $CHCl_3$) (>98% ee)	+234° [(*R*), c=1.0, $CHCl_3$][5]	(*R*)

(*R*)-1-Isobutyl-1,2,3,4-tetrahydro-9*H*-pyrido[3,4-*b*]indole ((*R*)-3a)

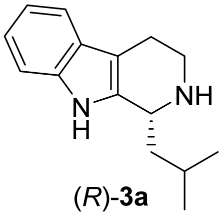

(*R*)-**3a**

Yield: 85.4 mg (374 µmol, 75%) yellow oil; R_f= 0.34 (CH_2Cl_2/MeOH/TEA = 90:9:1); $[\alpha]^{20}_D$ = +63° (c = 1.0, MeOH).

^{1}H-NMR ($CDCl_3$, 300.13 MHz): 7.79 (1H, bs), 7.50 – 7.26 (2H, m), 7.18 – 7.07 (2H, m), 4.15 – 4.10 (1H, m), 3.39 – 3.32 (1H, m), 3.08 - 2.99 (1H, m), 2.78 – 2.73 (2H, m), 2.03 - 1.94 (1H, m), 1.68 – 1.57 (2H, m), 1.03 (3H, d, *J* = 6.6 Hz), 1.01 (3H, d, *J* = 6.6 Hz). ^{13}C-NMR (CD_3OD, 75.47 MHz): 138.3, 130.6, 127.5, 123.4, 120.6, 119.0, 112.4, 107.1, 53.0, 43.1, 42.6, 25.2, 23.9, 21.7, 19.5. GC-MS (Method A): t_R = 16.08 min, >99% purity. *m/z* (rel. int.) = 228 [M$^+$] (9), 198 (1), 184 (3), 171 (100), 156 (8), 143 (6), 128 (3), 115 (3), 102 (1), 85 (3), 76 (1), 54 (1), 41 (2).

(*R*)-1-Propyl-2,3,4,9-tetrahydro-1*H*-pyrido[3,4-*b*]indole ((*R*)-3b)

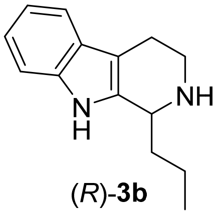

(*R*)-**3b**

Yield: 11.2 mg (52.3 µmol, 6%) red oil; R_f= 0.39 (CH_2Cl_2/MeOH/Et_3N = 90:9:1); $[\alpha]^{20}_D$ = +63° (c = 1.0, EtOH).

^{1}H-NMR (CD_3OD, 300.13 MHz): 7.37 – 7.25 (2H, m), 7.05 - 6.92 (2H, m), 4.06 – 4.03 (1H, m), 3.34 – 3.26 (1H, m), 2.98 – 2.89 (1H, m), 2.82 – 2.65 (2H, m), 2.02 - 1.88 (1H, m), 1.70 – 1.44 (3H, m), 1.00 (3H, t, *J* = 7.2 Hz). ^{13}C-NMR (CD_3OD, 75.47 MHz): 137.7, 136.5, 128.5, 122.0, 119.6, 118.5, 111.8, 108.4, 53.9, 43.6, 37.5, 29.9, 22.7, 19.9, 14.5. GC-MS (Method A): t_R = 15.65 min, >99% purity. *m/z* (rel. int.) = 214 [M$^+$] (11), 184 (3), 171 (100), 154 (8), 141 (7), 128 (3), 115 (5), 102 (1), 85 (5), 76 (2), 54 (1), 39 (1).

(*R*)-1-Methyl-2,3,4,9-tetrahydro-1*H*-pyrido[3,4-*b*]indole ((*R*)-3c)

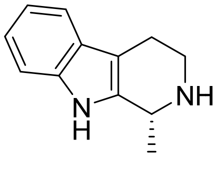

(*R*)-**3c**

Yield: 54.5 mg (293 µmol, 25%) yellow oil; R_f= 0.25 (CH_2Cl_2/MeOH/TEA = 90:9:1), $[\alpha]^{20}_D$ = +4° (c = 0.2, EtOH).

^{1}H-NMR ($CDCl_3$, 300.13 MHz): 8.16 (1H, bs), 7.48 – 7.32 (2H, m), 7.18 – 7.07 (2H, m), 4.28 – 4.26 (1H, m), 3.40 – 3.27 (1H, m), 3.13 – 3.04 (2H, m), 2.80 – 2.78 (2H, m), 1.51 (3H, d, *J* = 6.6 Hz). ^{13}C-NMR ($CDCl_3$, 75.47 MHz): 135.9, 135.8, 127.4, 121.9, 119.6, 118.3, 111.1, 108.1, 48.3, 42.1, 21.9, 20.3. GC-MS (Method A): t_R = 14.00 min, >99% purity. *m/z* (rel. int.) = 186 [M$^+$] (54), 171 (100), 157 (13), 144 (16), 130 (14), 115 (12), 101 (3), 93 (4), 85 (14), 77 (8), 63 (3), 51 (2), 39 (3).

(*R*)-1,2,5,6,11,11b-Hexahydro-3*H*-indolizino[8,7-*b*]indol-3-one ((*R*)-4)

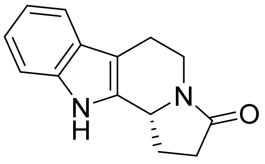

(*R*)-**4**

(Note: The primary product of the enzymatic reaction **3e** dehydrated spontaneously *in vitro* to give the lactam **4**)

Yield: 75.4 mg (333 µmol, 67%) white solid; mp: 158-163 °C; R_f = 0.52 (CH_2Cl_2/MeOH/TEA = 90:9:1); $[\alpha]^{20}_D$ = +221° (c=1.0, $CHCl_3$).

^{1}H-NMR ($CDCl_3$, 300.13 MHz): 8.42 (1H, bs), 7.44 – 7.26 (2H, m), 7.14 – 7.03 (2H, m), 4.90 – 4.85 (1H, m), 4.50 – 4.44 (1H, m), 3.03 - 2.93 (1H, m), 2.86 – 2.71 (2H, m), 2.62 – 2.37 (3H, m), 1.99 – 1.81 (1H, m). ^{13}C-NMR ($CDCl_3$, 75.47 MHz): 173.5, 136.4, 133.4, 126.9, 122.3, 119.9, 118.5, 111.2, 108.2, 54.5, 37.8, 31.8, 25.8, 21.2. GC-MS (Method A): t_R = 19.75 min, >99% purity. *m/z* (rel. int.) = 226 [M$^+$] (100), 207 (2), 197 (6), 182 (8), 169 (33), 156 (9), 143 (8), 128 (5), 115 (9), 98 (2), 87 (2), 77 (3), 63 (2), 51 (2), 39 (2).

Chemoenzymatic Synthesis of (*R*)-Harmicine (5)

Reactions were performed in 150 mL Erlenmeyer flasks without baffles. Freeze dried E. coli CFEs of expressed recombinant His6-RsSTR overexpressed in *E. coli* /Shuffle T7LysY (500 Units) were dissolved in an aqueous PIPES - tryptamine*HCl buffer system [50 mL, 50 mM PIPES, 10 mM tryptamine*HCl (**1**), pH 6.1] and methyl 4-oxobutanoate (**2e**) (75.0 mg, 330 µmol, final concentration: 50 mM) was added. The mixture was incubated for 48 h at 470 rpm at 35 °C. The reaction was quenched by the addition of aqueous NaOH solution (5 mL, 10 N) and extracted with ethyl acetate (3 x 100 mL). The combined organic phase was dried over Na_2SO_4, filtrated and the solvent was evaporated under reduced pressure. (*R*)-1,2,5,6,11,11b-Hexahydro-3*H*-indolizino[8,7-*b*]indol-3-one (*R*)-**4** was obtained as a white solid (75.4 mg, 67%).

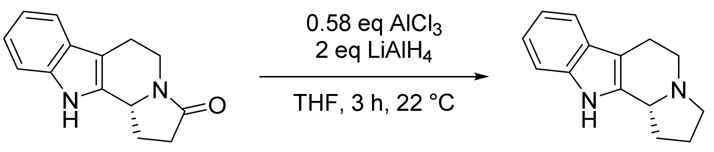

(*R*)-**4** (*R*)-**5**

Scheme S1. Reduction of (*R*)-**4** to (*R*)-harmicine ((*R*)-**5**).

Compound (*R*)-**4** (75.0 mg, 330 µmol)) was subsequently reduced according to literature.[5] The reaction parameters are given in Scheme S1. A flame dried round-bottomed flask was charged with $AlCl_3$ (25.0 mg, 190 µmol, 0.58 eq) and dry THF (2.0 mL) under nitrogen atmosphere. The mixture was cooled at 0 °C with an ice bath and $LiAlH_4$ (25.2 mg, 670 µmol, 2 eq) was added. After stirring 10 min at 0 °C, a solution of lactam (*R*)-**4** (75.0 mg, 330 µmol, 1 eq) in dry THF (1.5 mL) was added *via* canula. The reaction mixture was stirred 30 min at RT, quenched by the addition of saturated aqueous NH_4Cl (50 µL) and poured into saturated aqueous $NaHCO_3$ solution (10 mL). After extraction with ethyl acetate (2 x 7 mL), the organic phase was washed with brine (2 x 7 mL), dried over $MgSO_4$, filtrated and the solvent was removed under reduced pressure. The crude product was purified by column chromatography on silica gel (chloroform/methanol 9:1 v/v) to afford (*R*)-harmicine ((*R*)-**5**) (65.0 mg, 310 µmol) in 93% yield as white solid with 98% *ee*. (m.p. 158-164 °C). Specific optical rotation: $[\alpha]^{20}_D$ = +87.9 (c 0.5, $CHCl_3$).

^{1}H-NMR ($CDCl_3$, 300.13 MHz): 8.29 (1H, bs), 7.48 – 7.30 (2H, m), 7.17 – 7.07 (2H, m), 4.33 – 4.30 (1H, m), 3.37 – 3.30 (1H, m), 3.16 – 3.07 (1H, m), 3.01 - 2.90 (3H, m), 2.75 – 2.67 (1H, m), 2.37 – 2.26 (1H, m), 1.98 – 1.81 (3H, m). ^{13}C-NMR ($CDCl_3$, 75.47 MHz): 136.2, 134.5,

127.2, 121.7, 119.6, 118.2, 111.0, 107.5, 57.4, 49.6, 46.2, 29.7, 23.4, 17.8. GC-MS (Method A): t_R = 16.28 min, >99% purity, *m/z* (rel. int.) = 211 [M^+] (100), 197 (2), 184 (29), 169 (13), 156 (23), 143 (3), 128 (8), 115 (7), 106 (6), 91 (6), 77 (7), 63 (2), 52 (2), 42 (2).

Synthesis of references

Reference compounds were synthesized in a racemic fashion by PSR catalysed either by maleic acid in aqueous medium or by chlorotrimethylsilane in dry pyridine according to literature procedures.[6] Racemic THBC *rac*-**4** was reduced to THBC *rac*-**5** according to literature.[5] Reaction parameters are given in the corresponding schemes. Obtained reference compounds were further used for calibration required for quantitative determination of enzyme catalysed product formation by GC-FID analysis.

***rac*-1-Isobutyl-2,3,4,9-tetrahydro-1*H*-pyrido[3,4-*b*]indol-2-ium chloride (*rac*-3a*HCl)**

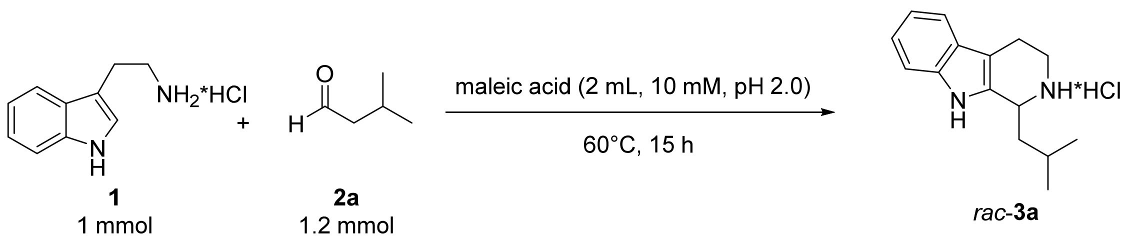

Scheme S2. Maleic acid catalyzed PSR of tryptamine hydrochloride (**1***HCl) and isovaleraldehyde (**2a**).

Yield: 78.5 mg (300 µmol, 30%) colorless solid.

mp = 244–245 °C; R_f = 0.25 (CH_2Cl_2/MeOH/TEA = 90:9:1).

^{1}H-NMR (CD_3OD, 300.13 MHz): 7.48 – 7.35 (2H, m), 7.17 – 7.02 (2H, m), 4.77 – 4.73 (1H, m), 3.76 – 3.68 (1H, m), 3.47 – 3.38 (1H, m), 3.16 – 3.00 (2H, m), 2.11 - 1.81 (3H, m), 1.14 (3H, d, ^3J = 6.4 Hz), 1.09 (3H, d, ^3J = 6.4 Hz). ^{13}C-NMR (CD_3OD, 75.47 MHz): 138.3, 130.5, 127.4, 123.4, 120.6, 119.0, 112.3, 107.1, 53.0, 43.1, 42.6, 25.2, 23.9, 21.6, 19.5. GC-MS (Method A): t_R = 16.20 min, >99% purity, *m/z* (rel. int.) = 228 [M^+] (12), 199 (1), 184 (4), 171 (100), 156 (8), 144 (5), 128 (3), 115 (4), 102 (1), 85 (2), 76 (1), 54 (1), 41 (1).

***rac*-1-Propyl-2,3,4,9-tetrahydro-1*H*-pyrido[3,4-*b*]indol-2-ium chloride (*rac*-3b*HCl)**

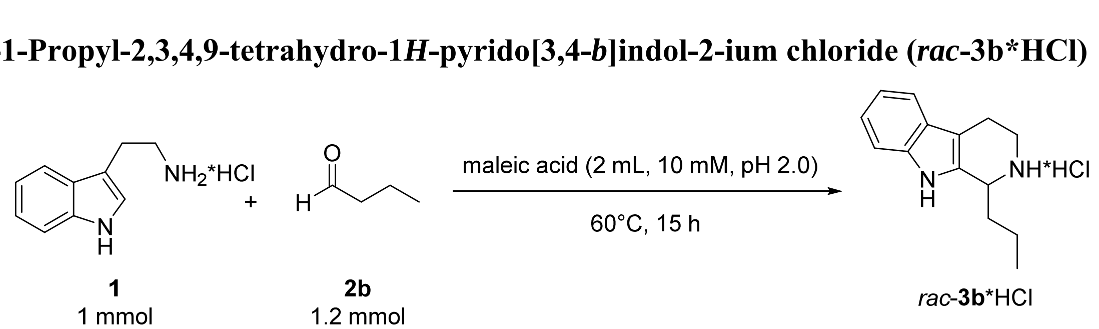

Scheme S3. Maleic acid catalyzed Pictet-Spengler reaction of tryptamine hydrochloride (**1***HCl) and *n*-butanal (**2b**).

Yield: 86.8 mg (350 µmol, 35%) colorless solid.

mp = 243 - 244 °C; R_f = 0.24 (CH_2Cl_2/MeOH/TEA = 90:9:1).

^{1}H-NMR (CD_3OD, 300.13 MHz): 7.48 – 7.35 (2H, m), 7.17 – 7.02 (2H, m), 4.70 – 4.65 (1H, m), 3.75 – 3.68 (1H, m), 3.44 – 3.37 (1H, m), 3.11 – 3.04 (2H, m), 2.26 – 2.18 (1H, m), 1.97 – 1.91 (1H, m), 1.66 – 1.58 (2H, m), 1.09 (3H, t, *J* = 7.2 Hz). ^{13}C-NMR (CD_3OD, 75.47 MHz): 138.3, 130.3, 127.4, 123.4, 120.6, 119.1, 112.3, 107.1, 54.8, 43.1, 35.4, 19.52, 19.48, 14.2. GC-MS (Method A): t_R = 15.75 min, >99% purity, *m/z* (rel. int.) = 214 [M^+] (14), 197 (1), 184 (3), 171 (100), 154 (8), 143 (7), 128 (3), 115 (4), 102 (1), 85 (2), 76 (1), 54 (1), 41 (1).

***rac*-1-Methyl-2,3,4,9-tetrahydro-1*H*-pyrido[3,4-*b*]indole (*rac*-3c)**

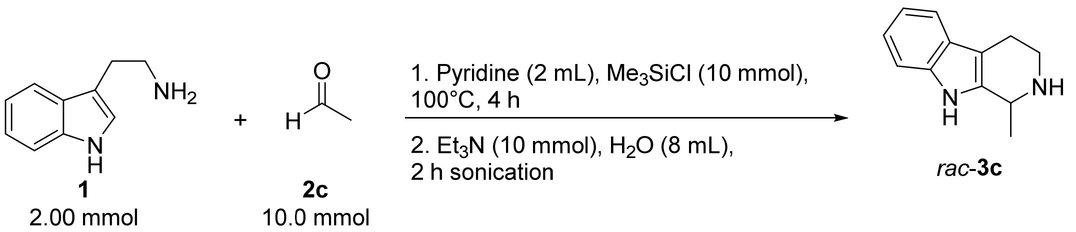

Scheme S4. Maleic acid catalyzed PSR of tryptamine hydrochloride (**1**) and acetaldehyde (**2c**).

Yield: 99.0 mg (530 µmol, 27%) yellow crystals.

mp = 168 – 172 °C; R_f = 0.25 (CH_2Cl_2/MeOH/TEA = 90:9:1).

^{1}H-NMR ($CDCl_3$, 300.13 MHz): 7.51 – 7.30 (2H, m), 7.18 – 7.08 (2H, m), 4.20 – 4.18 (1H, m), 3.42 – 3.34 (1H, m), 3.11 – 3.02 (2H, m), 2.96 – 2.79 (2H, m), 1.94 (1H, bs), 1.46 (3H, d, *J* = 6.6 Hz). ^{13}C-NMR ($CDCl_3$, 75.47 MHz): 137.2, 135.7, 129.9, 127.6, 121.9, 121.6, 119.5, 118.2, 110.9, 108.6, 48.3, 42.9, 22.8, 20.9. GC-MS (Method A): t_R = 14.00 min, >99% purity, *m/z* (rel. int.) = 186 [M^+] (54), 171 (100), 157 (13), 144 (16), 130 (14), 115 (12), 101 (3), 93 (4), 85 (14), 77 (8), 63 (3), 51 (2), 39 (3).

***rac*-1-Pentyl-2,3,4,9-tetrahydro-1*H*-pyrido[3,4-*b*]indol-2-ium chloride (*rac*-3d*HCl)**

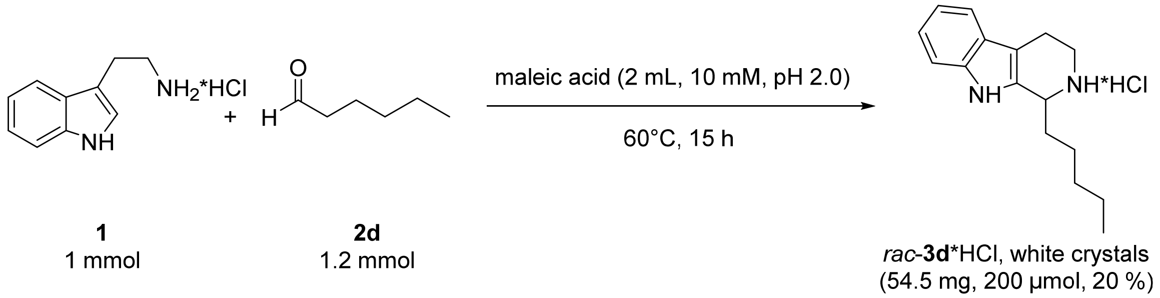

Scheme S5. Maleic acid catalyzed PSR of tryptamine hydrochloride (**1**) and hexanal (**2d**).

Yield: 54.5 mg (200 µmol, 20%) colorless solid.

mp = 241 – 243 °C, R_f = 0.24 (CH_2Cl_2/MeOH/TEA = 90:9:1).

^{1}H-NMR (CD_3OD, 300.13 MHz): 7.48 – 7.35 (2H, m), 7.17 – 7.02 (2H, m), 4.68 – 4.64 (1H, m), 3.75 – 3.68 (1H, m), 3.45 – 3.35 (1H, m), 3.11 - 2.99 (2H, m), 2.33 - 2.22 (1H, m), 2.00 - 1.87 (1H, m), 1.65 - 1.39 (6H, m), 0.97 (3H, t, *J* = 6.9 Hz). ^{13}C-NMR (CD_3OD, 75.47 MHz): 138.3, 130.3, 127.4, 123.4, 120.5, 119.1, 112.3, 107.1, 55.1, 43.1, 33.3, 32.7, 25.9, 23.4, 19.5, 14.3. GC-MS (Method A): t_R = 17.44 min, >99% purity, *m/z* (rel. int.) = 242 [M^+] (8), 212 (1),

195 (1), 184 (2), 171 (100), 156 (5), 144 (6), 128 (3), 115 (4), 102 (1), 85 (2), 76 (1), 54 (1), 41 (1).

***rac*-1,2,5,6,11,11b-Hexahydro-3*H*-indolizino[8,7-*b*]indol-3-one (*rac*-4)**

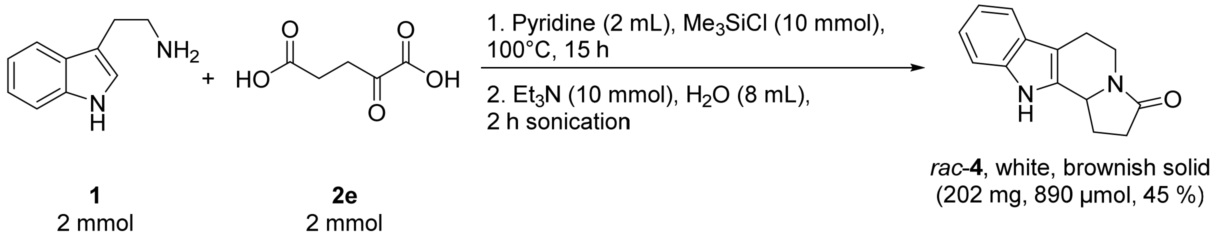

Scheme S6. Me_3SiCl catalyzed PSR of tryptamine (**1**) and 2-oxoglutaric acid (**2e**).

Yield: 202 mg (890 µmol, 45%) off-white solid; mp = 235–240 °C; R_f = 0.50 (CH_2Cl_2/MeOH/TEA = 90:9:1).

^{1}H-NMR (DMSO-d_6, 300.13 MHz): 11.04 (1H, bs), 7.41 – 7.32 (2H, m), 7.09 - 6.95 (2H, m), 4.93 – 4.88 (1H, m), 4.30 – 4.24 (1H, m), 3.01 - 2.93 (1H, m), 2.78 – 2.44 (3H, m), 2.34 – 2.23 (1H, m), 1.85 – 1.76 (1H, m). ^{13}C-NMR (DMSO-d_6, 75.47 MHz): 172.3, 136.1, 134.6, 126.4, 121.0, 118.6, 117.9, 111.2, 105.9, 53.6, 36.9, 31.1, 25.5, 20.8. GC-MS (Method A): t_R = 19.73 min, >99% purity, *m/z* (rel. int.) = 226 [M$^+$] (100), 207 (12), 197 (8), 182 (11), 169 (34), 156 (10), 143 (9), 128 (6), 115 (11), 99 (4), 87 (4), 73 (7), 63 (3), 52 (3), 39 (2).

***rac*-Harmicine (*rac*-5)**

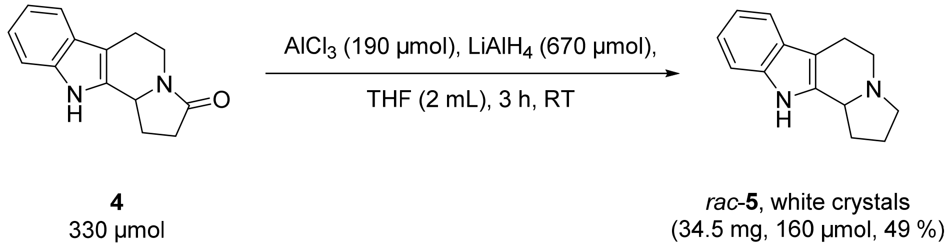

Scheme S7. Reduction of THBC **4** to racemic harmicine (*rac*-**5**).

Yield: 34.5 mg (160 µmol, 49%) colorless crystals; mp = 164-167 °C, R_f = 0.29 (CH_2Cl_2/MeOH/TEA = 90:9:1).

^{1}H-NMR ($CDCl_3$, 300.13 MHz): 8.61 (1H, bs), 7.48 – 7.33 (2H, m), 7.17 – 7.06 (2H, m), 4.37 – 4.33 (1H, m), 3.36 – 3.29 (1H, m), 3.18 – 3.09 (1H, m), 3.05 - 2.90 (3H, m), 2.78 – 2.70 (1H, m), 2.38 – 2.28 (1H, m), 1.98 – 1.82 (3H, m). ^{13}C-NMR ($CDCl_3$, 75.47 MHz): 136.3, 134.1, 127.1, 121.7, 119.5, 118.2, 111.1, 107.3, 57.5, 49.7, 46.2, 29.7, 23.3, 17.8. GC-MS (Method A): t_R = 16.28 min, >99% purity, *m/z* (rel. int.) = 211 [M$^+$] (100), 196 (2), 184 (29), 168 (13), 156 (23), 143 (3), 128 (8), 115 (7), 103 (6), 91 (6), 77 (7), 63 (2), 52 (2), 42 (2).

Synthesis of chiral reference materials

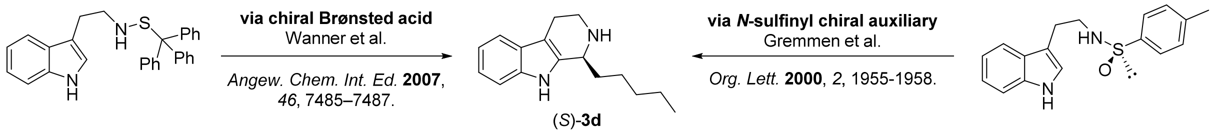

Scheme S8. Synthesis of (*S*)-**3d** as chiral reference material via two different published routes.

(*N*)-(Triphenylmethanesulfenyl)tryptamine

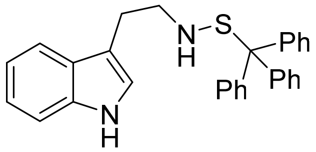

A 25 mL round-bottom flask with magnetic stirring bar was charged with tryptamine (263 mg, 1.61 mmol, 1 eq) followed by CH_2Cl_2 (12 mL) and was cooled to 0 °C with an ice bath. The mixture was vigorously stirred and 20 wt% aqueous NaOH solution (4 mL) was slowly added. Triphenylmethanesulfenyl chloride (500 mg, 1.61 mmol, 1 eq) was added in small portions over a period of 8 min and the mixture was kept stirring in the ice bath. Full conversion of the starting material was detected after 70 min by TLC (cyclohexane/EtOAc = 4:1, v/v), the mixture was transferred into a separating funnel, diluted with distilled H_2O (5 mL) and CH_2Cl_2 (~15 mL) until all solid parts had dissolved. The phases were separated and the aqueous phase was extracted with CH_2Cl_2 (5 x 10 mL). The combined organic layers were dried over Na_2SO_4, filtrated, reduced in vacuum and the residue was adsorbed on silica gel (CH_2Cl_2). The product was purified by column chromatography (silica gel, size: 27 x 1.8 cm, cyclohexane/EtOAc = 85:15 (v/v)).[7]

Yield: 632 mg (1.45 mmol, 90%) light-yellow, very viscous liquid.

R_f = 0.40 (cyclohexane/EtOAc = 4:1 (v/v)) (254 nm, CAM: black).

^{1}H- and ^{13}C-NMR spectra were in accordance with reported data.[7]

(*S*)-1-Pentyl-2,3,4,9-tetrahydro-1*H*-pyrido[3,4-*b*]indole ((*S*)-3d)

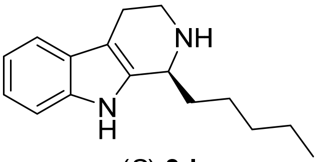

(*S*)-**3d**

A 4 mL screw-cap vial with magnetic stirring bar was charged with BHT (2.2 mg, 10.0 µmol, 0.1 eq), (*R*)-3,3′-bis[3,5-bis(trifluoromethyl)phenyl]-1,1′-binaphthyl-2,2′-diyl hydrogenphosphate (3.9 mg, 5.00 µmol, 0.05 eq), 1 mL dry toluene, (*N*)-(triphenylmethanesulfenyl)tryptamine (43.5 mg, 100 µmol, 1 eq) and powdered 3 Å molecular sieves (150 mg). The mixture was cooled to 0 °C, distilled hexanal (36.7 µL,

30.0 mg, 300 µmol, 3 eq) were added and the mixture was stirred at 0 °C until full conversion of the starting material was detected by TLC (cyclohexane/EtOAc = 4:1) after 17 h. The mixture was filtrated through a pad of diatomaceous earth (2 cm, wetted with 1,4-dioxane) and the pad was rinsed with 1,4-dioxane (2 x 1 mL). The light-yellow filtrate was transferred into a 4 mL screw-cap vial with magnetic stirring bar, PhSH (13.0 µL, 1.3 eq) were added followed by 200 µL 2 M HCl in Et_2O at 22 °C. The mixture was stirred at 22 °C for 28 h, transferred into 2 mL Eppendorf vials, centrifuged and the supernatant was discarded. The colorless precipitate was suspended in Et_2O (2 mL), centrifuged and the obtained colorless solid was dried on air. The free-flowing, colorless solid was suspended in Et_2O (5 mL) and treated with saturated aqueous Na_2CO_3 (2.5 mL) followed by 25% aqueous NH_4OH (500 µL) in a separating funnel. The phases were mixed, separated and the aqueous layer was extracted with Et_2O (2 x 5 mL). The combined organic layers were washed with brine (3 mL), dried over Na_2SO_4, filtrated and the solvent was removed in vacuum. The product was dried in oil pump vacuum.[7]

Yield: 5.6 mg (23 µmol, 23%) yellow, very viscous liquid.

R_f = 0.14 (EtOAc/cyclohexane/Et_3N = 2:1:1% (v/v/v)) (254 nm, CAM: blue).

α_D^{20}: -16.1° (c = 0.28, acetone) (Lit. α_D^{20}= -49.3° (c = 1.0, acetone))[7]

^{1}H-NMR (300.13 MHz, $CDCl_3$): δ = 7.73 (bs, 1H), 7.49 (d, *J* = 7.3 Hz, 1H, 7.32 (dd, *J* = 7.1 Hz, *J* = 1.1 Hz, 1H), 7.19-7.05 (m, 2H), 4.12-4.01 (m, 1H), 3.45-3.31 (m, 1H), 3.10-2.97 (m, 1H), 2.79-2.71 (m, 2H), 1.95-1.80 (m, 1H), 1.77-1.19 (m, 7H), 0.99-0.85 (m, 3H). ^{13}C-NMR (75.47 MHz, $CDCl_3$): δ = 121.5, 119.4, 118.1, 110.7, 52.7, 42.7, 35.1, 32.1, 25.6, 22.8, 22.6, 14.1 (quarternary C-atoms were not detected due to low concentration). ^{1}H- and ^{13}C-NMR spectra were in accordance with reported data.[7]

(*R*)-*N*-*p*-Tolylsulfinyl tryptamine

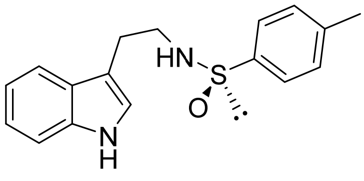

A oven dried 100 mL Schlenk tube with magnetic stirring bar was flushed with argon for 20 min. Tryptamine (327 mg, 2.00 mmol, 1 eq) was added followed by 20 mL dry THF and the mixture was cooled to -80 °C with a MeOH/liquid nitrogen cooling mixture. *n*-Butyl lithium solution in hexanes (1.93 mL, 4.10 mmol, 2.05 eq) was added dropwise over a period of 15 min. The cooling bath was removed and the mixture was allowed to warm to ambient temperature. TMSCl (272 µL, 233 mg, 2.10 mmol, 1.05 eq) was added, the mixture was left stirring at 22 °C for 30 min followed by dropwise addition of *n*-butyl lithium

(986 µL, 2.10 mmol, 1.05 eq). The yellow solution was stirred at 22 °C for 1 h upon which precipitation occurred. The turbid mixture was added dropwise to a vigorously stirred solution of (1*R*,2*S*,5*R*)-(*S*)-menthyl *p*-toluenesulfinate (601 mg, 2.00 mmol, 1 eq) in dry THF (15 mL) at 22 °C over a period of 15 min. The dirty-yellow solution was stirred for 1 h at 22 °C, quenched by the addition of 0.1 M aqueous Na_2HPO_4 solution (20 mL). The mixture was transferred into a separating funnel, the phases were separated and the aqueous layer was extracted with EtOAc (4 x 50 mL). The combined organic layers were dried over Na_2SO_4, filtrated and the solvent was removed in vacuum. The crude product was adsorbed on diatomaceous earth (EtOAc, MeOH) and was purified by column chromatography (31 x 2 cm, cyclohexane/EtOAc = 1:1 (v/v)).[8]

Yield: 496 mg (1.66 mmol, 83%) light-yellow solid; mp: 139-141 °C (Lit.[8] 109 °C);
R_f = 0.21 (cyclohexane/EtOAc = 1:1 (v/v)) (254 nm, CAM: black); α_D^{20}: +60.9° (c = 1.00, acetone) (lit. α_D^{20} = +102°).[8]

(*S*)-1-Pentyl-2,3,4,9-tetrahydro-1*H*-pyrido[3,4-*b*]indole ((*S*)-3d)

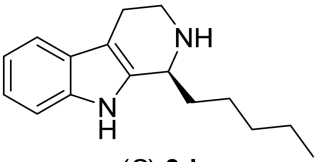

(*S*)-**3d**

Step 1: A dry 50 mL Schlenk tube with magnetic stirring bar was flushed with argon for 15 min. (*R*)-*N*-*p*-Tolylsulfinyl tryptamine (250 mg, 838 µmol, 1 eq) was added followed by $CH_2Cl_2/CHCl_3$ = 1:1 (8.4 mL). The mixture was cooled to -90 °C with an acetone/dry ice cooling bath and hexanal (103 µL, 83.9 mg, 838 mmol, 1 eq) were added followed by CSA (39.7 mg, 168 µmol, 0.2 eq). The mixture was left stirring in the cooling bath without further addition of dry ice for 7 h (temperature of 10 °C reached). The reaction was quenched with Et_3N (7 mL), the solvents were removed in vacuum and the product was purified by column chromatography (30 x 2 cm, cyclohexane/EtOAc = 12:1, 9:1, 7:1 (v/v)). The product (41 mg) was not analytically pure and was used in the subsequent step without further purification.

Step 2: The Pictet-Spengler product from the previous step was dissolved in EtOH (3 mL) and transferred into a 25 mL round-bottom flask with magnetic stirring bar. The mixture was cooled to 0 °C and concentrated aqueous HCl (37%, 200 µL) was added in one portion. The faint-yellow solution was stirred for 5 min at 0 °C and was quenched with saturated aqueous K_2CO_3 solution (3 mL). EtOAc (5 mL) was added, the mixture was stirred for 20 min, the phases were separated and the aqueous layer was extracted with EtOAc (3 x 5 mL). The combined organic layers were dried over Na_2SO_4, filtrated and the solvent was removed in vacuum. The crude

product was adsorbed on silica gel (MeOH) and purified by flash column chromatography (size: 16 x 0.8 cm, EtOAc/cyclohexane/Et_3N = 2:1:1% (v/v/v)).[7]

Yield: 14.9 mg (61.5 µmol, 7%) light-yellow, very viscous liquid.

R_f = 0.14 (EtOAc/cyclohexane/Et_3N = 2:1:1% (v/v/v)) (254 nm, CAM: blue).

α_D^{20}: -18.2° (c = 0.74, acetone) (Lit.[7] α_D^{20}= -49.3° (c = 1.0, acetone)).

^{1}H-NMR (300.13 MHz, $CDCl_3$): δ = 7.81 (bs, 1H), 7.49 (d, *J* = 7.3 Hz, 1H), 7.35–7.29 (m, 1H), 7.20–7.06 (m, 2H), 4.11–4.01 (m, 1H), 3.43–3.31 (m, 1H), 3.09–2.97 (m, 1H), 2.79–2.71 (m, 2H), 1.94–1.80 (m, 1H), 1.75–1.27 (m, 7H), 0.95–0.87 (m, 3H). ^{13}C-NMR (75.47 MHz, $CDCl_3$): δ = 124.5, 119.4, 118.1, 110.7, 52.7, 42.7, 35.1, 32.1, 25.6, 22.7, 22.6, 14.1 (quarternary C-atoms were not detected due to low concentration). ^{1}H- and ^{13}C-NMR spectra were in accordance with reported data.[7]

Analytics

Determination of the conversion of natural substrates

The conversions were measured by HPLC using a Shimadzu system equipped with an UV/VIS detector using a Phenomenex LUNA C18(2) column (0.64 cm x 25 cm, 5 µm). Method parameters are given in Table S9. Retention times: tryptamine (**1**) = 7.3 min, secologanin = 7.7 min, (*S*)-strictosidine = 8.9 min (Figure S9).

Table S9. HPLC method characteristics and parameters for determination of conversion.

Method parameters	
Flow rate	1.0 mL / min
Detection	UV at 254 nm and 280 nm
Time	15 minutes
Oven temperature	25 °C
Injection volume	50 µL
Solvent system	MeCN (containing 0.1% TFA)/NH_4COOH (30 mM, pH 2.8)
Gradient	10:90 -> 50:50 within 8 min
	50:50 -> 80:20 within 3 min
	80:20 -> 10:90 within 0.5 min
	10:90 for 3.5 min

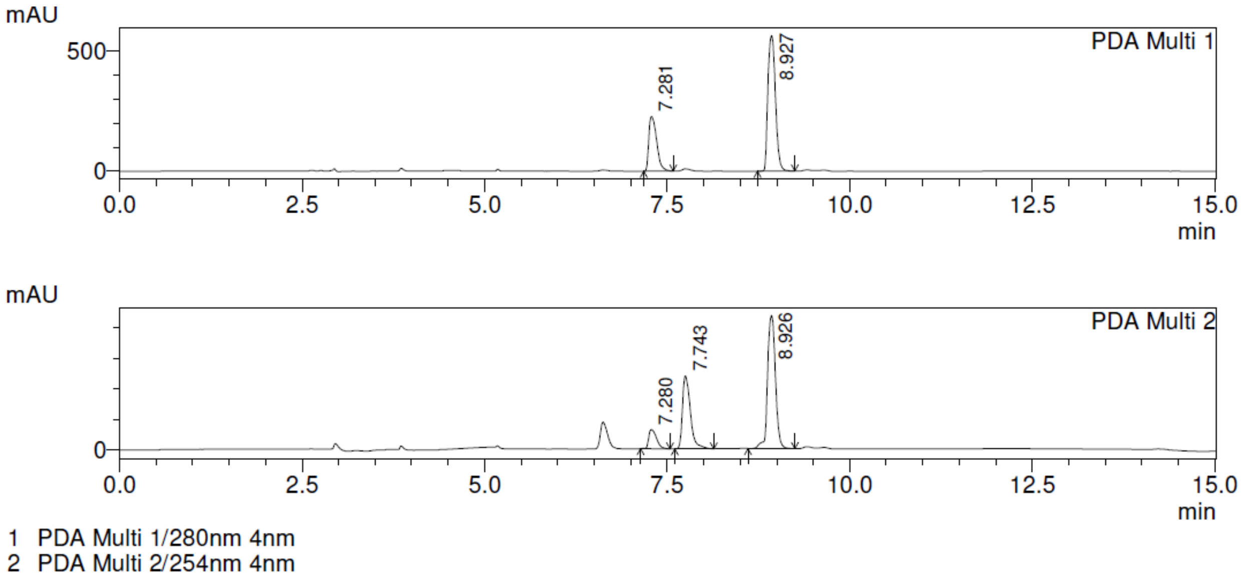

Figure S9. HPLC chromatograms at 280 nm (top) and 254 nm (bottom).

Determination of the conversion of non-natural aldehydes

The conversions were measured by GC using an Agilent 7890 A GC system equipped with an FID detector using an Agilent J&W HP-5 column (30 m, 320 µm and 0.25 µm). Helium was used as carrier gas and EtOAc was used to dissolve the sample.

Method for GC-FID: injector 300 °C, flow 36.388 cm/sec, 2 mL/min, split ratio 15:1. Temperature program: 100 °C/hold 0.5 min, 300 °C/rate 10 °C per min/hold 0 min. Injection volume: 1 µL.

Retention times, response values are given in brackets: tryptamine (**1**) = 9.9 min, THBC **3a** = 13.7 min (163.9), THBC **3b** = 13.2 min (105.7), THBC **3c** = 11.7 min (86.2), THBC **3d** = 15.1 min (137.9), lactam **4** = 16.4 min (108.4), harmicine (**5**) = 17.2 min (no response value determined),

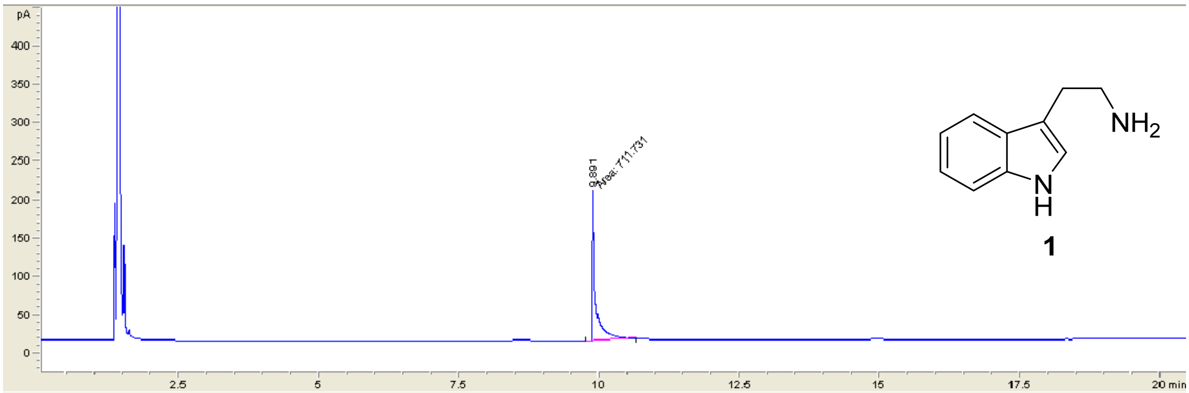

Figure S10. GC-FID chromatogram of tryptamine (**1**).

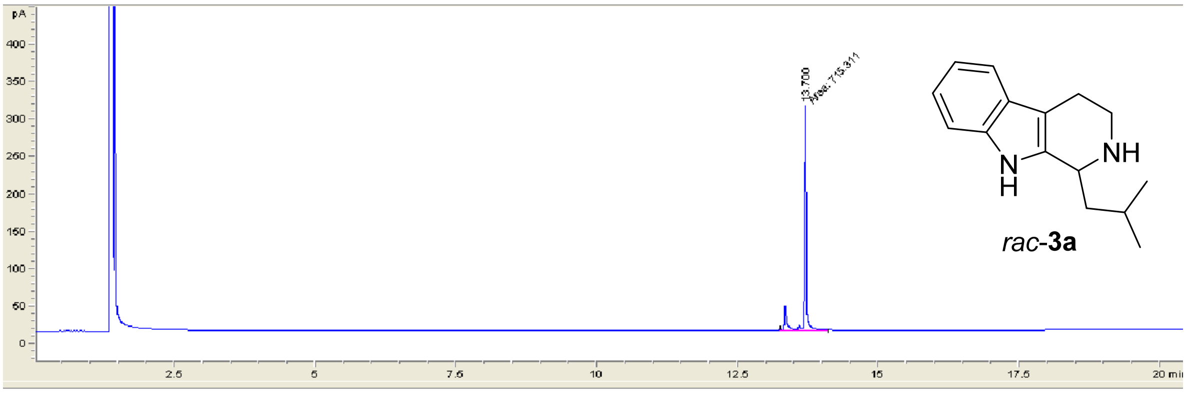

Figure S11. GC-FID chromatogram of the THBC **3a**.

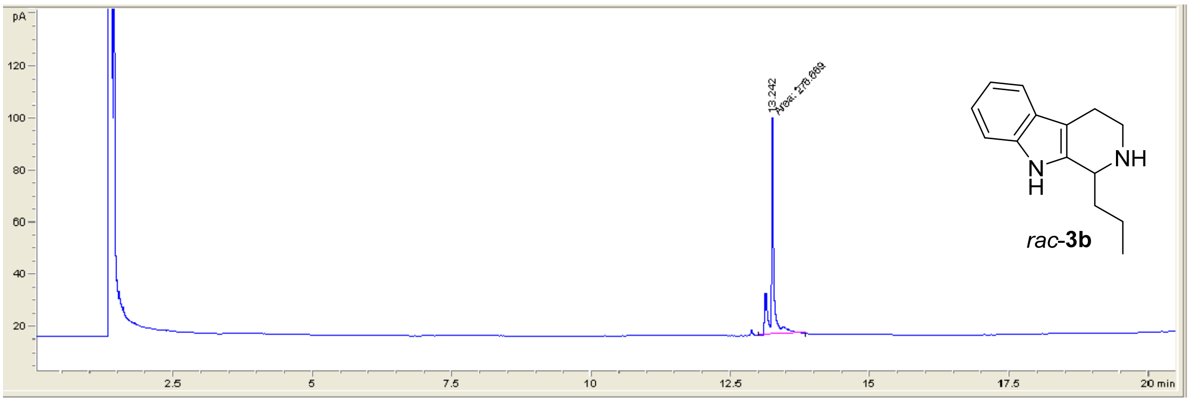

Figure S12. GC-FID chromatogram of the THBC **3b**.

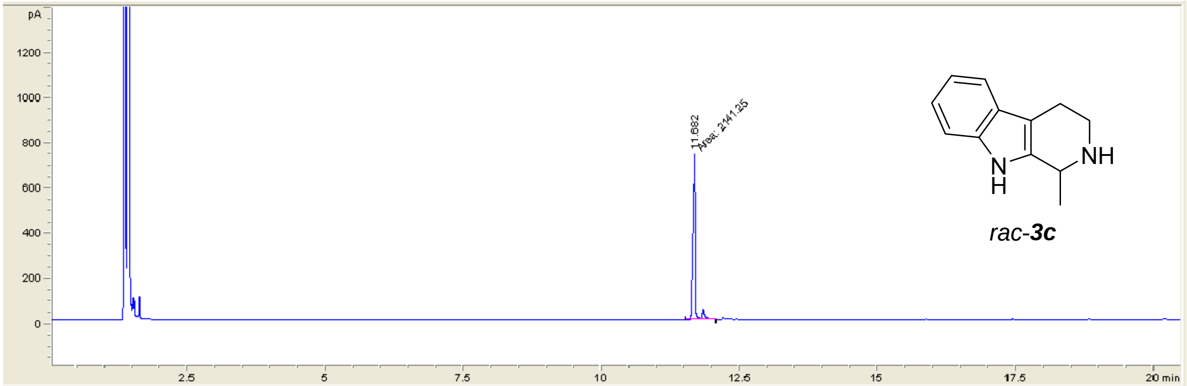

Figure S13. GC-FID chromatogram of the THBC **3c**.

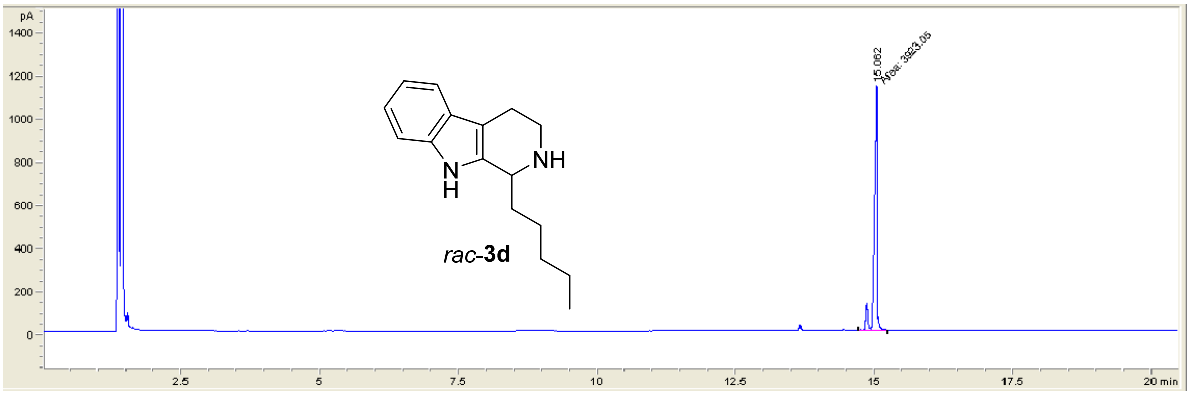

Figure S14. GC-FID chromatogram of the THBC **3d**.

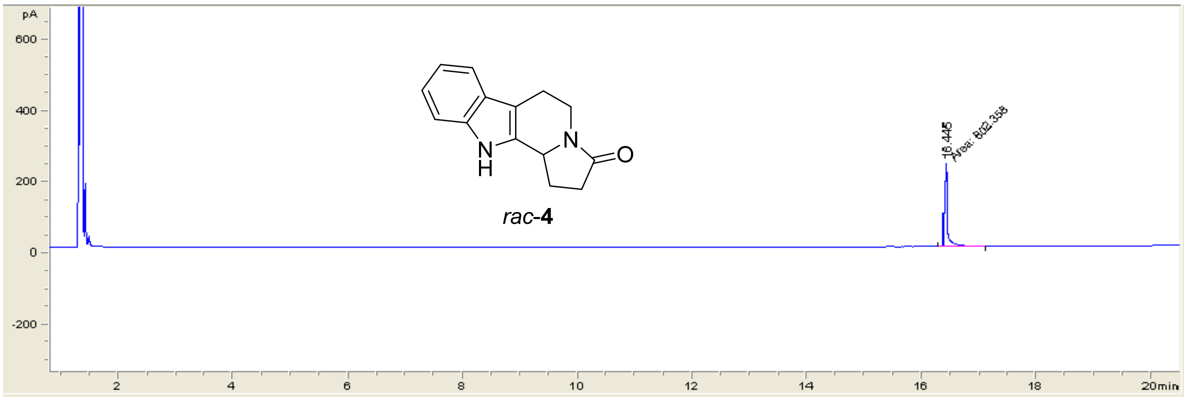

Figure S15. GC-FID chromatogram of the lactam **4**.

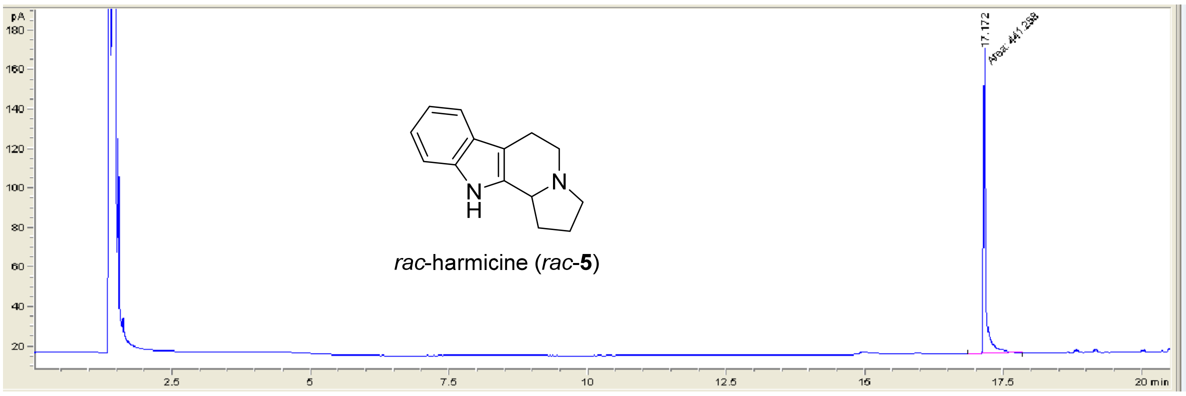

Figure S16. GC-FID chromatogram of harmicine (**5**).

Determination of *enantiomeric excess* for transformations with non-natural aldehydes

The samples were dissolved in EtOAc after determination of the conversion and purified *via* preparative TLC (silica gel plates, eluent: CH_2Cl_2:MeOH:TEA = 90:9:1). The product was purified *via* TLC and extracted from the solid phase (100 µL 2-PrOH, 400 µL *n*-Heptane + 0.1% *N*,*N*-diethylamine DEA). The samples were centrifuged and the *ee* of the supernatant was measured by HPLC on a chiral phase and *via* an UV/VIS detector. The product was eluted with an isocratic flow of *n*-heptane and 2-PrOH containing 0.1% DEA. The column, conditions and retention times for the respective separations are given in Table S10.

Table S10. HPLC parameters for determination of *ee*s.

THBC	Column	Eluent[a]	Flow [mL/min]	Oven [°C]	Retention times [min]	
					(*S*)	(*R*)
**3a**	Chiralpak IC	90:10	1.0	25	5.8	7.5
**3b**	Chiralpak IC	90:10	1.0	25	6.9	9.4
**3c**	Chiralpak IC	90:10	1.0	25	8.8	9.7
**3d**	Chiralpak IC	90:10	1.0	25	6.5	9.1
**4**	Chiralcel OD-H	90:10	1.0	40	21.3	18.5
**5**	Chiralpak IC	90:10	1.0	25	6.9	9.5

[a] *n*-Heptane containing 0.1% DEA and 2-PrOH cotaining 0.1% DEA.

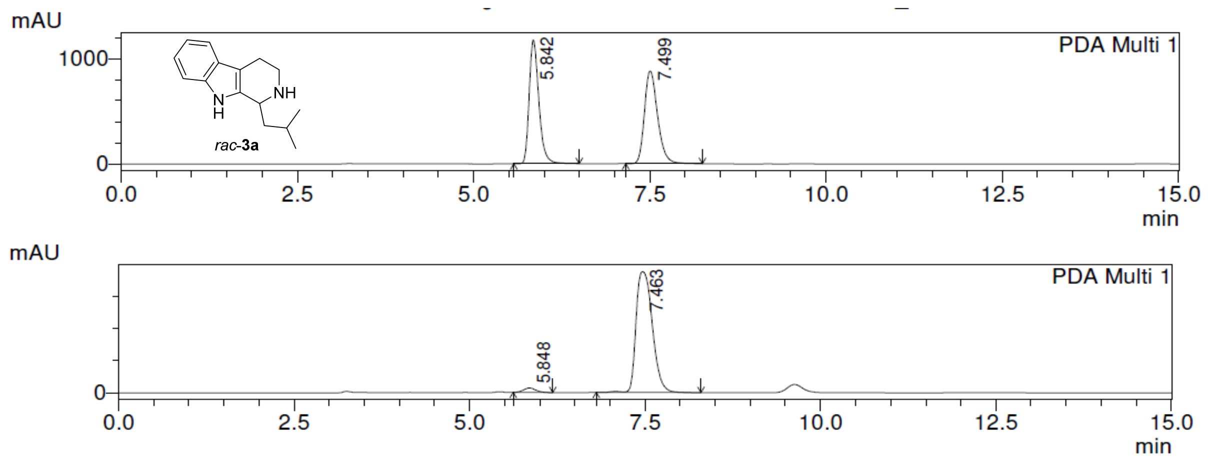

Figure S17. HPLC chromatograms of the racemic reference material *rac*-**3a** (top) and the product of the biotransformation with His_6-RsSTR (bottom) **3a**.

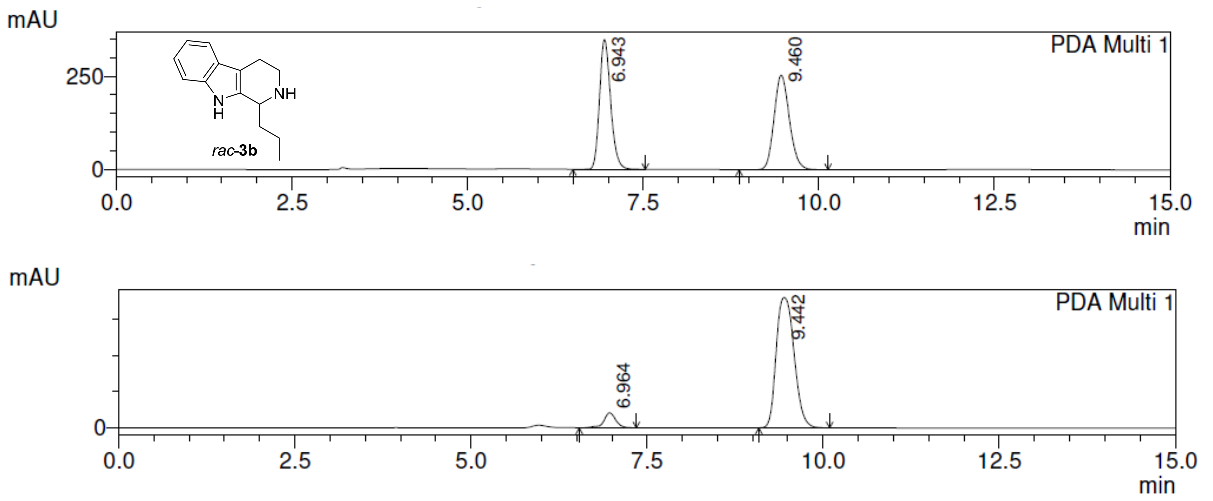

Figure S18. HPLC chromatograms of racemic reference material *rac*-**3b** (top) and the product **3b** of the biotransformation with His_6-OpSTR (bottom).

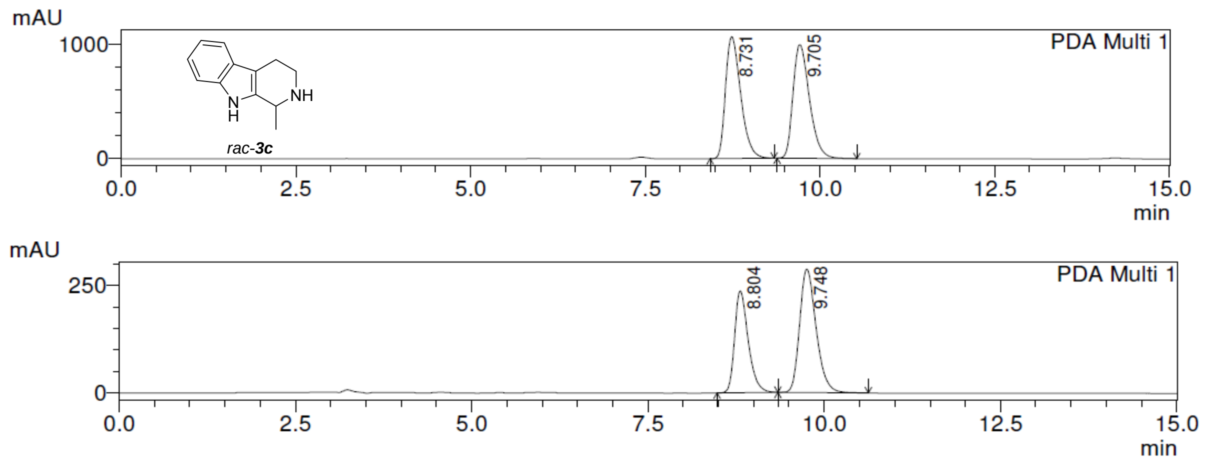

Figure S19. HPLC chromatograms of racemic reference material *rac*-**3c** (top) and the product **3c** of the biotransformation with His_6-RsSTR (bottom).

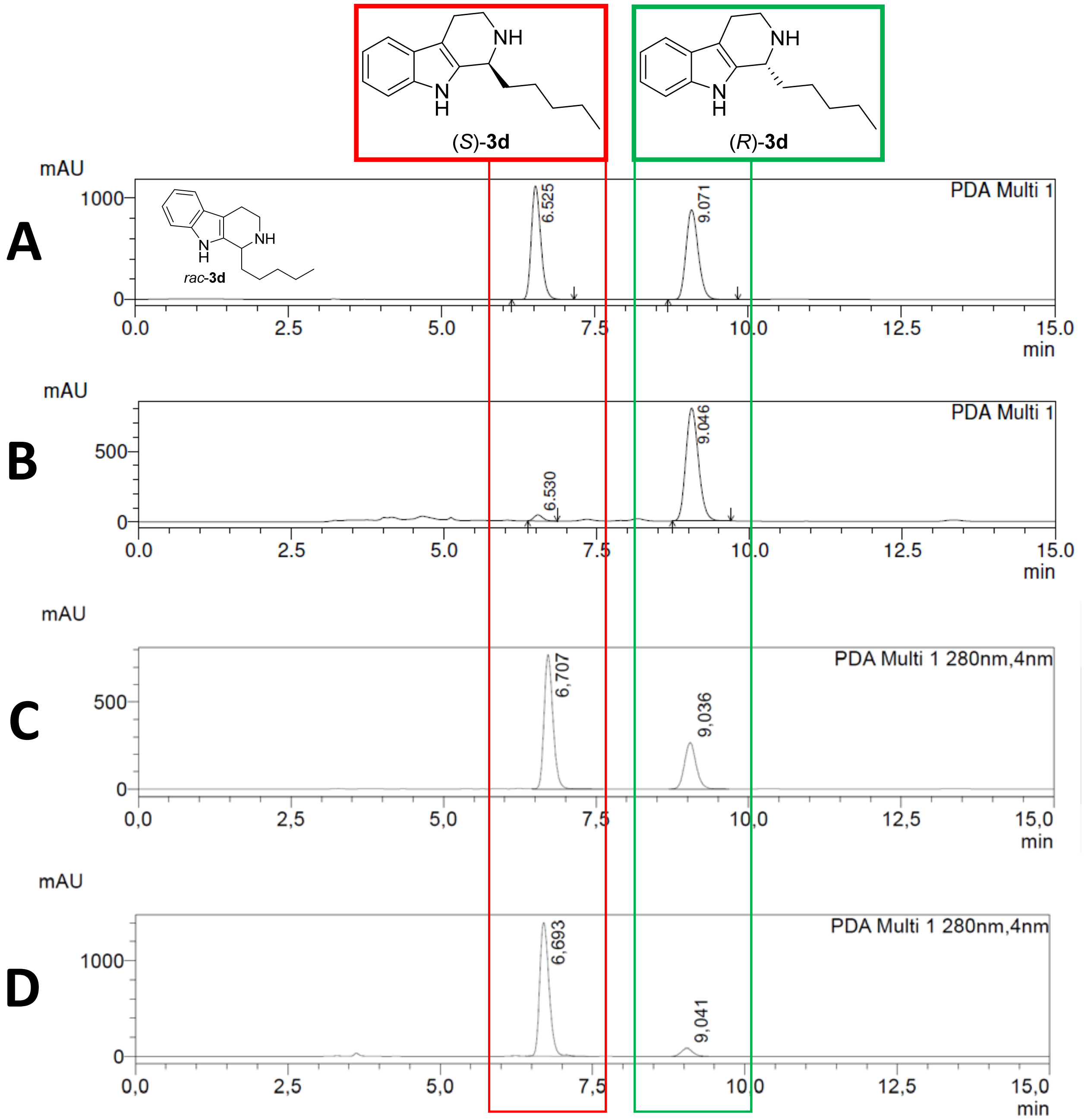

Figure S20. HPLC chromatograms of a) racemic reference material *rac*-**3d** (A), product of the enzymatic reaction with RvSTR and aldehyde **2d** (82% ee) (B), enantioenriched reference material (*S*)-**3d** (38% ee) synthesized via Pictet-Spengler reaction using an *N*-sulfinyl chiral auxiliary according to Gremmen (C),[8] enantioenriched reference material (*S*)-**3d** (86% ee) synthesized via Pictet-Spengler reaction using a chiral Brønsted acid according to Wanner (D).[7]

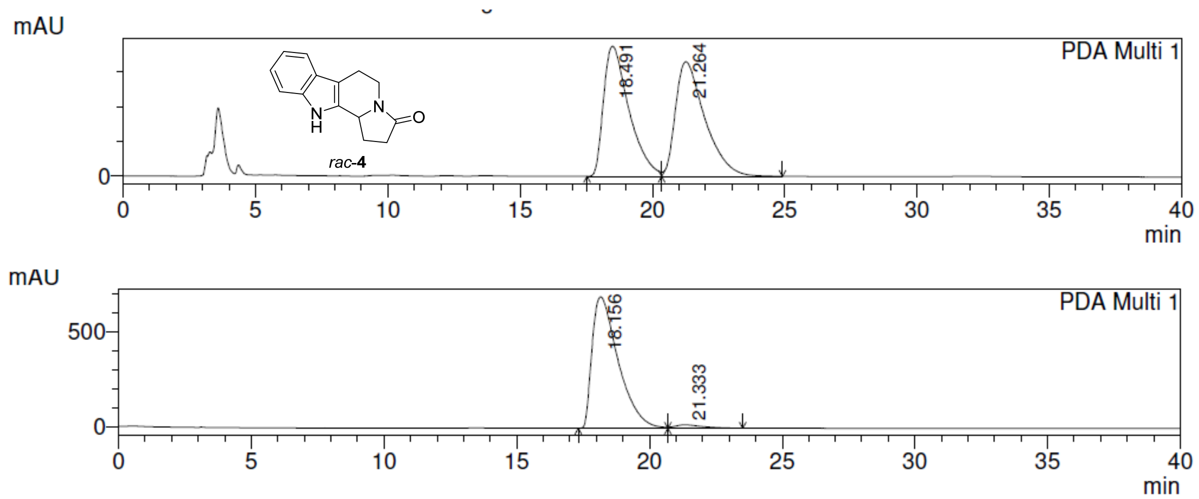

Figure S21. HPLC chromatograms of racemic reference material *rac*-**4** (top) and the product **4** of the biotransformation with His_6-RsSTR (bottom).

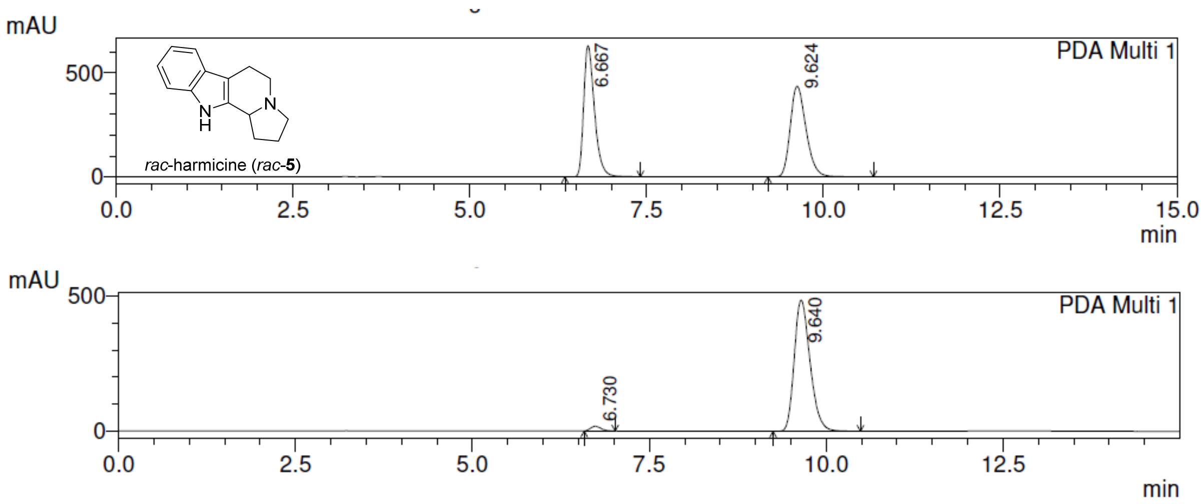

Figure S22. HPLC chromatograms of racemic reference material *rac*-**5** (top) and enantioenriched (*R*)-harmicine (*R*)-**5** (bottom).

Spectra

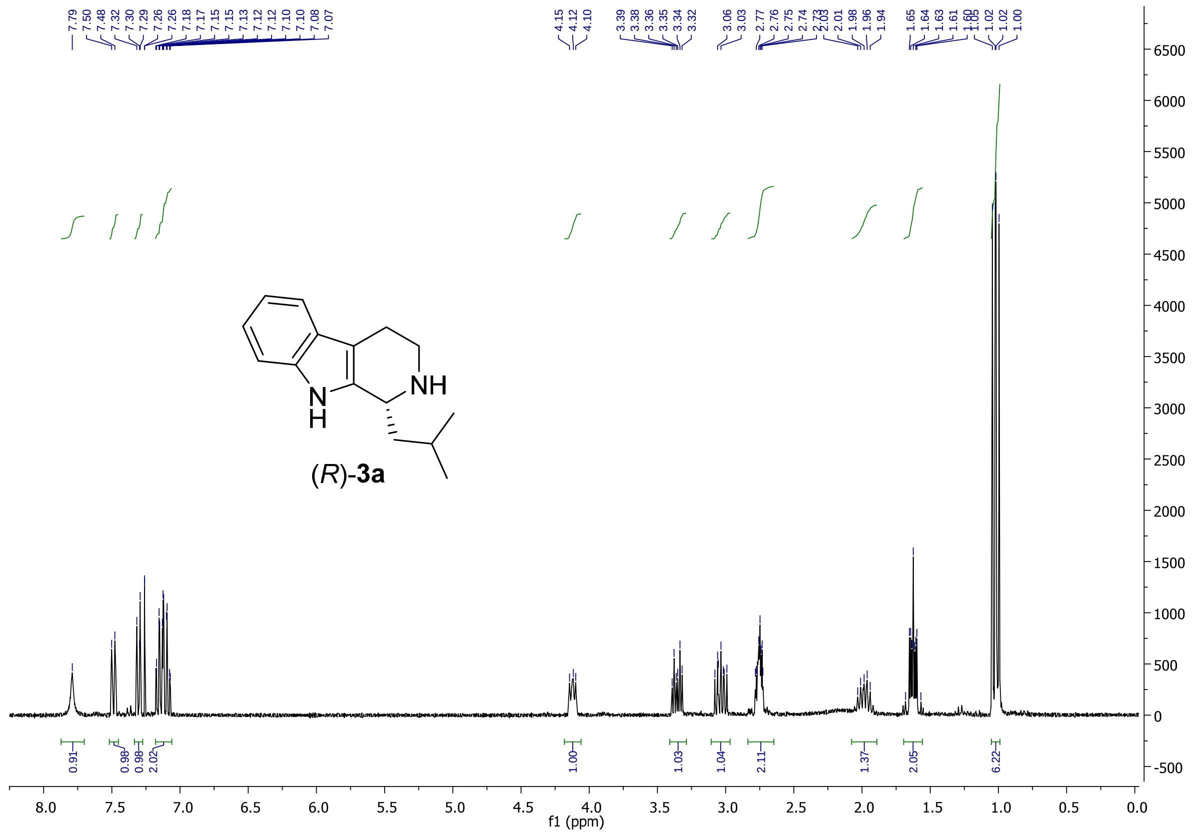

Figure S23. ^{1}H-NMR spectrum of (*R*)-1-isobutyl-1,2,3,4-tetrahydro-9*H*-pyrido[3,4-*b*]indole (*R*)-**3a**.

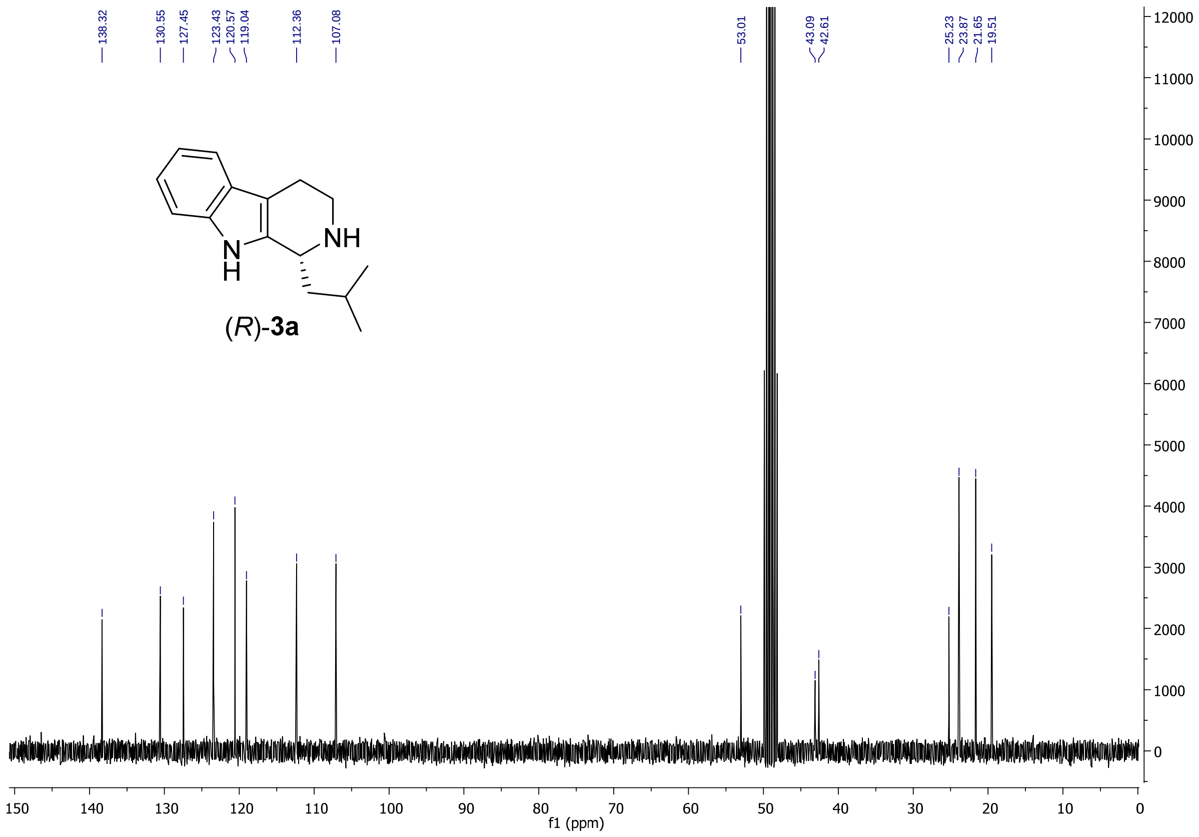

Figure S24. ^{13}C-NMR spectrum of (*R*)-1-isobutyl-1,2,3,4-tetrahydro-9*H*-pyrido[3,4-*b*]indole (*R*)-**3a**.

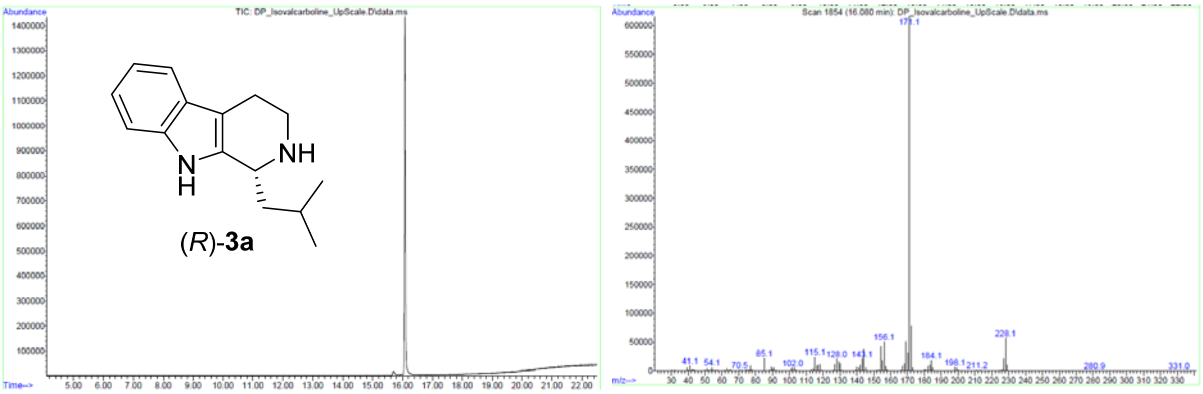

Figure S25. GC-MS spectrum and fragmentation pattern of (*R*)-1-isobutyl-1,2,3,4-tetrahydro-9*H*-pyrido[3,4-*b*]indole (*R*)-**3a**.

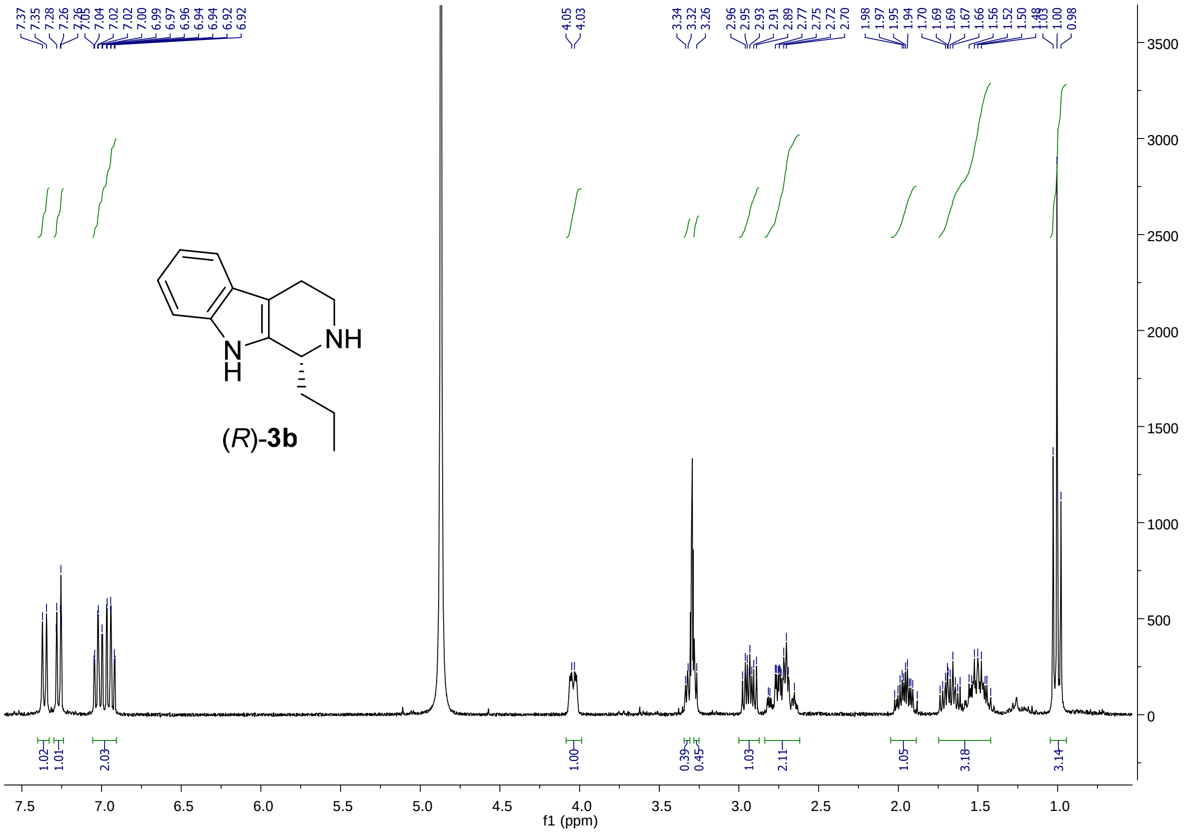

Figure S26. ^{1}H-NMR spectrum of (*R*)-1-propyl-2,3,4,9-tetrahydro-1*H*-pyrido[3,4-*b*]indole (*R*)-**3b**.

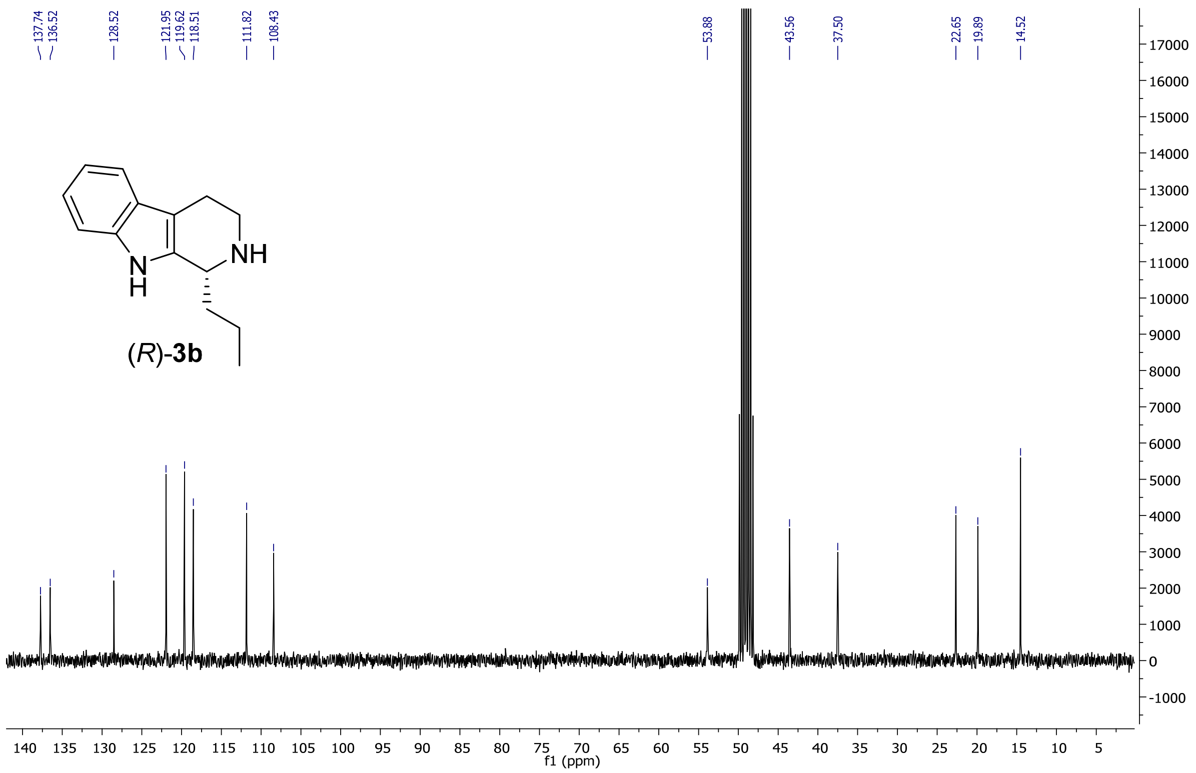

Figure S27. ^{13}C-NMR spectrum of (*R*)-1-propyl-2,3,4,9-tetrahydro-1*H*-pyrido[3,4-*b*]indole (*R*)-**3b**.

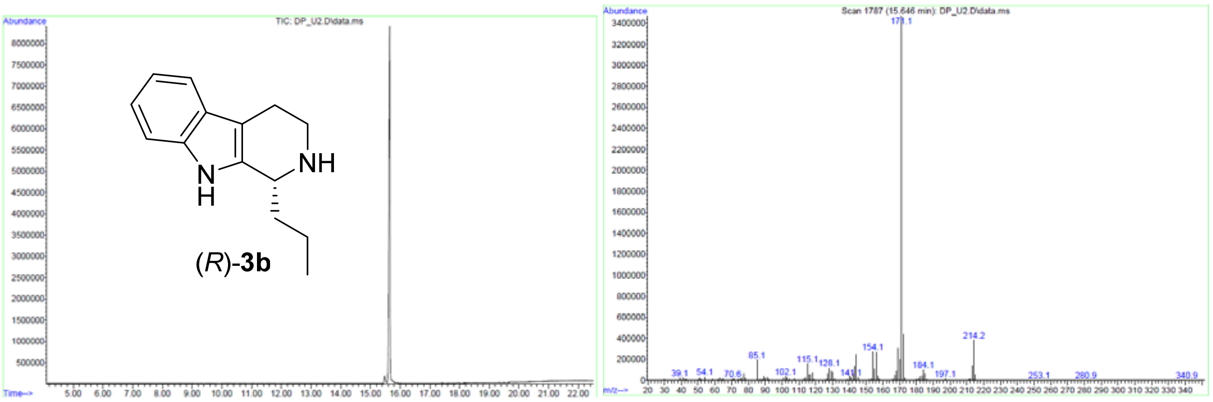

Figure S28. GC-MS spectrum and fragmentation pattern of (*R*)-1-propyl-2,3,4,9-tetrahydro-1*H*-pyrido[3,4-*b*]indole (*R*)-**3b**.

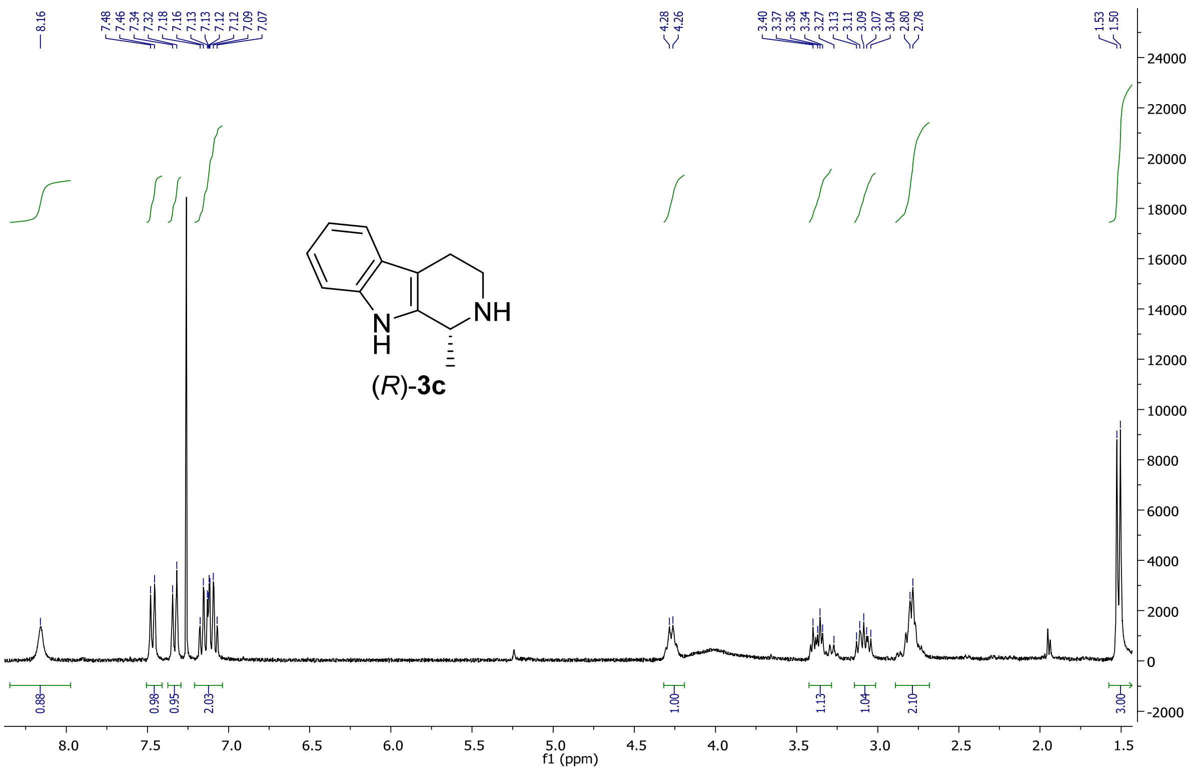

Figure S29. ^{1}H-NMR spectrum of (*R*)-1-methyl-2,3,4,9-tetrahydro-1*H*-pyrido[3,4-*b*]indole (*R*)-**3c**.

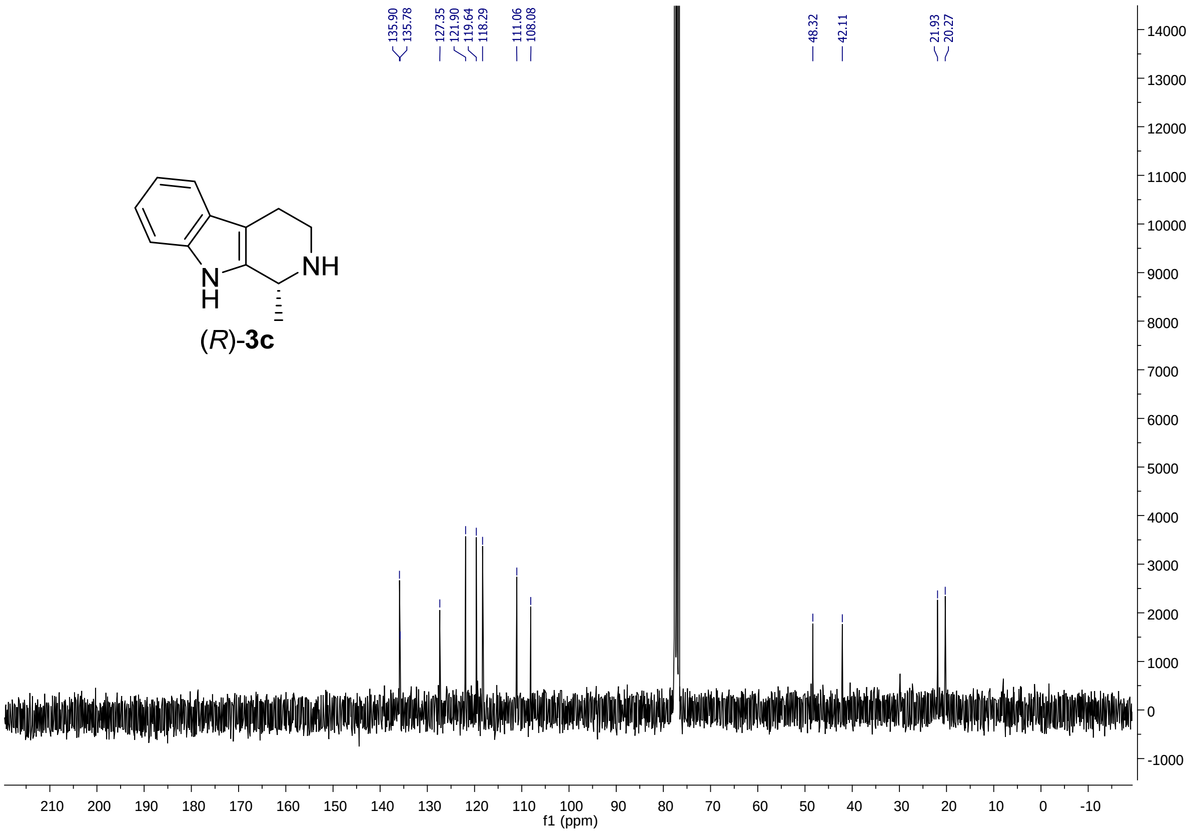

Figure S30. ^{13}C-NMR spectrum of (*R*)-1-methyl-2,3,4,9-tetrahydro-1*H*-pyrido[3,4-*b*]indole (*R*)-**3c**.

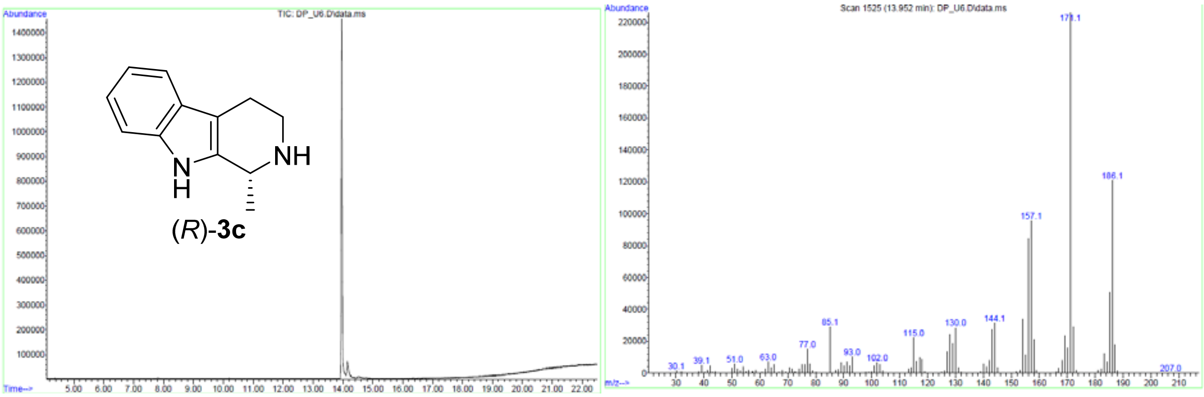

Figure S31. GC-MS spectrum and fragmentation pattern of (*R*)-1-methyl-2,3,4,9-tetrahydro-1*H*-pyrido[3,4-*b*]indole (*R*)-**3c**.

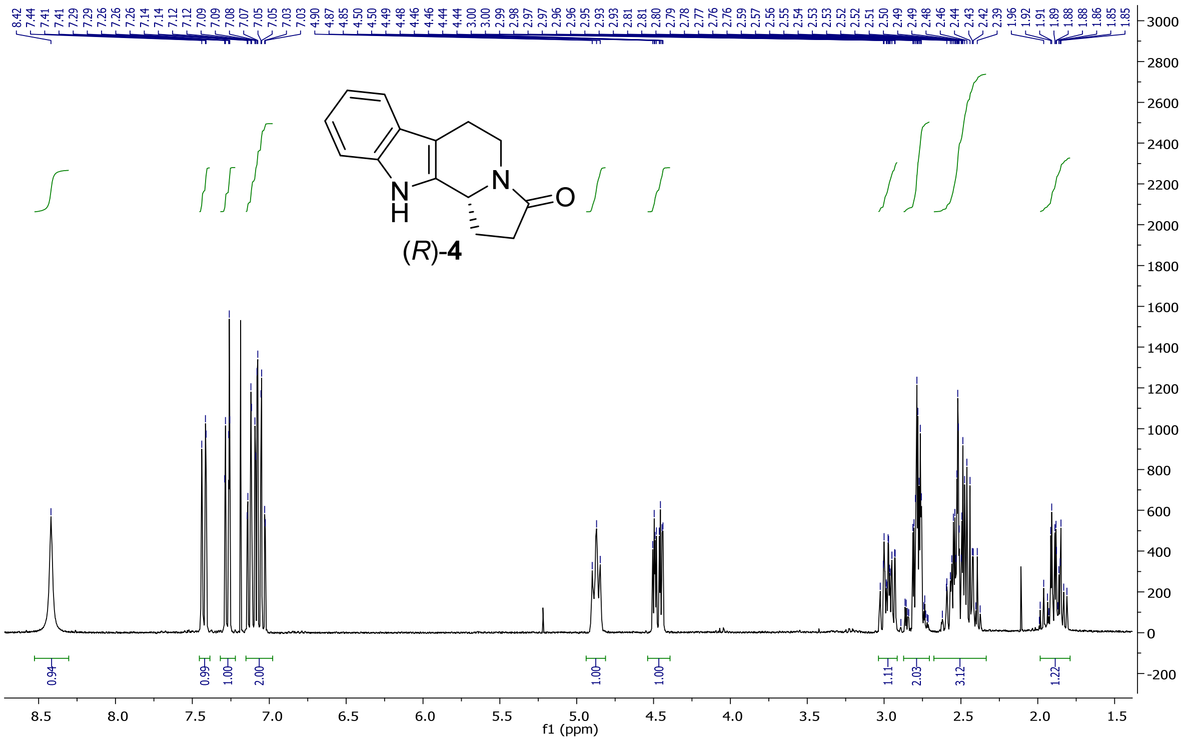

Figure S32. ^{1}H-NMR spectrum of (*R*)-1,2,5,6,11,11b-hexahydro-3*H*-indolizino[8,7-*b*]indol-3-one (*R*)-**4**.

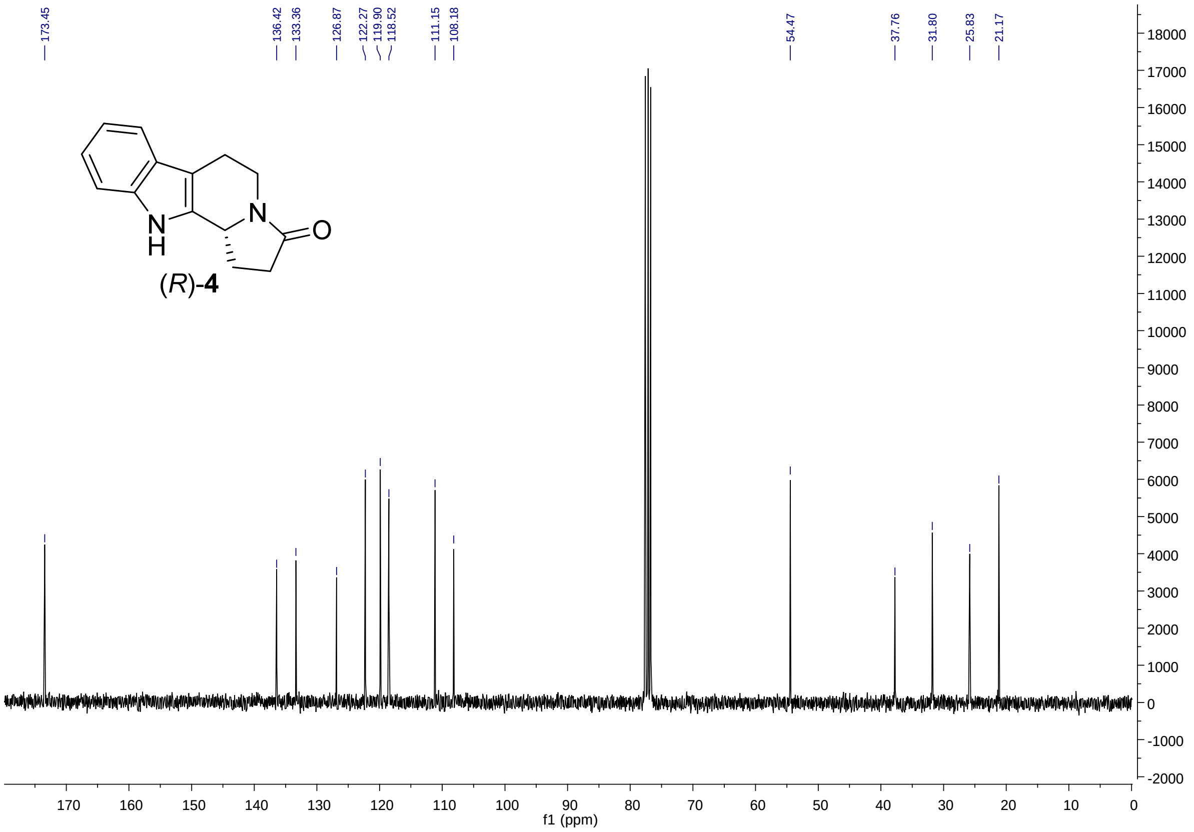

Figure S33. ^{13}C-NMR spectrum of (*R*)-1,2,5,6,11,11b-hexahydro-3*H*-indolizino[8,7-*b*]indol-3-one (*R*)-**4**.

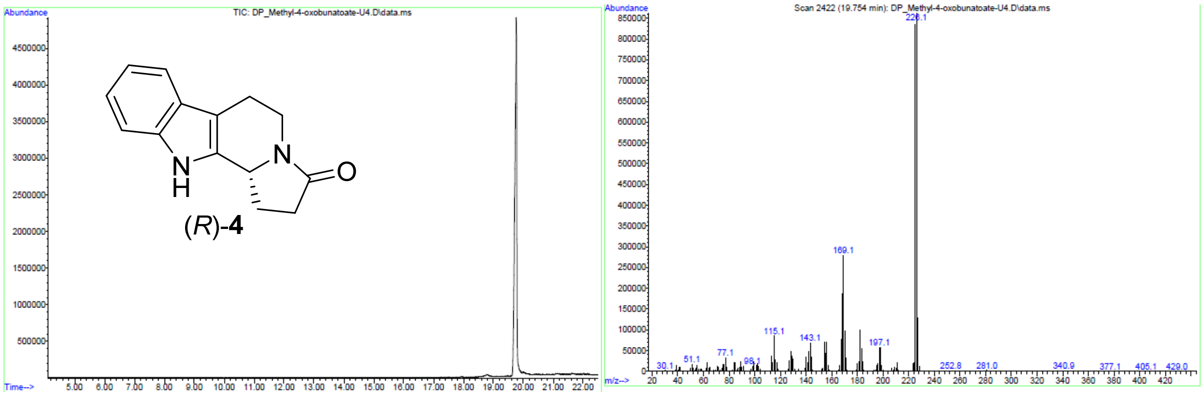

Figure S34. GC-MS spectrum and fragmentation pattern of (*R*)-1,2,5,6,11,11b-hexahydro-3*H*-indolizino[8,7-*b*]indol-3-one (*R*)-**4**.

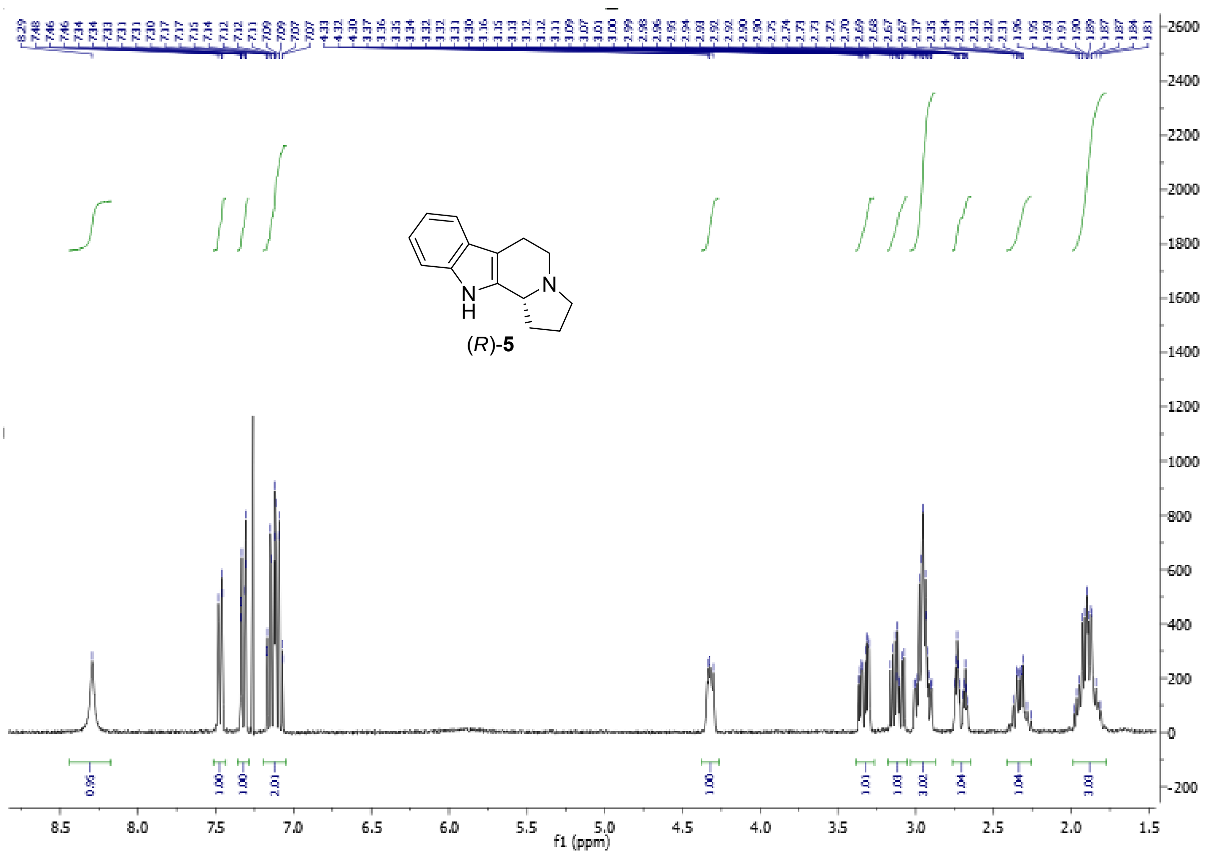

Figure S35. ^{1}H-NMR spectrum of (*R*)-harmicine (*R*)-**5**.

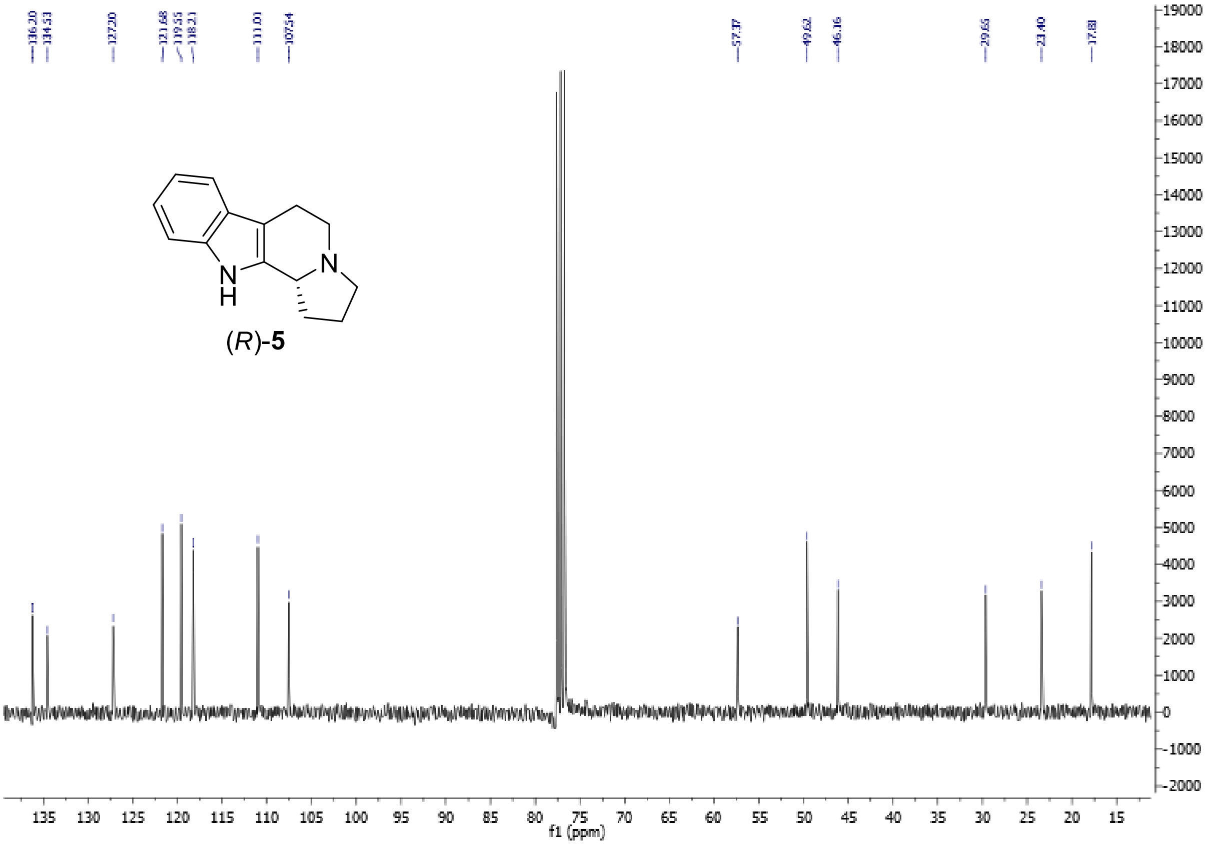

Figure S36. ^{13}C-NMR spectrum of (*R*)-harmicine (*R*)-**5**.

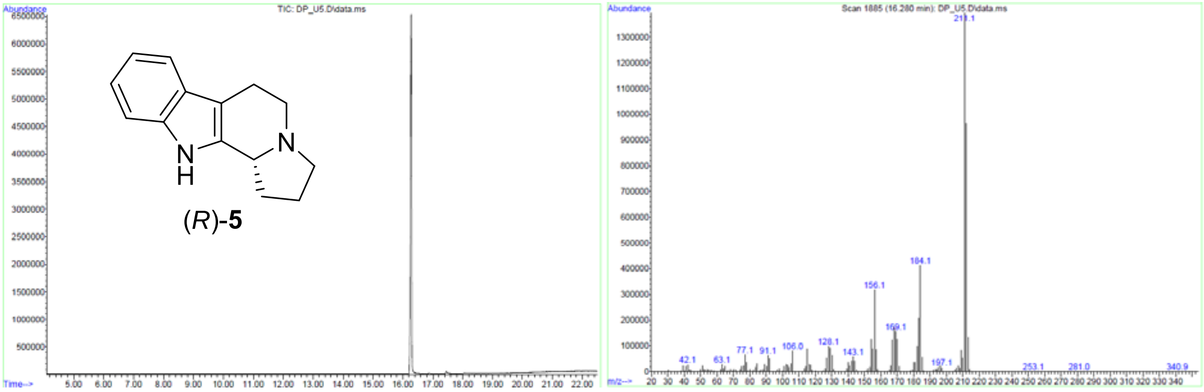

Figure S37. GC-MS spectrum and fragmentation pattern of (*R*)-harmicine (*R*)-**5**.

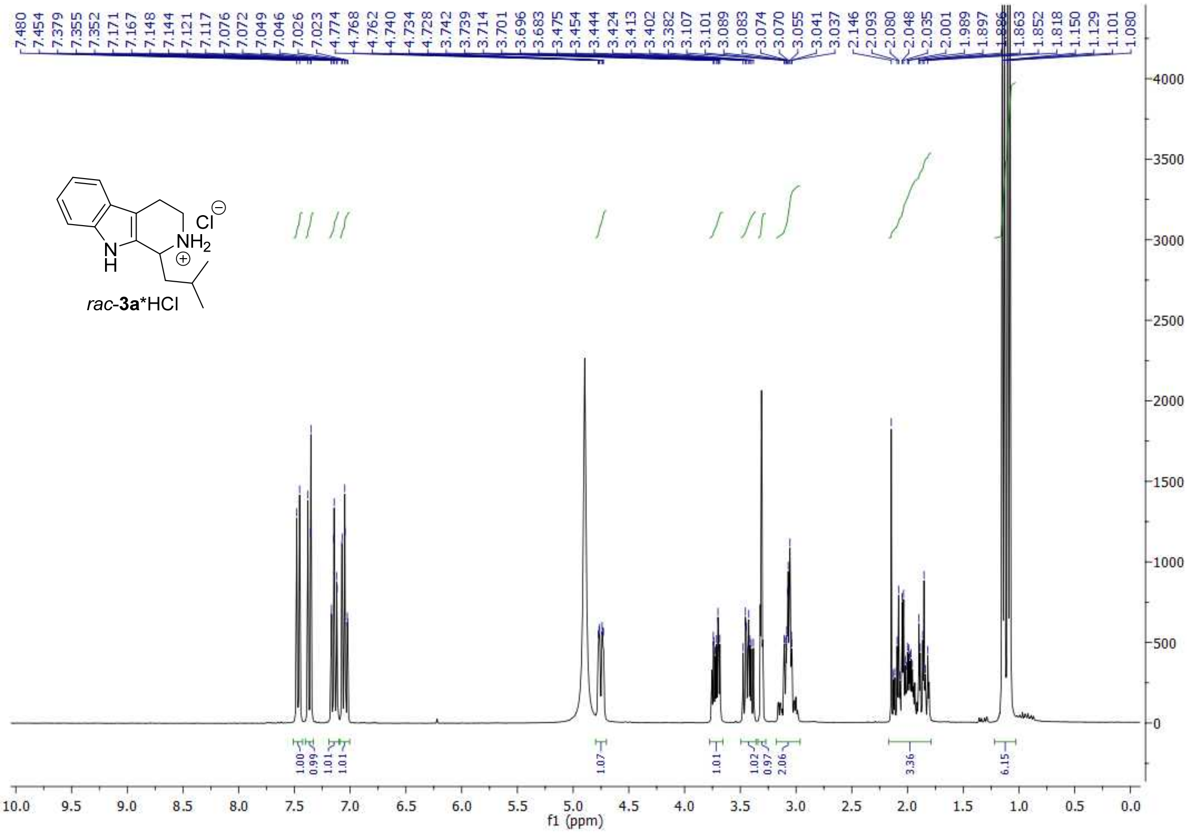

Figure S38. ^{1}H-NMR spectrum of *rac*-1-isobutyl-2,3,4,9-tetrahydro-1*H*-pyrido[3,4-*b*]indol-2-ium chloride (*rac*-**3a***HCl).

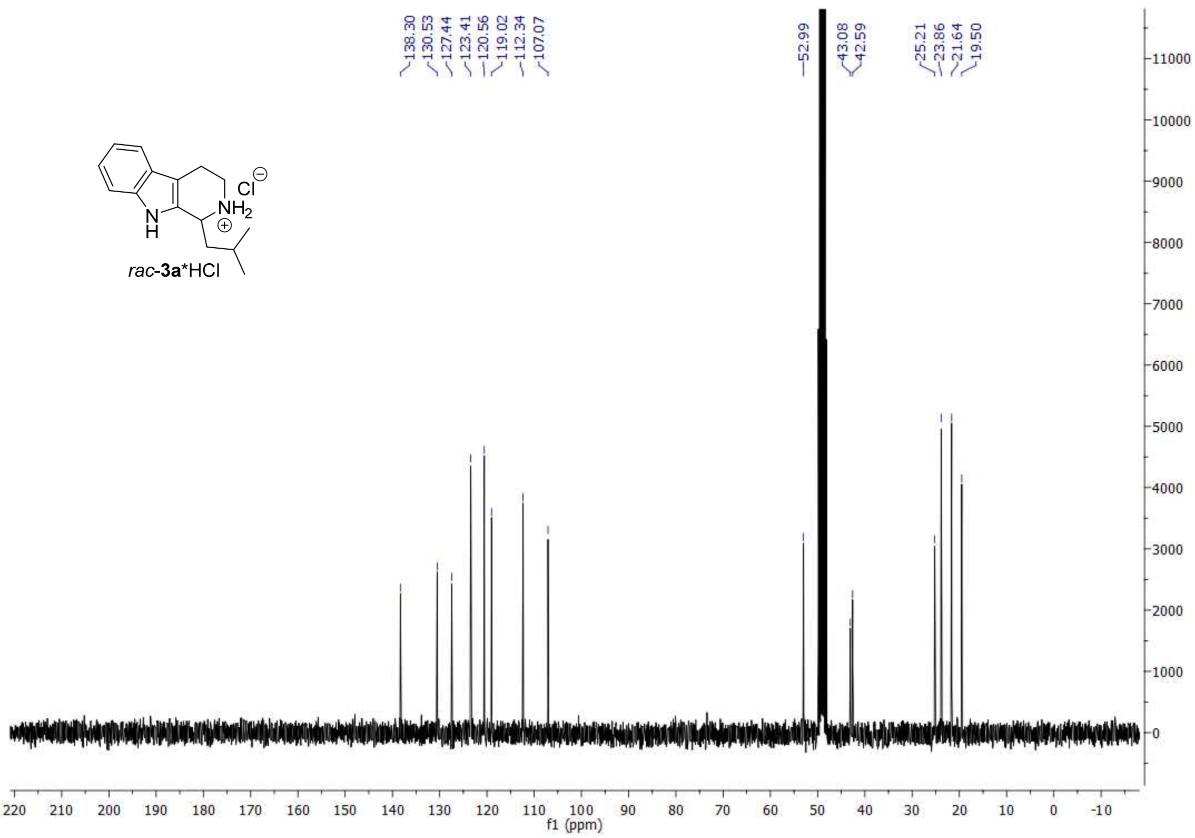

Figure S39. ^{13}C-NMR spectrum of *rac*-1-isobutyl-2,3,4,9-tetrahydro-1*H*-pyrido[3,4-*b*]indol-2-ium chloride (*rac*-**3a***HCl).

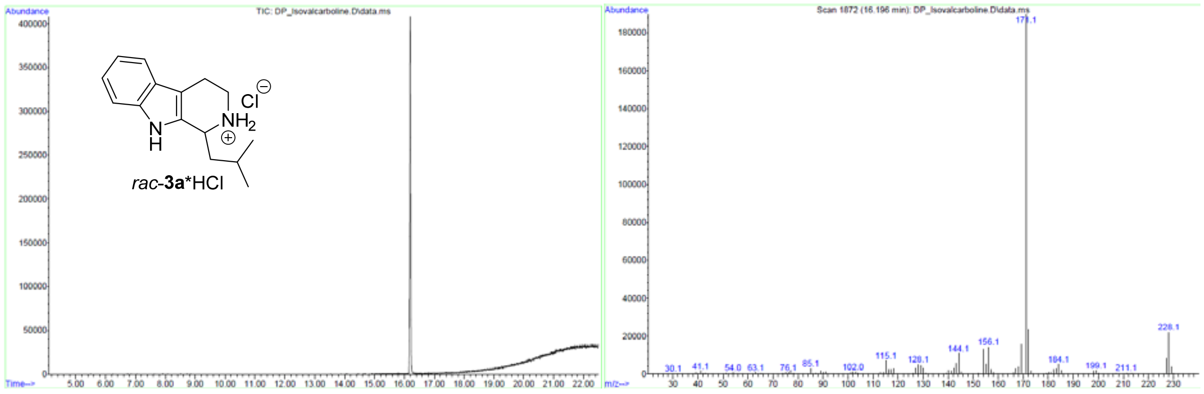

Figure S40. GC-MS spectrum and fragmentation pattern of *rac*-1-isobutyl-2,3,4,9-tetrahydro-1*H*-pyrido[3,4-*b*]indol-2-ium chloride (*rac*-**3a***HCl)

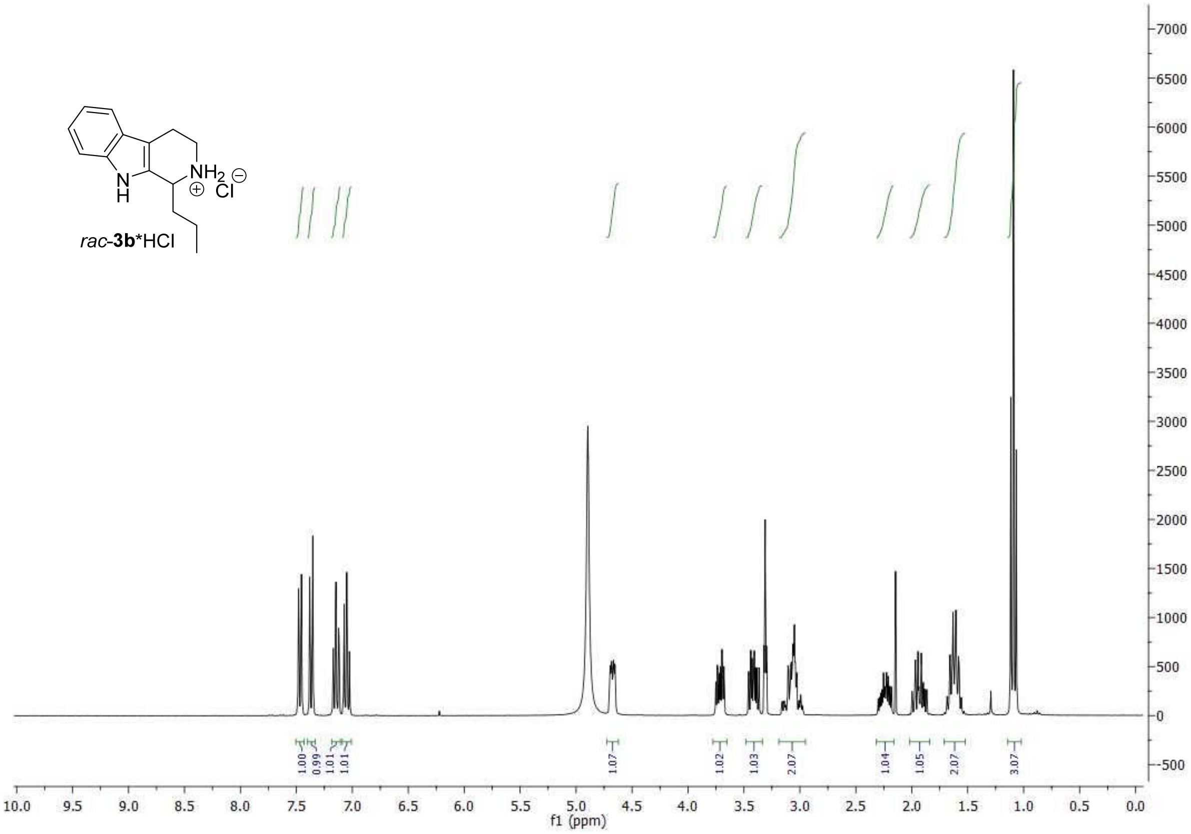

Figure S41. ^{1}H-NMR spectrum of 1-propyl-2,3,4,9-tetrahydro-1*H*-pyrido[3,4-*b*]indol-2-ium chloride (*rac*-**3b***HCl).

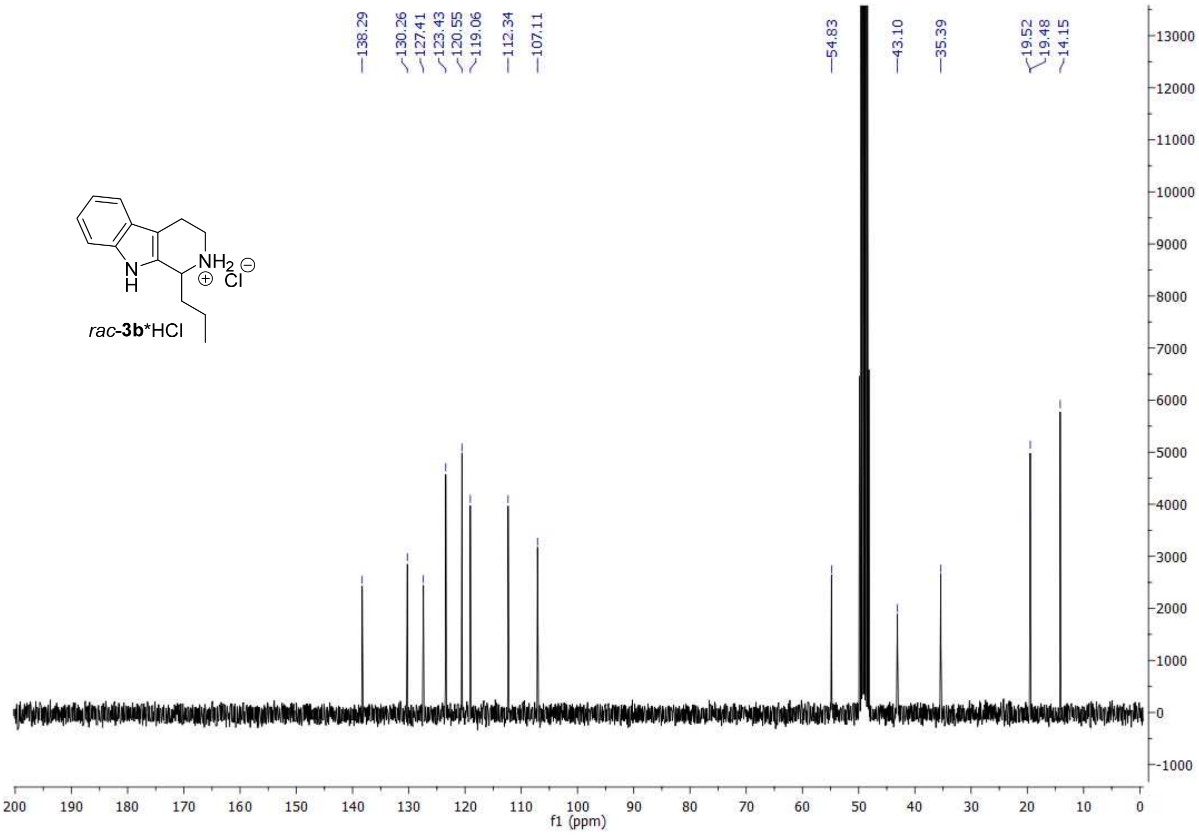

Figure S42. ^{13}C-NMR spectrum of 1-propyl-2,3,4,9-tetrahydro-1*H*-pyrido[3,4-*b*]indol-2-ium chloride (*rac*-**3b***HCl).

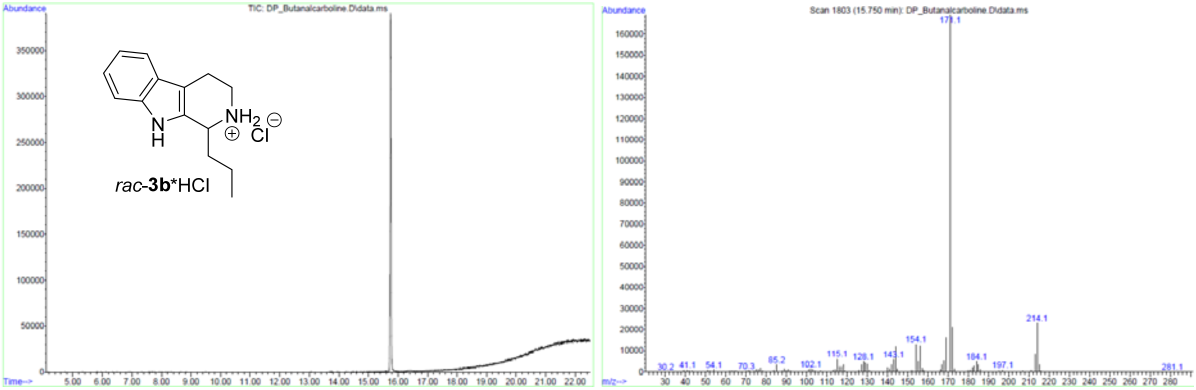

Figure S43. GC-MS spectrum and fragmentation pattern of 1-propyl-2,3,4,9-tetrahydro-1*H*-pyrido[3,4-*b*]indol-2-ium chloride (*rac*-**3b***HCl).

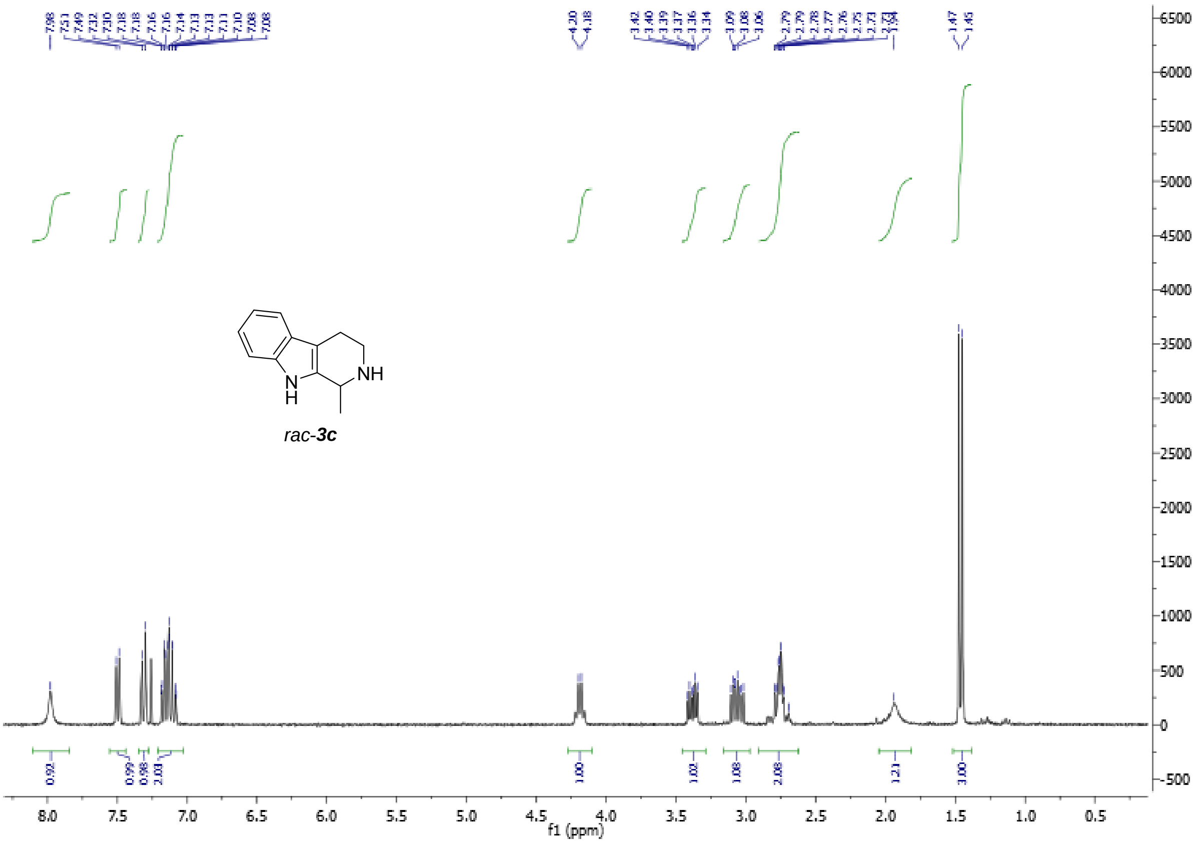

Figure S44. ^{1}H-NMR spectrum of 1-methyl-2,3,4,9-tetrahydro-1*H*-pyrido[3,4-*b*]indole (*rac*-**3c**).

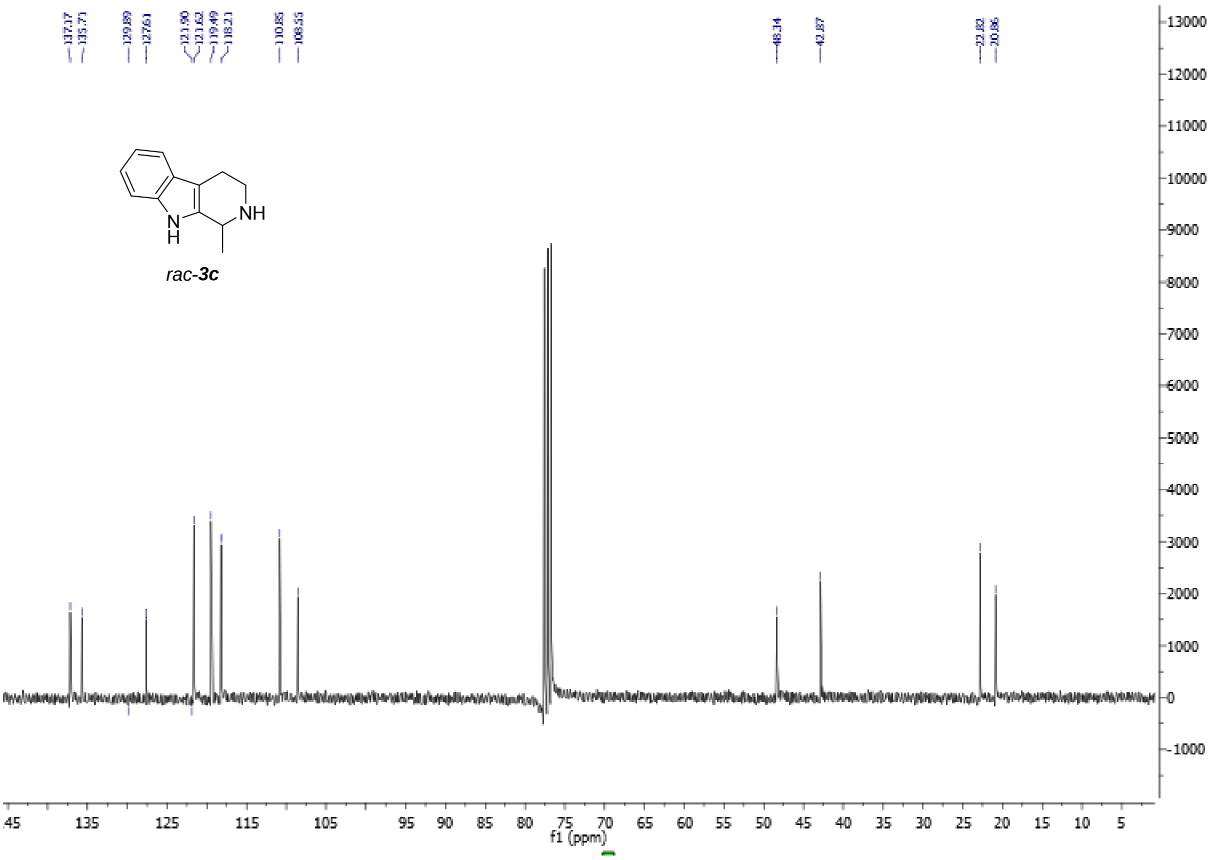

Figure S45. ^{13}C-NMR spectrum of 1-methyl-2,3,4,9-tetrahydro-1*H*-pyrido[3,4-*b*]indole (*rac*-**3c**).

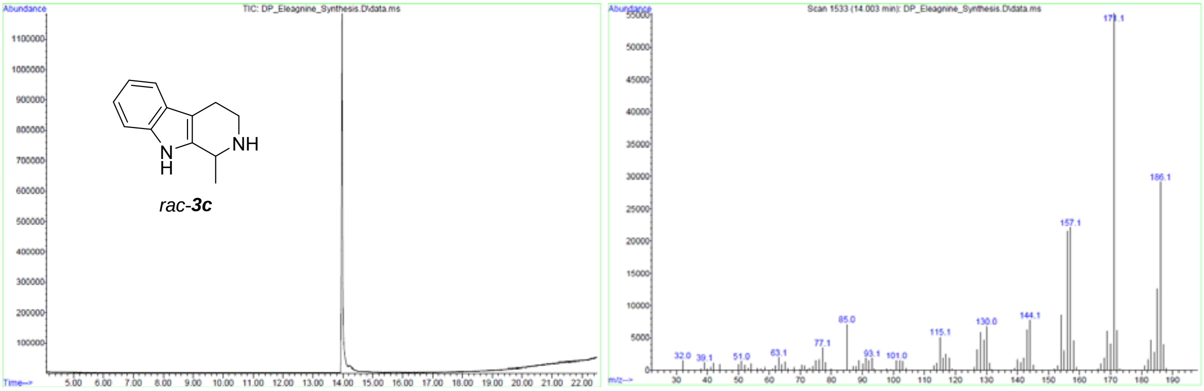

Figure S46. GC-MS spectrum and fragmentation pattern of 1-methyl-2,3,4,9-tetrahydro-1*H*-pyrido[3,4-*b*]indole (*rac*-**3c**).

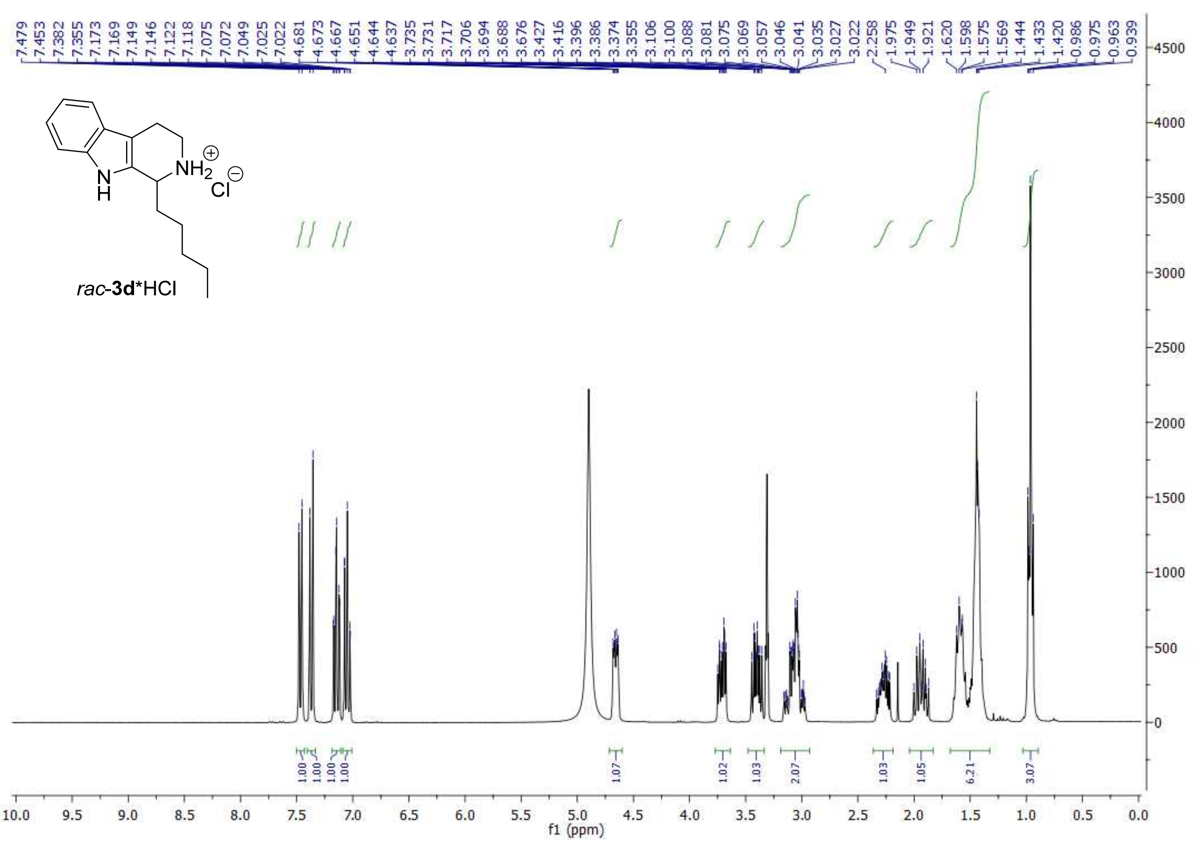

Figure S47. ^{1}H-NMR spectrum of 1-pentyl-2,3,4,9-tetrahydro-1*H*-pyrido[3,4-*b*]indol-2-ium chloride (*rac*-**3d***HCl).

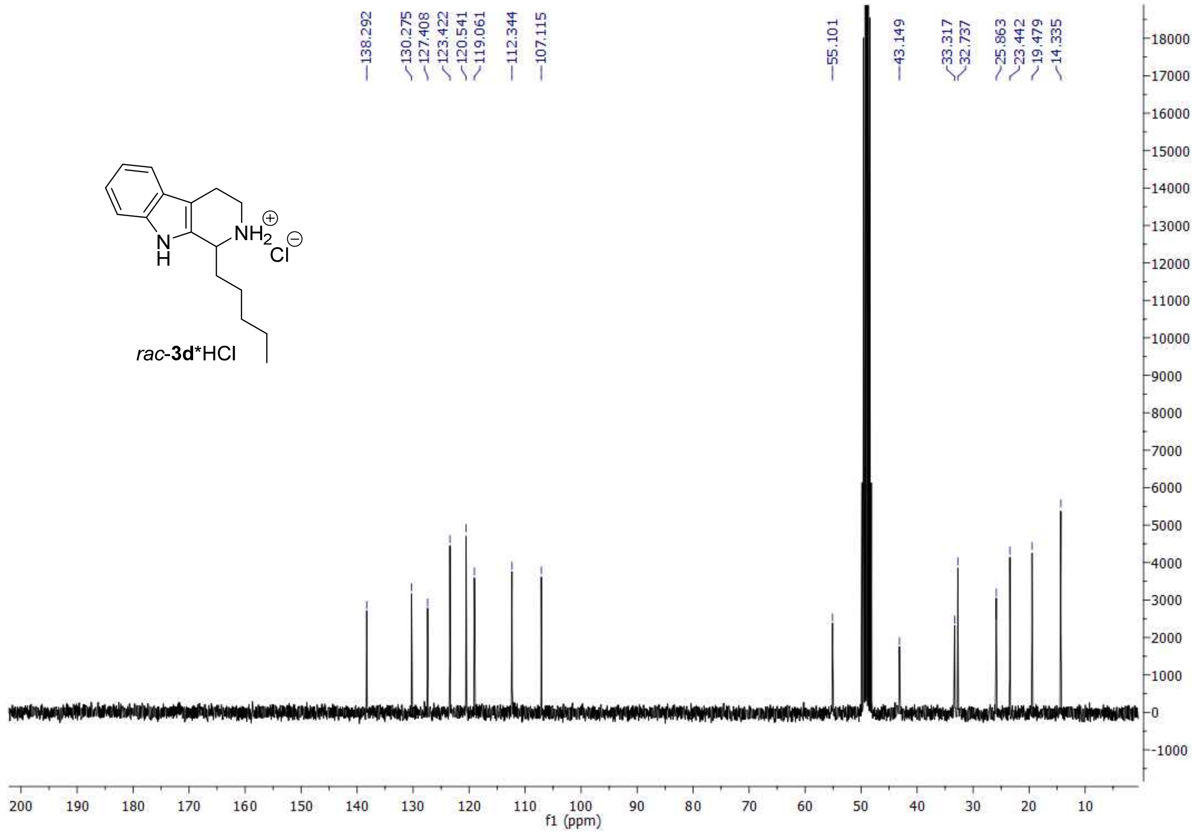

Figure S48. ^{13}C-NMR spectrum of 1-pentyl-2,3,4,9-tetrahydro-1*H*-pyrido[3,4-*b*]indol-2-ium chloride (*rac*-**3d***HCl).

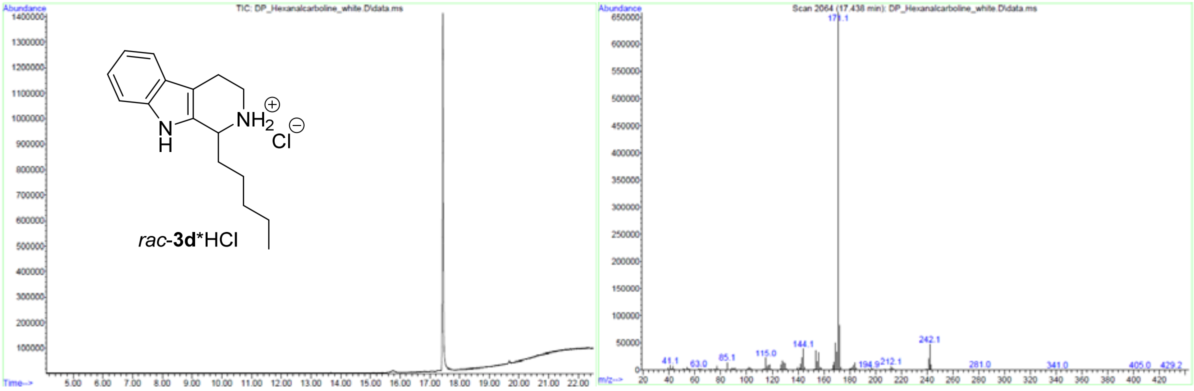

Figure S49. GC-MS spectrum and fragmentation pattern of 1-pentyl-2,3,4,9-tetrahydro-1*H*-pyrido[3,4-*b*]indol-2-ium chloride (*rac*-**3d***HCl).

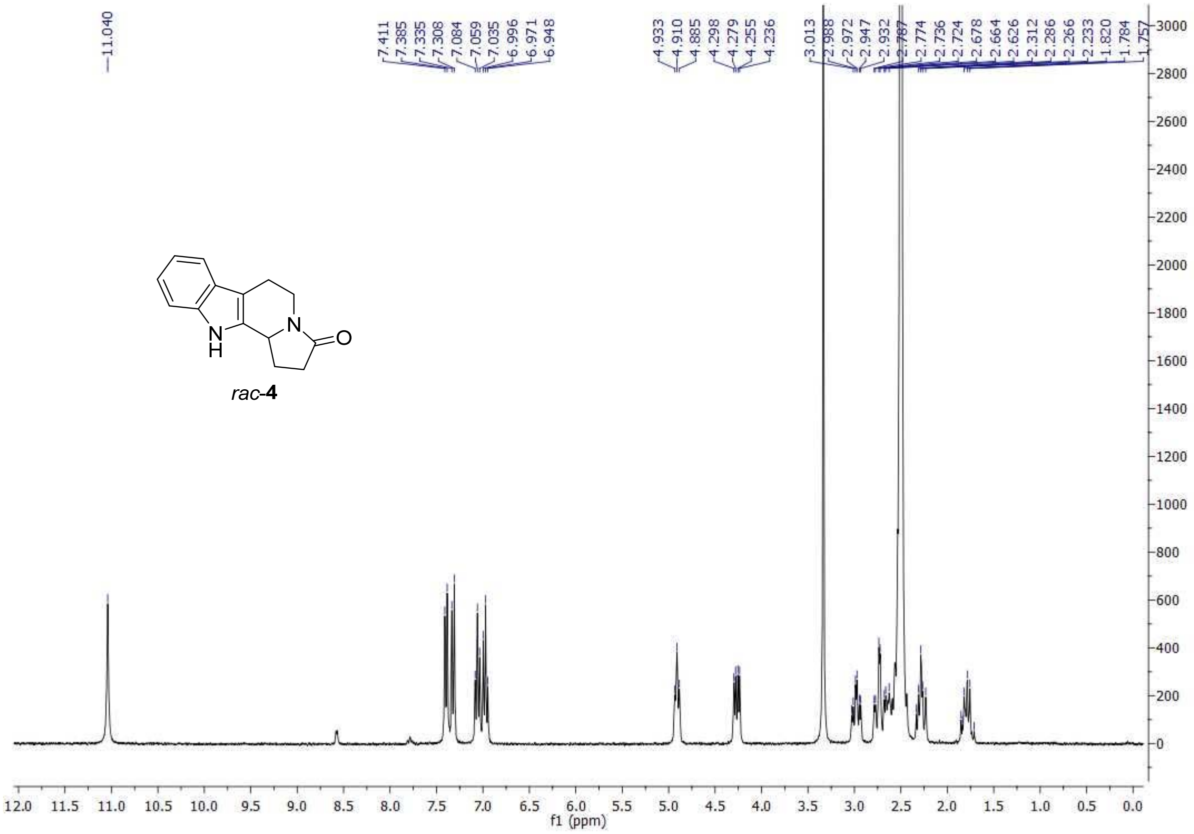

Figure S50. ^{1}H-NMR spectrum of 1,2,5,6,11,11b-hexahydro-3*H*-indolizino[8,7-*b*]indol-3-one (*rac*-**4**).

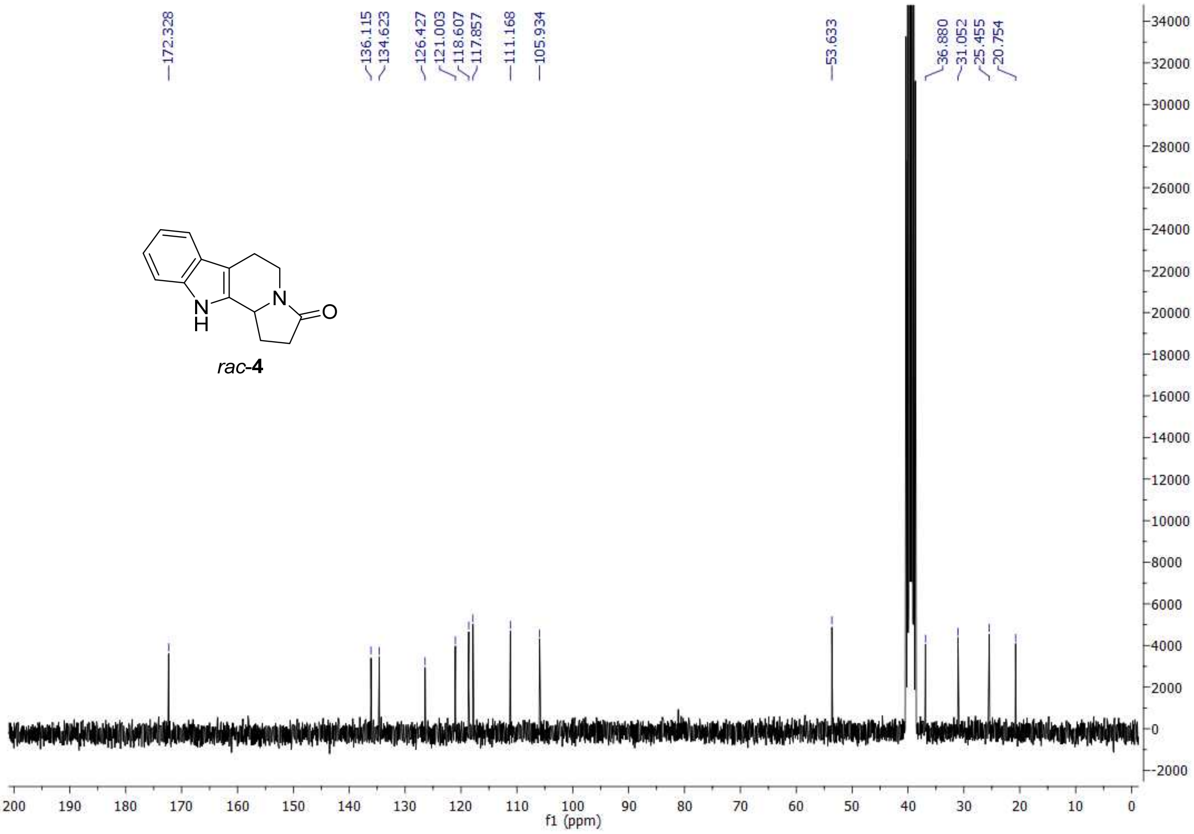

Figure S51. ^{13}C-NMR spectrum of 1,2,5,6,11,11b-hexahydro-3*H*-indolizino[8,7-*b*]indol-3-one (*rac*-**4**).

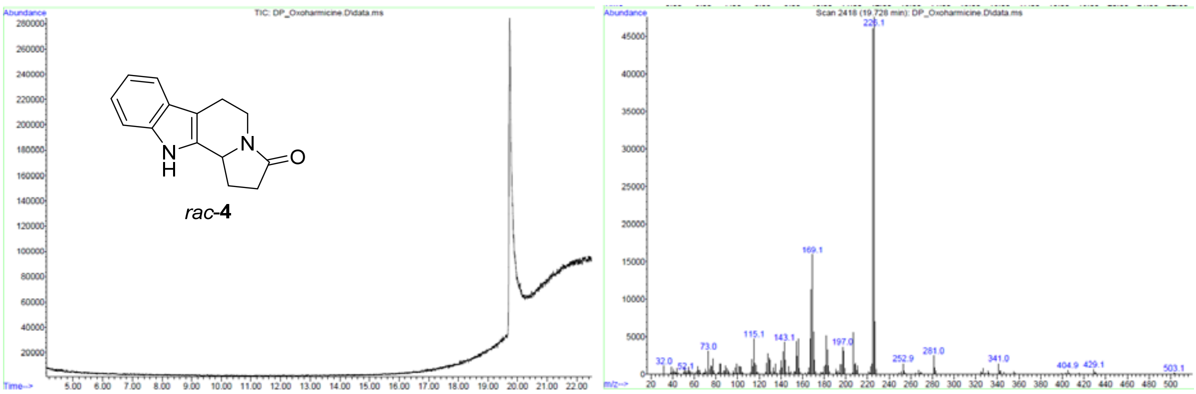

Figure S52. GC-MS spectrum and fragmentation pattern of 1,2,5,6,11,11b-hexahydro-3*H*-indolizino[8,7-*b*]indol-3-one (*rac*-**4**).

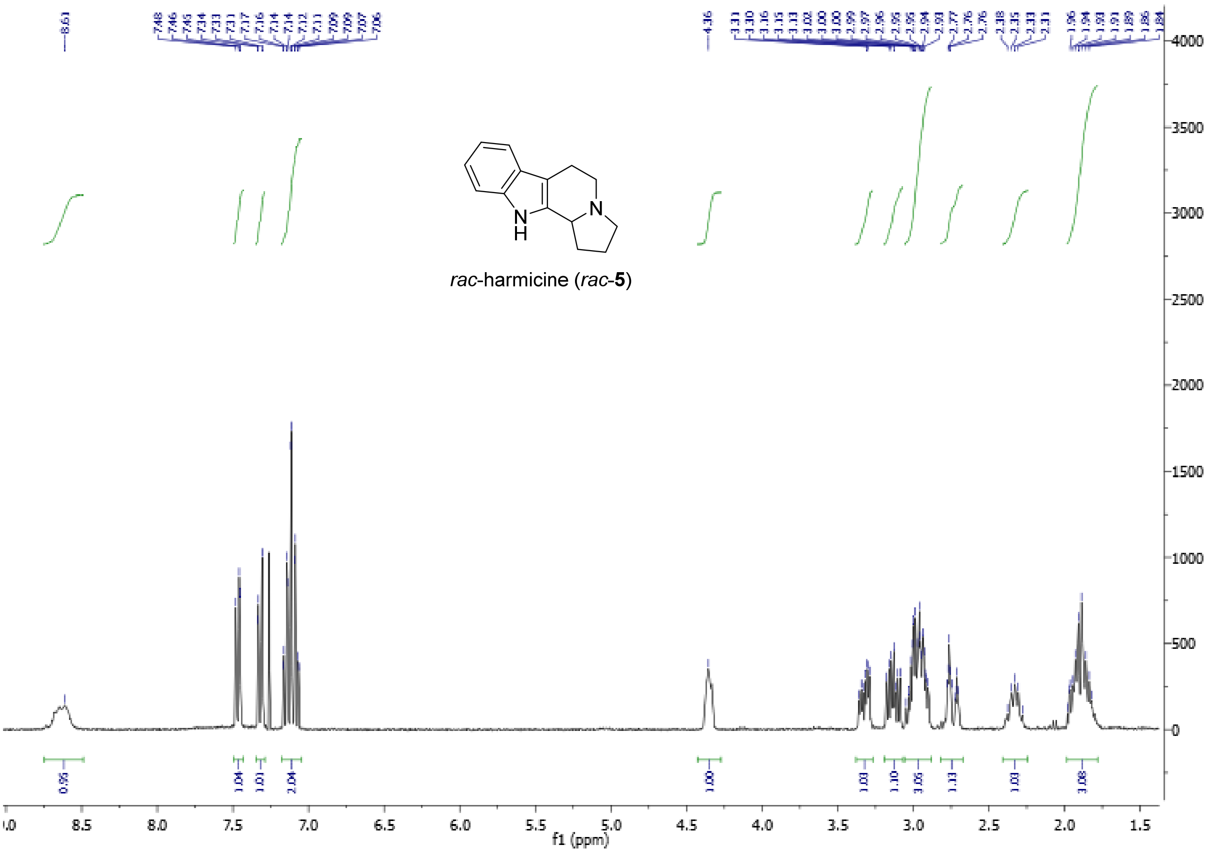

Figure S53. ^{1}H-NMR spectrum of *rac*-harmicine (*rac*-**5**).

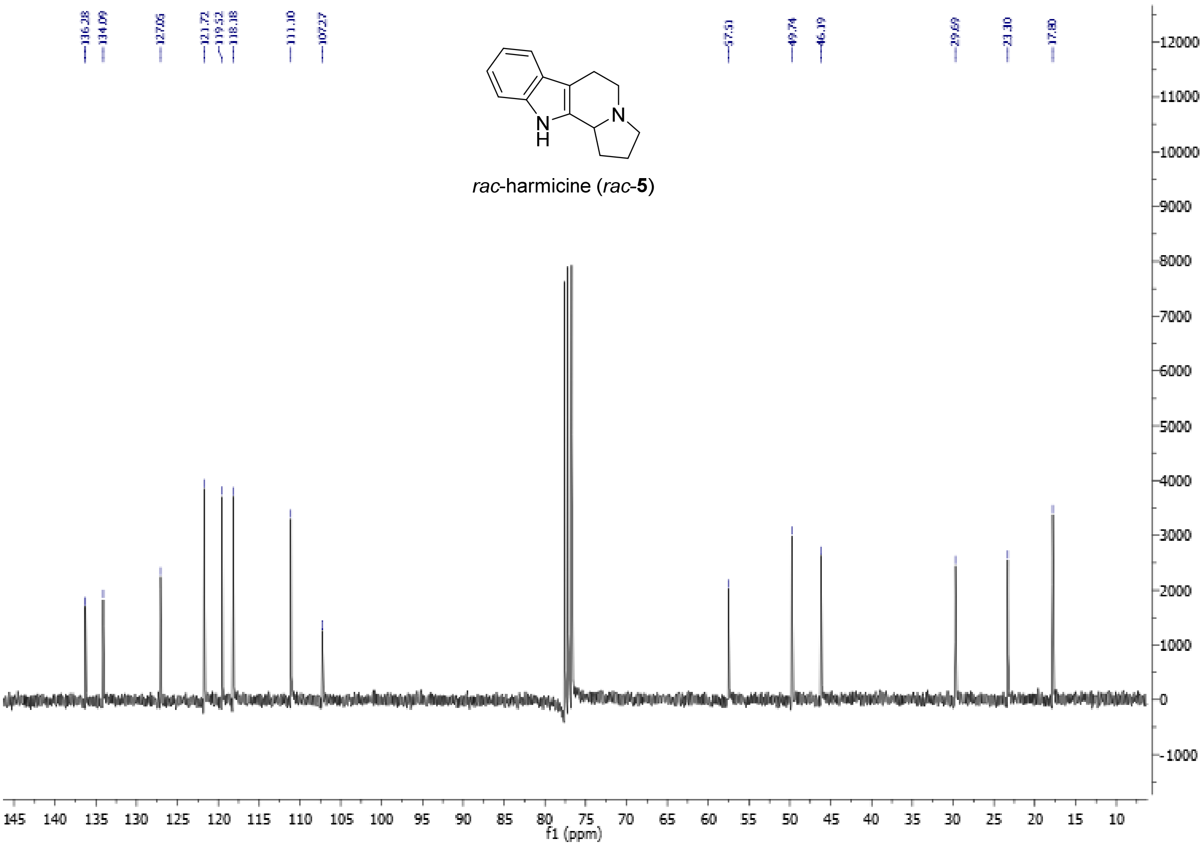

Figure S54. ^{13}C-NMR spectrum of *rac*-harmicine (*rac*-**5**).

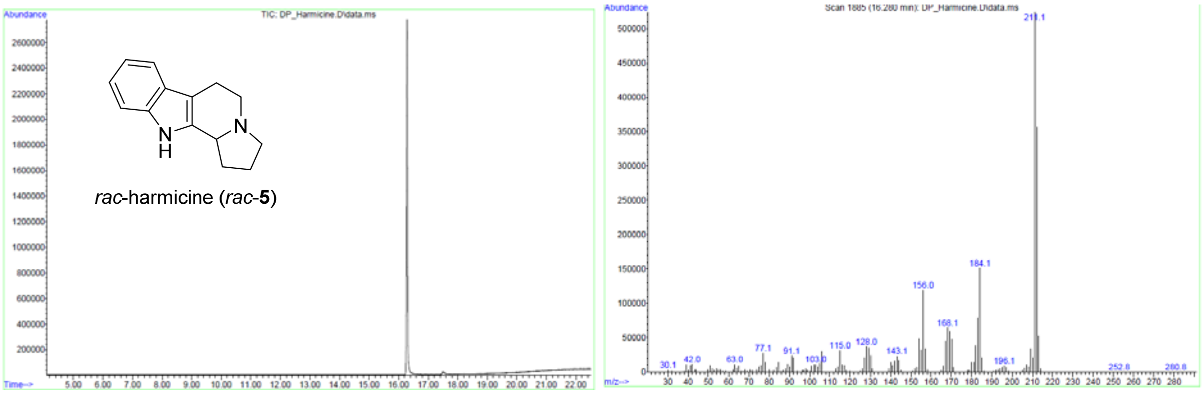

Figure S55. GC-MS spectrum and fragmentation pattern of *rac*-harmicine (*rac*-**5**).

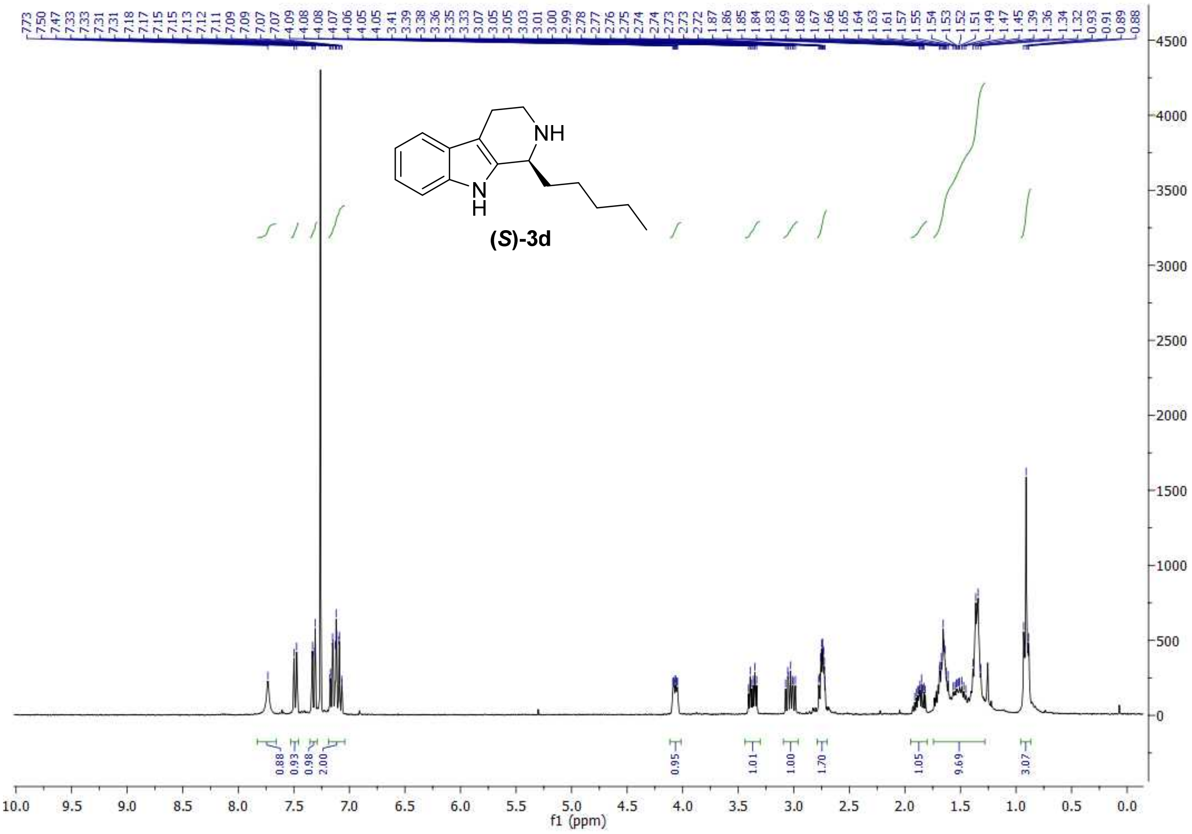

Figure S56. ^{1}H- NMR spectrum of (*S*)-1-pentyl-2,3,4,9-tetrahydro-1*H*-pyrido[3,4-*b*]indole ((*S*)-3**d**) synthesized via the chiral Brønsted acids,approach by Wanner.[7]

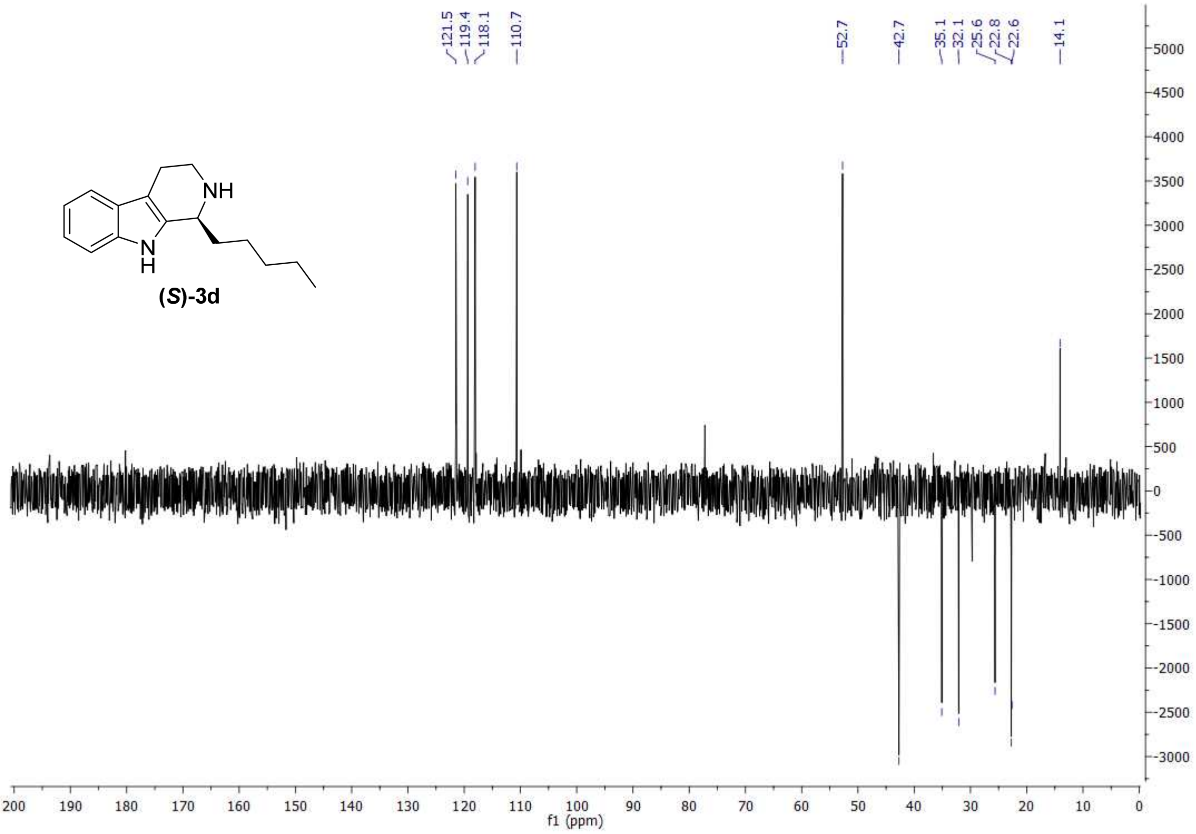

Figure S57. 135-DEPT NMR spectrum of (*S*)-1-pentyl-2,3,4,9-tetrahydro-1*H*-pyrido[3,4-*b*]indole ((*S*)-**3d**) synthesized via the chiral Brønsted acids,approach by Wanner.[7]

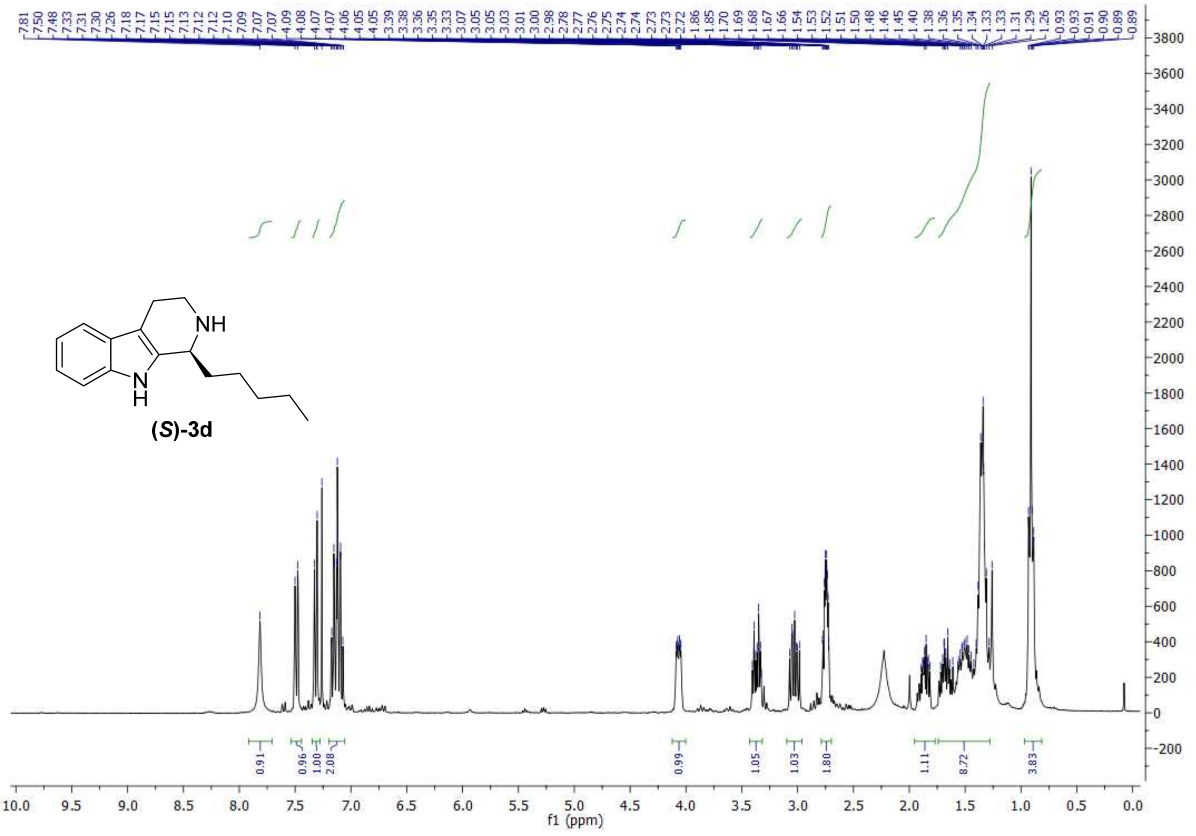

Figure S58. ^{1}H- NMR spectrum of (*S*)-1-pentyl-2,3,4,9-tetrahydro-1*H*-pyrido[3,4-*b*]indole ((*S*)-**3d**) synthesized via the chiral auxiliary method by Gremmen.[8]

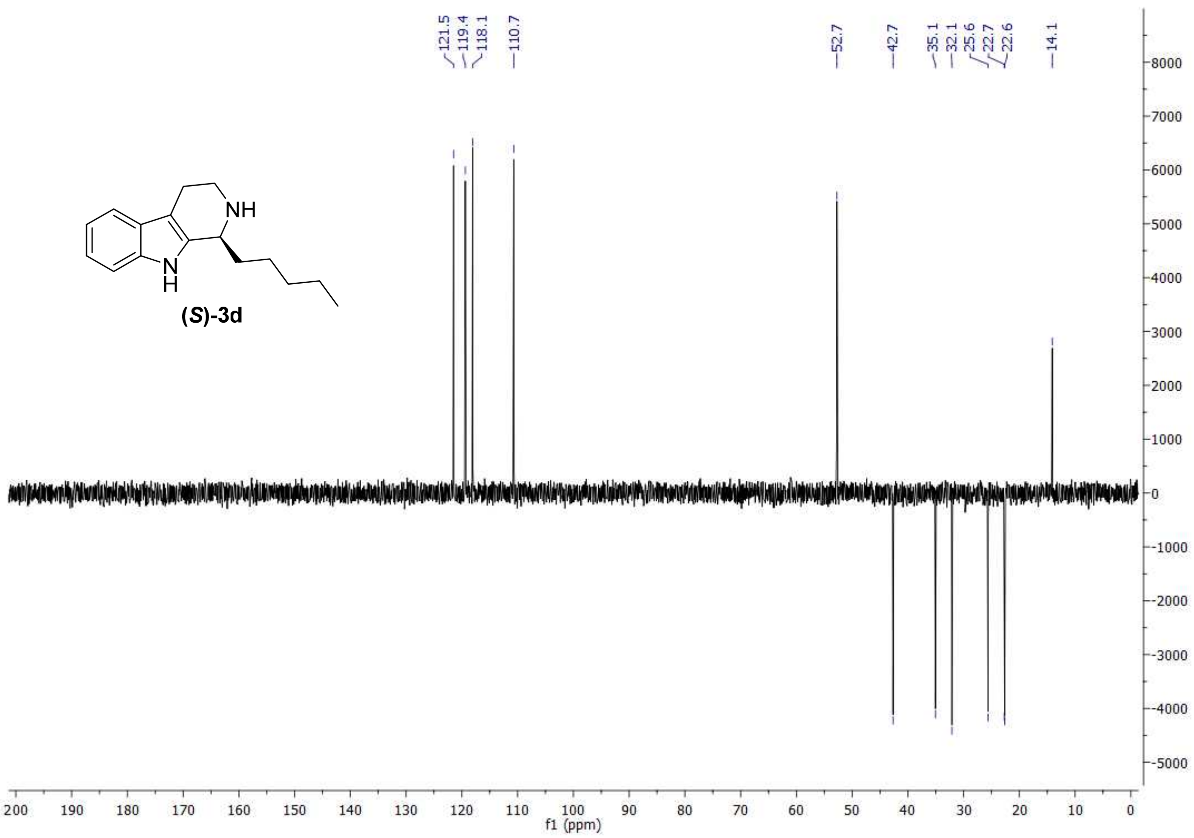

Figure S59. 135-DEPT-NMR spectrum of (*S*)-1-pentyl-2,3,4,9-tetrahydro-1*H*-pyrido[3,4-*b*]indole ((*S*)-**3d**) synthesized via the chiral auxiliary method by Gremmen.[8]

References

[1] E. A. Loris, S. Panjikar, M. Ruppert, L. Barleben, M. Unger, H. Schübel, J. Stöckigt, *Chem. Biol.* **2007**, *14*, 979-985.

[2] P. Bernhardt, A. R. Usera, S. E. O'Connor, *Tetrahedron Lett.* **2010**, *51*, 4400-4402.

[3] D. Ghislieri, D. Houghton, A. P. Green, S. C. Willies, N. J. Turner, *ACS Catal.* **2013**, *3*, 2869-2872.

[4] P. Roszkowski, K. Wojtasiewicz, A. Leniewski, J. K. Maurin, T. Lis, Z. Czarnocki, *J. of Mol. Catal. A: Chem.* **2005**, *232*, 143-149.

[5] W. A. da Silva, M. T. Rodrigues, N. Shankaraiah, R. B. Ferreira, C. K. Z. Andrade, R. A. Pilli, L. S. Santos, *Org. Lett.* **2009**, *11*, 3238-3241.

[6] a) R. S. Heath, M. Pontini, B. Bechi, N. J. Turner, *ChemCatChem* **2014**, *6*, 996-1002; b) S. V. Ryabukhin, D. M. Panov, A. S. Plaskon, A. A. Tolmachev, R. V. Smaliy, *Chem. Month.* **2012**, *143*, 1507-1517.

[7] M. J. Wanner, R. N. S. van der Haas, K. R. de Cuba, J. H. van Maarseveen, H. Hiemstra, *Angew. Chem. Int. Ed.* **2007**, *46*, 7485-7487.

[8] C. Gremmen, B. Willemse, M. J. Wanner, G.-J. Koomen, *Org. Lett.* **2000**, *2*, 1955-1958.
